# MATH-Domain Family Shows Response toward Abiotic Stress in Arabidopsis and Rice

**DOI:** 10.3389/fpls.2016.00923

**Published:** 2016-06-28

**Authors:** Hemant R. Kushwaha, Rohit Joshi, Ashwani Pareek, Sneh L. Singla-Pareek

**Affiliations:** ^1^Microbial Engineering, International Centre for Genetic Engineering and BiotechnologyNew Delhi, India; ^2^Plant Stress Biology, International Centre for Genetic Engineering and BiotechnologyNew Delhi, India; ^3^Stress Physiology and Molecular Biology Laboratory, School of Life Sciences, Jawaharlal Nehru UniversityNew Delhi, India

**Keywords:** abiotic stress, biotic stress, MATH domain, BTB domain, rice, Arabidopsis

## Abstract

Response to stress represents a highly complex mechanism in plants involving a plethora of genes and gene families. It has been established that plants use some common set of genes and gene families for both biotic and abiotic stress responses leading to cross-talk phenomena. One such family, Meprin And TRAF Homology (MATH) domain containing protein (MDCP), has been known to be involved in biotic stress response. In this study, we present genome-wide identification of various members of MDCP family from both Arabidopsis and rice. A large number of members identified in Arabidopsis and rice indicate toward an expansion and diversification of MDCP family in both the species. Chromosomal localization of MDCP genes in Arabidopsis and rice reveals their presence in a few specific clusters on various chromosomes such as, chromosome III in Arabidopsis and chromosome X in rice. For the functional analysis of MDCP genes, we used information from publicly available data for plant growth and development as well as biotic stresses and found differential expression of various members of the family. Further, we narrowed down 11 potential candidate genes in rice which showed high expression in various tissues and development stages as well as biotic stress conditions. The expression analysis of these 11 genes in rice using qRT-PCR under drought and salinity stress identified *OsM4* and *OsMB11* to be highly expressed in both the stress conditions. Taken together, our data indicates that *OsM4* and *OsMB11* can be used as potential candidates for generating stress resilient crops.

## Introduction

Abiotic stress is considered as one of the major factors affecting growth, biomass, and productivity in plants (Singh A. et al., 2015; Joshi et al., 2016a). Among several abiotic stresses, salinity and drought are the key factors for the downfall of yield in the agricultural sector due to reduced productivity in both irrigated and non-irrigated agricultural lands (Gupta et al., 2015). In plants, a high degree of similarity has been reported in salinity and drought stress responses with respect to their physiological, molecular and genetic effects (Joshi et al., 2014). Elevated levels of salt in the soil limits the water uptake because of low water potential, thereby initiating drought stress (Ahmed et al., 2015). It has been well established that osmotic stress in plants triggers turgor loss, membrane disorganization, protein denaturation and production of reactive oxygen species (Joshi et al., 2014). This situation further causes inhibition of photosynthesis, damage of cellular organelles and metabolic dysfunction resulting in growth retardation, reduced fertility, and premature senescence, thus causing severe yield losses (Joshi et al., 2016b). Plants use common pathways and components in response to these stresses (Pastori and Foyer, 2002). Hence, plants tolerant to salinity may also be tolerant to the drought stress or vice-versa (Farooq and Azam, 2001).

Over the years, a number of attempts have been made to improve stress tolerance in crop plants (Singh B. et al., 2015). One of the strategies adopted worldwide for this purpose is the identification of genes that can assist tolerant plants to survive under harsh conditions and using such genes to engineer similar trait in stress sensitive genotypes (Kumar et al., 2012). Though, remarkable progress has been made in developing transgenic plants that can tolerate various stresses (Joshi et al., 2016a), it has been well accepted that these tolerance mechanisms are synchronized by a complex signaling network and orchestrated stress-regulated gene expression (Bohnert et al., 2006; Sreenivasulu et al., 2007; Ramegowda et al., 2014). Thus, identification and characterization of overlapping signal transduction pathways between both salt and drought stresses is essential for getting a holistic view of the response.

One of the major food crops consumed by more than half of the world's population is rice (*Oryza sativa* L.; Bohra et al., 2015). Sensitivity toward abiotic stresses in rice varies with the growth stage, as young seedlings and reproductive stages are highly sensitive to salt and drought stress (Basu and Roychoudhury, 2014). The sensitivity toward these stresses in rice also varies considerably across genotypes. Comparative analysis of various genotypes in rice has been exploited as a successful strategy to discover novel genes and proteins which contribute toward abiotic stress tolerance (Gehan et al., 2015). Earlier, we had employed comparative transcriptomics approach between two contrasting rice genotypes to identify salinity tolerance related genes (Kumari et al., 2009). By employing subtractive hybridization using two contrasting rice genotypes, Pokkali (salt tolerant) and IR64 (salt sensitive), a total of 1194 ESTs (584 from IR64 and 610 from Pokkali) were identified. Analysis of these ESTs led to the identification of various novel genes playing a possible role in salt stress specific response. In fact, an EST identified from this study led to the characterization of CDCP genes in Arabidopsis and rice (Kushwaha et al., 2009; Singh et al., 2012). Another protein identified from the analysis of these ESTs is the MATH (Meprin And TRAF Homology) domain containing protein (MDCP) which has been analyzed in the present study.

Biotic stress is also reported to contribute to 50–80% yield loss in the absence of control measures (Foyer et al., 2016). Previously, available data on biotic stress along with the changing climatic conditions project toward an increase in reproductive potential and geographical expansion of different pathogen strains with higher chances of plants encountering biotic stresses in future (Kissoudis et al., 2014). MDCPs were earlier known for their role in the plant/microbe interaction. They are the early responsive membrane bound receptor kinases reported in Arabidopsis, which gets transiently up-regulated during the fungal interaction, and decrease thereafter when the interaction is established (Peškan-Berghöfer et al., 2004; Shahollari et al., 2005). The TRAF-C domain of TRAF protein and C-terminal region of meprin A and B constitutes the MATH domain of MDCPs (Sunnerhagen et al., 2002). Meprins are tissue-specific and membrane-associated oligomeric zinc endopeptidases that belong to the Astacin family of Metzincin superfamily. These are the largest extracellular proteases in the animal system which cleaves various peptides including growth factors, cytokines and extracellular matrix proteins (Broder and Becker-Pauly, 2013). Tumor necrosis factor-Receptor Associated Factors (TRAFs) belong to the adaptor protein family, and are characterized by a carboxy-terminal homology domain of about 180 amino acids, forming 7–8 antiparallel β-sheets defined as TRAF domain (TD) (Bradley and Pober, 2001; Zapata et al., 2007; Zhou et al., 2015). They are the key factors of the Toll-Like Receptor (TLR) family and Tumor Necrosis Factor (TNF) family, which regulate downstream signaling pathways and finally activate various transcription factors related to cell survival and stress responses (Huang B. et al., 2016). It also triggers the downstream components of signaling pathways, controls the sub-cellular localization of the receptor-ligand complexes, and modifies the response by controlling the degradation of proteins (Zapata et al., 2007). Recently, two redundant TRAF proteins were identified which play a role in the turnover of the nucleotide-binding domain and leucine-rich repeat-containing (NLR) immune receptors SNC1 and RPS2 (Huang S. et al., 2016).

Various other sets of protein domains such as peptidases, RING and zinc finger, filamin and RluA domains, BTB (Broad-complex, Tramtrack, and Bric a brac) domain, tripartite motif (TRIM) and astacin domains are known to be present in conjunction with the MATH domain (Zapata et al., 2007). The number of MDCPs in Arabidopsis and *Brassica rapa* have been found to be similar to *C. elegans* but their role in plants is still unknown (Oelmüller et al., 2005; Zhao et al., 2013). These MATH domain containing proteins have been hypothesized for having a role in the regulation of protein processing (Zapata et al., 2007). The MATH-BTB proteins have been in fact found to play a role in ABA signaling (Lechner et al., 2011). Further, MDCPs are reported to localize in various subcellular compartments such as endoplasmic reticulum, Golgi apparatus, cytosol, nucleus, and organellar membranes, especially peroxisomes.

In the present study, we have identified and characterized MDCP-encoding gene family members in Arabidopsis and rice. A detailed comparison has been made in terms of phylogeny and their genome organization. Expression profile for all the MDCP family members in various tissues, developmental stages as well as biotic and abiotic stress conditions has been studied using the publicly available database. Further, eleven biotic stress-responsive MDCP encoding genes have been analyzed for their expression under salt and drought stress by qRT-PCR. Based on the analysis presented here, we have highlighted the possible role of MDCP-encoding gene family members in both biotic and abiotic stress response in plants.

## Materials and methods

### Identification of MDC proteins

The MDC protein sequences were fetched and classified using *Arabidopsis* (TAIR release 10.0; Berardini et al., 2015) and *Oryza sativa* (TIGR release 7.0; Kawahara et al., 2013) whole genome sequences. Profiles unique to the MATH domain (accession no. PF00917) were obtained from Pfam database (Finn et al., 2014) and were used to screen the whole genome protein sequences of both Arabidopsis and rice, using the HMMER software (version 3.0) deploying default parameters (Eddy, 1998). The protein sequences obtained from the profile search were manually checked for the presence of additional domains along with the MATH domain. We have assigned names to these protein sequences following the domains observed in the individual protein sequences, where “At” denote *Arabidopsis thaliana* and “Os” denote *Oryza sativa*. This is followed by a number of times the MATH “M” or BTB “B” domains are present in the sequence.

### Analysis of MDC proteins

Further, the protein sequences identified were analyzed for their characteristics such as pI, molecular weight using TAIR (release 10.0; Berardini et al., 2015) and TIGR (release 7.0; Kawahara et al., 2013) for Arabidopsis and rice respectively. The subcellular localization of the MDC proteins of Arabidopsis was predicted based on SUBA database (http://suba3.plantenergy.uwa.edu.au/) while that of rice was predicted using subCELlular LOcalization predictor (CELLO v. 2.5: http://cello.life.nctu.edu.tw/) (Yu et al., 2006) and re-confirmed using WoLF PSORT, an advanced protein subcellular localization prediction tool (http://www.genscript.com/wolf-psort.html) (Horton et al., 2007; Nielsen, 2016).

### Chromosomal localization of MDCP encoding genes and phylogenetic analysis

In order to identify the localization of MDCP encoding genes on various chromosomes we used publicly available information resources, that is, TAIR for Arabidopsis and TIGR for rice. The chromosomal positions were plotted using Dia diagram editor (Dia 0.97.2). The rooted ML tree was build using PhyML 3.0 (Guindon et al., 2010) and the final tree was plotted using FigTree 1.4.2 (Rambaut, 2012). To build phylogenies, bootstrap analysis was conducted using 1000 replicates. The sequence analysis was performed using Seaview (version 4) multiple sequence alignment editor (Gouy et al., 2010).

### *In silico* gene expression analysis

Expression pattern for each gene model of MATH domain encoding genes were analyzed in different tissues (such as, callus, seedling, coleoptiles, root, inflorescence, panicle, spikelet, stamen, anther, pollen, stigma, ovary, caryopsis, embryo, endosperm, culm, node, internode, stele, pith, parenchyma, peduncle, leaf, blade, sheath, flag leaf, collar, rhizome, primary root, and root tip; Table S1), at various developmental stages (such as, germination, seedling, tillering, stem elongation, booting, heading, flowering, milk, and dough; Table S2), and under different abiotic stresses (such as, cold, drought, heat, and salinity; Table S3), and biotic stresses (Table S4) were obtained from Affymetrix GeneChip database using Response Viewer (https://www.genevestigator.com) (Hruz et al., 2008). For Arabidopsis, 22 K ATH1 genome array was chosen and pre-existing microarray data of Arabidopsis was considered for further analysis. In the case of rice, microarray datasets of OS_51 K: Rice Genome 51 K array were analyzed.

Further, the same dataset was used for analysis under various biotic stresses i.e., various nematodes and insect pests in rice. In Arabidopsis, various mutants were analyzed along with their response to various bacterial elicitors. The expression of MDC proteins in Arabidopsis was also analyzed in response to various bacterial and fungal infections.

### Plant material and stress treatments

Seeds of *Oryza sativa* ssp. *indica*, cv. IR64 were surface sterilized with bavistin solution (0.1%), rinsed with distilled water and germinated hydroponically in half strength Yoshida medium as described previously (Mustafiz et al., 2011). Seedlings were grown under 16 h/8 h photoperiod at 28 ± 2°C with 70% humidity in the growth chamber (Panasonic, Japan). Ten day old seedlings were subjected to various stress treatments for 6 h (Tripathi et al., 2012). For salinity stress, seedlings were supplemented with half strength Yoshida medium containing 200 mM NaCl and for drought stress, seedlings were air-dried between folds of tissue paper as described (Singh V. K. et al., 2015). Untreated seedlings grown in half strength Yoshida medium were taken as control. The shoot tissues were harvested and immediately frozen in liquid nitrogen and stored at −80°C for RNA isolation.

### Quantitative real-time PCR analysis

Total RNA was isolated from shoot tissues using TRIzol reagent (Thermo Fisher Scientific, USA) according to the manufacturer's protocol. RNA quality and integrity was determined using NanoDrop spectrophotometer and agarose gel electrophoresis. Total RNA was treated with 2 μg of DNase (Thermo Fisher Scientific, USA) and reverse transcribed with RevertAid® RNase H minus cDNA synthesis kit (Thermo Fisher Scientific, USA) according to the manufacturer's instructions. Using Primer Express Software v3.0 (Applied Biosystems, USA), the primers for qRT-PCR analysis were designed from the 3′-UTR region of the selected genes (Table S5). The specificity of amplification was further confirmed by Primer-BLAST (http://www.ncbi.nlm.nih.gov/tools/primer-blast/). The qRT-PCR assay was performed in 20 μl final reaction mixture according to the instructions for Power SYBR® Green PCR Master Mix (Applied Biosystems, USA) using 7500^TM^ Real-Time PCR system and software (Applied Biosystems, USA). The reaction was performed using three biological and three technical replicates as follows: 95°C for 10 min followed by 40 cycles of 95°C for 15 s and 60°C for 1 min. Elongation factor 1-α (eEf-1α) was used as reference gene for normalization (Tripathi et al., 2015). Dissociation curve analysis and gel electrophoresis was carried out to check the specificity of amplification. Relative change in fold expression was calculated using comparative CT value (Livak and Schmittgen, 2001) and two-tailed Student's *t*-test was used to analyze statistical significance at *p* < 0.05.

## Results

### Identification and characterization of MDC proteins

To identify the MDC proteins in Arabidopsis and rice, the profile of MATH domain (accession no. PF00917) was obtained from the Pfam database using HMM-based method (see Materials and Methods). The method used for the identification of MDC proteins remains same as used earlier for the identification and classification of various other gene families such as TCS (Pareek et al., 2006; Singh A. et al., 2015), CDCP (Kushwaha et al., 2009), glyoxalase I and II (Mustafiz et al., 2011), cyclophilins (Kumari et al., 2015), NCX (Singh A. K. et al., 2015), histone chaperones (Tripathi et al., 2015), and glyoxalase III (Ghosh et al., 2016).

Genome-wide analysis search of MDC proteins revealed the presence of 62 MDC genes coding for 82 proteins in Arabidopsis. Similarly, in rice, 69 genes were found to be coding for 74 MDC proteins. Classification of these proteins was based on the presence of MATH domain either as a single domain or multiple domains or along with BTB domain (Accession No. PF00651; Figure S1). The POZ (POxvirus and Zinc finger) domain, renamed as BTB (Broad-Complex, Tramtrack, and Bric à brac) domain is evolutionarily conserved and plays a role in the regulation of gene expression through protein-protein interactions (Ahmad et al., 1998). The proteins having MATH domain have been named as “M” (for the single MATH domain), “2M” (for two MATH domains), “3M” (for three MATH domains), “4M” (for four MATH domains), “MB” (for single MATH and single BTB domain), and “2M2B” (for two MATH along with two BTB domains) followed by a number which represents the sequence order in which they were found in the search. Each name is preceded by the name of the species in which they were identified such as, “At” representing Arabidopsis and “Os” representing *Oryza sativa*. Further, the postscript alphabets were assigned like “a,” “b” etc for representing the alternative splice proteins in both the species.

In Arabidopsis, 39 single domain proteins were encoded by 28 genes, while in rice, 13 such genes code for 15 proteins (Table 1). In the group of proteins having two MATH domains, 25 genes in Arabidopsis were found to code 31 proteins, while in rice, only a single such instance was observed. Only 2 proteins, encoded by 2 genes in Arabidopsis were found to possess three MATH domains and only 1 protein possessed 4 MATH domains. However, in rice, no protein was identified having 3 or 4 MATH domains.

**Table 1:** **Comparison of MDC protein members and their encoding genes in Arabidopsis and rice**.

	**Arabidopsis**		**Rice**	
	**Gene**	**Protein**	**Gene**	**Protein**
Single MATH domain	28	39	13	15
Single MATH + Single BTB domain	06	09	54	57
Two MATH domain	25	31	01	01
Two MATH + Two BTB domain	–	–	01	01
Three MATH domain	02	02	–	–
Four MATH domain	01	01	–	–

The alternative splicing mechanism has been considered as the major source of diversity and complexity in various species (Brett et al., 2002; Ghosh et al., 2016). In Arabidopsis, 15 instances of alternative splicing have been observed generating 35 MDC proteins (Table 2) while in rice, 9 MDC proteins have been observed as a result of four alternative splicing events (Table 3).

**Table 2:** **MDC protein members of Arabidopsis**.

**Gene**	**Proteins**	**Locus**	**Coordinate 5′–3′**	**AA**	**Subcellular localization**	**MW (Da)**	**pI**
**ONE MATH DOMAIN**							
*AtM1*	AtM1a	AT1G04300.1	1,148,818–1,153,895	1074	NUC	119,653.9	6.3922
	AtM1b	AT1G04300.2	1,148,818–1,153,095	997	NUC	111,017.2	7.1375
	AtM1c	AT1G04300.3	1,148,818–1,153,895	1082	NUC	120,442.8	6.3267
	AtM1d	AT1G04300.4	1,148,818–1,153,895	1055	NUC	117,429.3	6.2591
*AtM2*	AtM2	AT1G65370.1	24,284,707–24,285,699	227	NUC	25,637.1	5.1422
*AtM3*	AtM3	AT1G31390.1	11,243,191–11,244,392	268	CYT	30,716.9	4.7923
*AtM4*	AtM4	AT1G69660.1	261,99,623–26,200,603	231	NUC	26,769.2	6.1501
*AtM5*	AtM5	AT1G65050.1	24,164,286–24,165,679	228	MIT	25,855.2	6.7984
*AtM6*	AtM6	AT2G01790.1	341,322–342,480	269	CHL	30,514.6	4.8414
*AtM7*	AtM7a	AT2G42460.1	17,676,399–17,679,247	442	CYT	50,287.0	9.6502
	AtM7b	AT2G42460.2	17,678,018–17,679,247	299	CYT	34,203.4	9.8068
*AtM8*	AtM8	AT2G05420.1	1,983,901–1,985,341	297	CYT	33,642.5	9.9165
*AtM9*	AtM9	AT2G05410.1	1,977,490–1,978,553	265	NUC	30,213.7	6.9302
*AtM10*	AtM10a	AT3G11910.1	3,761,758–3,770,290	1115	CYT	130,647.9	5.2677
	AtM10b	AT3G11910.2	3,761,758–3,770,290	1114	CHL	130,519.8	5.2677
*AtM11*	AtM11	AT3G58210.1	21,562,645–21,564,067	330	CYT	37,798.2	5.7367
*AtM12*	AtM12a	AT3G58290.1	21,580,572−21,581,861	282	CYT	32,361.5	4.6457
	AtM12b	AT3G58290.3	21,580,572−21,581,861	264	CYT	30,236.2	4.8263
*AtM13*	AtM13a	AT3G58220.1	21,565,173−21,566,435	351	NUC	40,883.1	4.7638
	AtM13b	AT3G58220.2	21,564,677−21,566,435	453	NUC	52,614.4	4.6927
*AtM14*	AtM14	AT3G58250.1	21,570,745−21,572,143	317	CHL	36,182.2	5.6895
*AtM15*	AtM15	AT3G44790.1	16,328,792−16,330,265	324	CHL	37,262.6	8.1007
*AtM16*	AtM16	AT3G58360.1	21,593,505−21,594,866	298	CHL	34,496.2	5.1936
*AtM17*	AtM17	AT3G58440.1	21,618,446−21,621,249	601	CHL	67,401.7	4.2072
*AtM18*	AtM18	AT3G58200.1	21,560,086−21,561,358	319	CHL	36,860.5	5.4114
*AtM19*	AtM19	AT3G58410.1	21,604,871−21,606,229	328	EXT	37,986.4	4.6825
*AtM20*	AtM20	AT3G29580.1	11,394,675−11,395,871	306	CYT	35,040.2	5.5592
*AtM21*	AtM21	AT3G58350.1	21,591,618−21,592,836	301	NUC	34,071.7	5.302
*AtM22*	AtM22	AT3G44800.1	16,343,333−16,346,027	564	CYT	63,853.6	8.6223
*AtM23*	AtM23	AT4G16045.1	9,089,906−9,091,860	382	NUC	44,311.3	5.2049
*AtM24*	AtM24a	AT5G52330.1	21,247,596−21,249,732	397	CYT	46,773.6	7.5279
	AtM24b	AT5G52330.2	21,247,596−21,249,288	346	CYT	40,861.9	7.1561
*AtM25*	AtM25	AT5G26300.1	9,229,326−9,231,033	349	EXT	39,390.2	7.1582
*AtM26*	AtM26a	AT5G43560.1	17,501,043−1,750,5526	1055	NUC	117,448.5	6.8912
	AtM26b	AT5G43560.2	17,501,043−1,750,5526	1055	NUC	117,448.5	6.8912
*AtM27*	AtM27a	AT5G06600.1	2,020,682−2,027,834	1116	CYT	130,606.1	5.5325
	AtM27b	AT5G06600.2	2,019,545−2,027,834	1115	CYT	130,477.9	5.5325
	AtM27c	AT5G06600.3	2,020,682−2,027,834	985	CYT	115,150.8	5.4092
*AtM28*	AtM28	AT2G04170.5	1,417,660−1,419,156	369	MIT	38,976.4	10.1232
**ONE MATH** + **ONE BTB DOMAIN**							
*AtMB1*	AtMB1a	AT5G19000.1	6,342,563–6,344,641	407	CHL	44,728.7	7.286
	AtMB1b	AT5G19000.2	6,342,563–6,344,641	442	CHL	48,583.3	7.7142
*AtMB2*	AtMB2	AT5G21010.1	7,136,062–7,138,374	410	CHL	45,190.9	6.5729
*AtMB3*	AtMB3a	AT2G39760.1	16,583,213–16,585,983	408	NUC	44,889.8	7.2209
	AtMB3b	AT2G39760.2	16,583,213–16,584,815	343	NUC	37,718.7	5.1807
*AtMB4*	AtMB4	AT3G43700.1	15,601,944–15,603,499	415	CHL	45,747.3	7.3394
*AtMB5*	AtMB5a	AT3G06190.1	1,874,577–1,876,575	406	NUC	45,158.2	7.103
	AtMB5b	AT3G06190.2	1,874,577–1,876,575	295	NUC	32,533.5	8.2031
*AtMB6*	AtMB6	AT3G03740.1	937,106–939,807	465	CYT	50,997.5	4.9782
**Two MATH Domain**							
*At2M1*	At2M1	AT1G69650.1	26,197,498–26,198,821	294	CHL	33,717.3	9.5888
*At2M2*	At2M2	AT1G58270.1	21,612,394–21,614,089	396	VAC	45,035.2	5.5264
*At2M3*	At2M3a	AT1G65150.1	24,204,167–24,205,558	296	MIT	33,583.6	6.44
	At2M3b	AT1G65150.2	24,204,167–24,205,558	296	MIT	33,583.6	6.44
*At2M4*	At2M4	AT2G15710.1	6,842,648–6,845,103	365	NUC	42,558.1	5.861
*At2M5*	At2M5	AT2G42470.1	17,679,887–17,685,187	898	CHL	103,071.4	6.8265
*At2M6*	At2M6a	AT2G04170.1	1,417,404–1,419,156	420	MIT	44,404.4	9.8464
	At2M6b	AT2G04170.2	1,417,404–1,419,156	420	MIT	44,404.4	9.8464
	At2M6c	AT2G04170.3	1,417,404–1,418,711	298	CYT	33,875.9	7.7519
	At2M6d	AT2G04170.4	1,417,404–1,418,711	298	CYT	33,875.9	7.7519
*At2M7*	At2M7	AT2G42480.1	17,685,805–17,689,851	743	NUC	86,420.6	5.0429
*At2M8*	At2M8	AT2G32880.1	13,948,953–13,950,505	318	NUC	36,728.1	9.1903
*At2M9*	At2M9	AT2G32870.1	13,944,968–13,946,776	416	CYT	48,326.1	9.9181
*At2M10*	At2M10	AT2G04190.1	1,427,594–1,430,230	411	CYS	44,418.2	6.59
*At2M11*	At2M11	AT3G17380.1	5,950,240–5,952,124	309	CYS	35,126.4	7.0092
*At2M12*	At2M12	AT3G28220.1	10,524,420–10,526,497	370	CYT	42,886.5	8.8
*At2M13*	At2M13	AT3G20360.1	7,099,952–7,101,589	363	CHL	41,763.4	9.2958
*At2M14*	At2M14	AT3G46190.1	16,965,889−16,967,345	291	PER	33,425.1	4.3038
*At2M15*	At2M15	AT3G20370.1	7,105,481–7,107,079	379	CHL	43,448.8	6.5366
*At2M16*	At2M16	AT3G20380.1	7,108,183–7,109,770	375	CHL	43,157.6	8.9212
*At2M17*	At2M17	AT3G27040.1	9,974,912–9,977,927	358	NUC	41,264.8	9.3241
*At2M18*	At2M18	AT4G09780.1	6,159,538–6,161,378	427	CHL	49,656.1	9.2725
*At2M19*	At2M19a	AT4G09770.1	6,154,534–6,155,859	297	CHL	34,199.7	7.7039
	At2M19b	AT4G09770.2	6,154,534–6,155,859	297	CHL	34,199.7	7.7039
*At2M20*	At2M20	AT4G00780.1	334,779–336,120	299	CYT	34,323.1	7.4232
*At2M21*	At2M21	AT4G01390.1	570,242–571,595	300	CHL	34,272.0	8.1505
*At2M22*	At2M22	AT5G26260.1	9,200,492–9,202,153	351	VAC	39,826.0	9.6237
*At2M23*	At2M23a	AT5G26280.1	9,208,724–9,210,403	350	CHL	39,445.2	8.9246
	At2M23b	AT5G26280.2	9,208,724–9,210,403	327	CHL	36,874.4	8.576
*At2M24*	At2M24	AT5G26290.1	9,226,079–9,227,873	333	CHL	37,690.7	9.2797
*At2M25*	At2M25	AT5G26320.1	9,238,310–9,241,236	352	EXT	39,999.0	7.0751
**THREE MATH DOMAIN**							
*At3M1*	At3M1	AT2G25330.1	10,788,946–10,791,331	693	NUC	78,068.1	4.8947
*At3M2*	At3M2	AT2G25320.1	10,781,951–10,788,065	1673	NUC	187,674.3	5.3989
**FOUR MATH DOMAIN**							
*At4M1*	At4M1	AT3G22080.1	7,777,818–7,781,718	648	CYS	74,469.1	8.154

*The genes and their respective proteins have been prefixed by “At.” The alternative spliced forms have been postfixed with the alphabets like “a,” “b” and so on. The table shows their predicted sub-cellular localization like (CHL), Chloroplast; (CYT), Cytoplasm; (NUC),Nucleus; (VAC), Vacuole; (MIT), Mitochondria; (EXT), Extracellular; (CYS), Cytoskeleton; or (PER), Peroxisome; along with their (MW), molecular weight; in (Da), Dalton; and pI value*.

**Table 3:** **MDC protein members of rice**.

**Gene**	**Proteins**	**Locus**	**Coordinate 5′–3′**	**AA**	**Subcellular localization**	**MW (Da)**	**pI**
**ONE MATH DOMAIN**							
*OsM1*	OsM1a	LOC_Os01g56800.1	32,784,325–32,773,565	1278	NUC	141,414	7.0917
	OsM1b	LOC_Os01g56800.2	32,783,710–32,773,479	1253	NUC	138,487	6.8639
	OsM1c	LOC_Os01g56800.3	32,783,710–32,773,565	1250	NUC	138,199	6.8639
*OsM2*	OsM2a	LOC_Os01g56490.1	32,569,756–32,552,611	1111	NUC	129,097	5.4099
*OsM3*	OsM3a	LOC_Os04g18830.1	10,474,689–10,475,396	236	NUC	25,518.8	8.2291
*OsM4*	OsM4a	LOC_Os05g43280.1	25,176,651–25,186,745	1262	NUC	139,463	6.5865
*OsM5*	OsM5a	LOC_Os07g06950.1	3,411,719–3,424,507	999	CYT	117,172	6.0369
*OsM6*	OsM6a	LOC_Os07g20130.1	11,627,408–11,628,385	223	CHL	24,567.2	8.211
*OsM7*	OsM7a	LOC_Os10g28130.1	14,607,689–14,606,812	214	CYT	22,946.7	4.7678
*OsM8*	OsM8a	LOC_Os11g41360.1	24,812,851–24,813,612	224	CHL	25,088.8	9.1168
*OsM9*	OsM9a	LOC_Os11g27030.1	15,564,541–15,565,050	170	CYT	19,223.3	8.0773
*OsM10*	OsM10a	LOC_Os11g41230.1	24,724,484–24,725,050	189	MIT	20,316.4	6.6431
*OsM11*	OsM11a	LOC_Os11g41240.1	24,727,308–24,727,862	185	CHL	20,177.4	7.1494
*OsM12*	OsM12a	LOC_Os12g40520.1	25,069,598–25,077,639	1138	NUC	126,956	6.3151
*OsM13*	OsM13a	LOC_Os12g30540.1	18,334,665–18,349,360	1126	NUC	131,937	5.6182
**ONE MATH** + **ONE BTB DOMAIN**							
*OsMB1*	OsMB1	LOC_Os02g20690.1	12,192,602–12,191,631	324	NUC	36,457.5	7.5467
*OsMB2*	OsMB2	LOC_Os02g20620.1	12,154,630–12,153,845	262	NUC	29,073.4	8.1126
*OsMB3*	OsMB3	LOC_Os02g20720.1	12,218,391–12,219,563	391	CYT	43,593.1	4.7974
*OsMB4*	OsMB4	LOC_Os02g20590.1	12,144,157–12,143,096	354	CHL	39,558.4	7.1767
*OsMB5*	OsMB5a	LOC_Os03g57854.1	32,957,898–32,964,626	432	NUC	46,983.5	5.4251
	OsMB5b	LOC_Os03g57854.2	32,957,898–32,964,626	379	NUC	41,390.4	5.3836
*OsMB6*	OsMB6	LOC_Os04g53410.1	31,812,018–31,813,118	367	CYT	40,472.2	6.562
*OsMB7*	OsMB7	LOC_Os04g35310.1	21,474,453–21,472,975	369	CHL	40,695.4	6.8411
*OsMB8*	OsMB8	LOC_Os06g45730.1	27,685,556–27,683,619	365	CHL	39,446	6.5097
*OsMB9*	OsMB9	LOC_Os07g01140.1	85,934–82,397	396	CHL	43,753.6	6.6421
*OsMB10*	OsMB10a	LOC_Os07g07270.1	3,614,403–3,610,786	425	CYT	46,159.7	5.1953
	OsMB10b	LOC_Os07g07270.2	3,614,403–3,610,786	372	CYT	40,474.5	5.1341
*OsMB11*	OsMB11	LOC_Os07g46160.1	27,545,275–27,550,563	435	CHL	47,093.8	6.7769
*OsMB12*	OsMB12	LOC_Os08g31430.1	19,442,644–19,441,238	402	CHL	44,016.8	5.4363
*OsMB13*	OsMB13	LOC_Os08g12960.1	7,694,865–7,693,822	307	CYT	34,089.8	5.2765
*OsMB14*	OsMB14	LOC_Os08g31450.1	19,452,059–19,451,229	277	CYT	30,753.6	6.0999
*OsMB15*	OsMB15	LOC_Os08g13180.1	7,834,796–7,835,950	385	CHL	42,640.7	6.509
*OsMB16*	OsMB16	LOC_Os08g13030.1	7,740,373–7,741,464	364	CHL	40,755.3	5.0818
*OsMB17*	OsMB17	LOC_Os08g03490.1	1,634,503–1,635,537	345	CHL	38,044.7	9.4141
*OsMB18*	OsMB18	LOC_Os08g03470.1	1,628,504–1,631,173	371	CHL	41,778.9	5.0812
*OsMB19*	OsMB19	LOC_Os08g13000.1	7,718,114–7,719,211	366	CYT	40,473.1	4.974
*OsMB20*	OsMB20a	LOC_Os10g29180.1	15,199,437–15,202,164	376	CYT	41,732.3	5.2703
	OsMB20b	LOC_Os10g29180.2	15,199,437–15,200,849	370	CHL	40,906.3	5.1365
*OsMB21*	OsMB21	LOC_Os10g29230.1	15,218,256–15,219,365	370	CHL	40,959.7	5.1501
*OsMB22*	OsMB22	LOC_Os10g29310.1	15,245,005–15,246,475	364	MIT	40,272.1	6.4353
*OsMB23*	OsMB23	LOC_Os10g29220.1	15,213,679–15,215,127	357	CHL	39,791.7	7.8764
*OsMB24*	OsMB24	LOC_Os10g29050.1	15,138,447–15,140,147	363	CHL	40,324.2	7.1538
*OsMB25*	OsMB25	LOC_Os10g29100.1	15,167,886–15,166,777	370	CHL	40,985.6	6.3166
*OsMB26*	OsMB26	LOC_Os10g29020.1	15,124,391–15,125,458	313	CHL	34,483.3	6.3492
*OsMB27*	OsMB27	LOC_Os10g29330.1	15,255,885–15,257,174	360	CHL	39,869.1	4.7875
*OsMB28*	OsMB28	LOC_Os10g28860.1	15,044,042–15,045,403	373	CYT	40,566.9	5.7543
*OsMB29*	OsMB29	LOC_Os10g29110.1	15,170,899–15,169,211	410	CHL	44,731	8.0775
*OsMB30*	OsMB30	LOC_Os10g29380.1	15,268,591–15,269,703	371	CYT	41,008.4	4.6732
*OsMB31*	OsMB31	LOC_Os10g29150.1	15,183,967–15,182,652	391	CHL	43,209.9	5.6054
*OsMB32*	OsMB32	LOC_Os10g28990.1	15,110,429–15,111,589	387	CHL	43,499.1	7.2487
*OsMB33*	OsMB33	LOC_Os10g28790.1	15,015,529–15,016,650	374	CYT	40,251.5	7.1539
*OsMB34*	OsMB34	LOC_Os10g29750.1	15,468,056–15,466,956	367	CHL	40,763.5	6.3456
*OsMB35*	OsMB35	LOC_Os10g29290.1	15,239,701–15,240,792	364	CHL	40,122.8	6.2353
*OsMB36*	OsMB36	LOC_Os10g30360.1	15,774,570–15,775,465	254	NUC	28,742.1	5.8616
*OsMB37*	OsMB37	LOC_Os10g29840.1	15,506,569–15,507,648	360	CHL	40,329.2	6.6647
*OsMB38*	OsMB38	LOC_Os10g29740.1	15,462,806–15,463,918	371	CHL	40,929.5	6.5608
*OsMB39*	OsMB39	LOC_Os10g29790.1	15,486,180–15,487,367	396	CYT	43,715.9	4.7729
*OsMB40*	OsMB40	LOC_Os10g28760.1	15,003,427–15,004,575	383	CYT	42,366.3	6.5094
*OsMB41*	OsMB41	LOC_Os10g28780.1	15,012,324–15,010,666	384	CYT	42,247	6.3774
*OsMB42*	OsMB42	LOC_Os10g29410.1	15,281,164–15,279,959	402	CYT	43,832.4	5.8263
*OsMB43*	OsMB43	LOC_Os10g29950.1	15,547,685–15,546,516	350	CYT	38,533	4.6588
*OsMB44*	OsMB44	LOC_Os10g29850.1	15,519,165–15,514,718	356	CHL	39,671.2	7.0526
*OsMB45*	OsMB45	LOC_Os10g29810.1	15,499,315–15,498,122	398	CHL	43,691.5	5.357
*OsMB46*	OsMB46	LOC_Os10g29495.1	15,324,463–15,331,200	719	CHL	79,001.7	7.2184
*OsMB47*	OsMB47	LOC_Os10g29340.1	15,259,550–15,260,554	306	CHL	34,092.3	9.3761
*OsMB48*	OsMB48	LOC_Os10g28770.1	15,007,280–15,006,162	373	CYT	40,976.7	6.5747
*OsMB49*	OsMB49	LOC_Os10g29120.1	15,171,570–15,173,056	323	CHL	35,436.5	7.1534
*OsMB50*	OsMB50	LOC_Os11g41310.1	24,758,712–24,759,854	381	CHL	40,767.1	8.7039
*OsMB51*	OsMB51	LOC_Os11g41350.1	24,810,818–24,809,640	393	CYT	42,053.4	4.8638
*OsMB52*	OsMB52	LOC_Os11g40680.1	24,280,165–24,278,622	371	CYT	40,235.5	5.659
*OsMB53*	OsMB53	LOC_Os11g40220.1	23,994,340–23,993,291	343	CHL	37,346.5	7.5005
*OsMB54*	OsMB54	LOC_Os11g45560.1	27,579,282–27,576,676	371	CHL	39,994.9	9.9389
**TWO MATH DOMAIN**							
*Os2M1*	Os2M1	LOC_Os10g33830.1	17,956,926–17,945,282	686	VAC	78,376.8	9.6134
**TWO MATH** + **TWO BTB DOMAIN**							
*Os2M2B1*	Os2M2B1	LOC_Os11g41260.1	24,734,244–24,737,370	655	CYT	71,398.8	5.7745

*The genes and their respective proteins have been prefixed by “OS” The alternative spliced forms have been postfixed with the alphabets like “a,” “b” and so on. The table shows their predicted sub-cellular localization like (CHL), Chloroplast; (CYT), Cytoplasm; (NUC), Nucleus; (VAC), Vacuole; (MIT), Mitochondria; (EXT), Extracellular; (CYS), Cytoskeleton; or (PER), Peroxisome; along with their (MW), molecular weight in (Da), Dalton and pI value*.

### Phylogenetic analysis of MDC proteins

**Figure 1:**
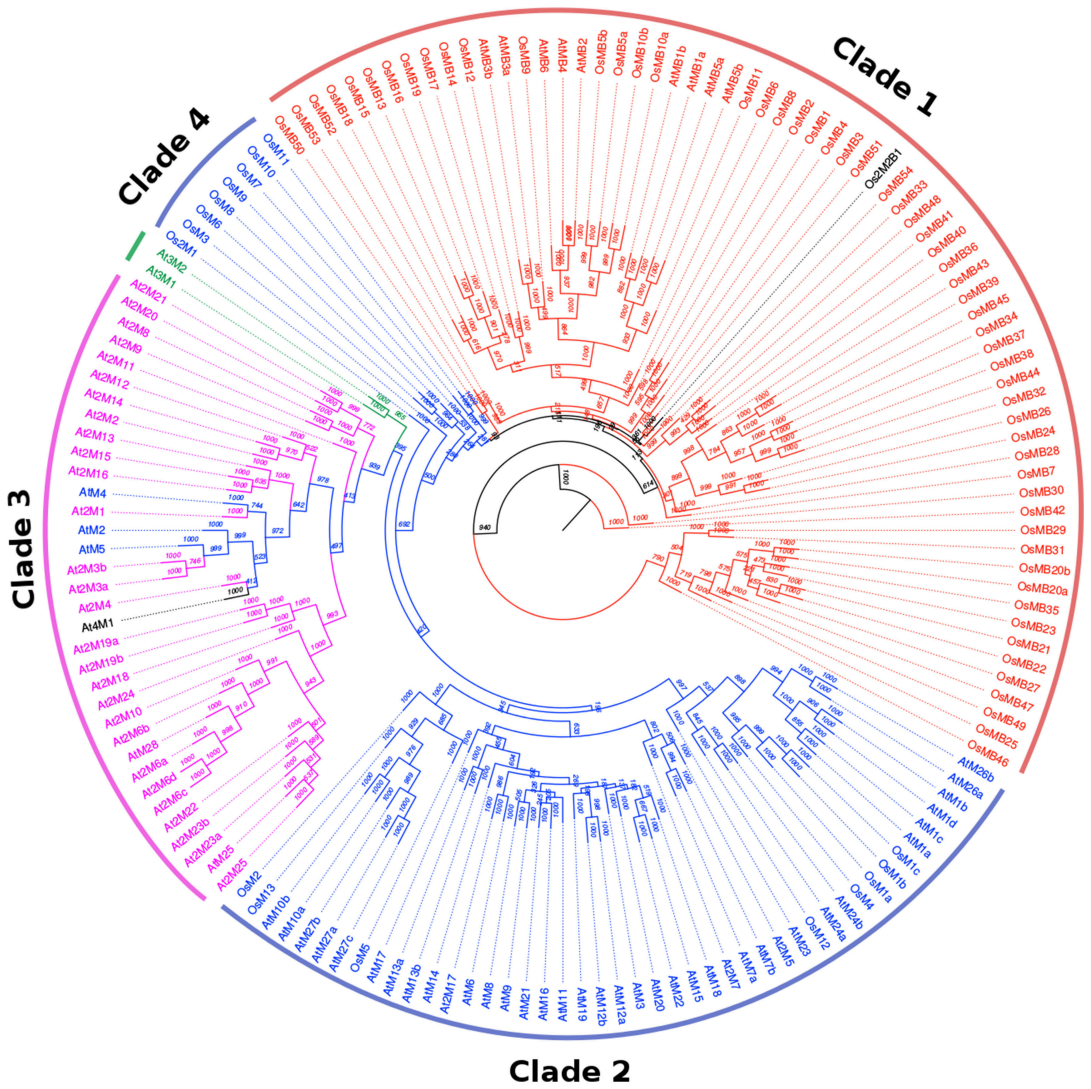
**Rooted phylogenetic relationship tree of the MDC protein members of both Arabidopsis and rice**. The rooted tree shows the presence of MDC proteins having single MATH domain (blue), MATH domain along with BTB domain (Red), two MATH domains (pink), three MATH domains (green), four MATH domains (black), and two MATH and two BTB domains (black). The bootstrap values are marked on the rooted tree.

To analyze the phylogenetic relationship between the MDC proteins in both Arabidopsis and rice, a rooted tree was prepared by aligning full-length protein sequence (Figure 1). This analysis gave a comprehensive picture of MDC protein classification. The single MATH domain containing proteins (AtM and OsM) were observed to be clustered in two groups (Clade 2 and 4) and the other major clade (Clade 1) was of proteins having one MATH domain along with one BTB domain (AtMB and OsMB). The third major clade was of the proteins having two MATH domains (At2M and Os2M). The three MATH domain containing proteins (At3M) formed a separate cluster with the group of proteins having two MATH domain containing proteins. Further, proteins with two MATH domains and two BTB domains of rice were found in the same clade as those of protein sequences with one MATH and one BTB domain. In addition, single-domain MDC proteins from Arabidopsis (AtM2, AtM4, AtM5, and At4M1) were found in the same clade as two MATH domain containing proteins from Arabidopsis. Similarly, proteins containing two MATH domains in Arabidopsis (At2M17, At2M7, and At2M5) were found to be present in the clade belonging to MDC proteins containing one domain.

### Sequence analysis of MDC proteins

Amino acid sequence analysis of the MDC proteins revealed that single MATH domain containing protein OsM7 shared a very low level of identity with other single MATH domain proteins in rice (ranging from 19 to 28%) while it was found to be closer to the proteins with single MATH and single BTB domain (28–32% identity). Interestingly, all single MATH domain containing proteins from Arabidopsis showed significant identity (30–77%) with other members of their group, except for a few single MATH domain proteins from rice such as, OsM3, OsM6, OsM8, OsM9, OsM10, and OsM11, which showed only 15–22% identity (Figure S2). This was also evident from the phylogenetic tree where these protein sequences were found to lie in the separate clade from other single MATH domain containing protein sequences. The amino acid sequences of MDCPs containing single MATH domain along with single BTB domain (OsMB) were found to have 27–77% identity within their group. The two MATH domain containing proteins were found to be sharing 26–41% identity within their group. The single MATH domain containing protein AtM5 was found to possess 25–60% identity with the two MATH domain containing members. Similarly, AtM28, AtM2, and AtM4 shared 28–62% identity with the members having two MATH domains (Figure S3). The protein with two MATH domains along with two BTB domains in rice (Os2M2B1) was observed to be having 34–56% homology with the protein sequences having one MATH and one BTB domain. The two MATH domain containing proteins were observed to have 26–46% identity within their group (Figure S4). The four MATH domain containing protein in Arabidopsis, At4M1 was found to be sharing 28–51% identity with the members having two MATH domains. Analysis of alignment of all the MATH domain protein sequences suggests large-scale insertion in various protein sequences leading to low sequence identity between the sequences.

### Chromosomal localization of MDC protein encoding genes

**Figure 2:**
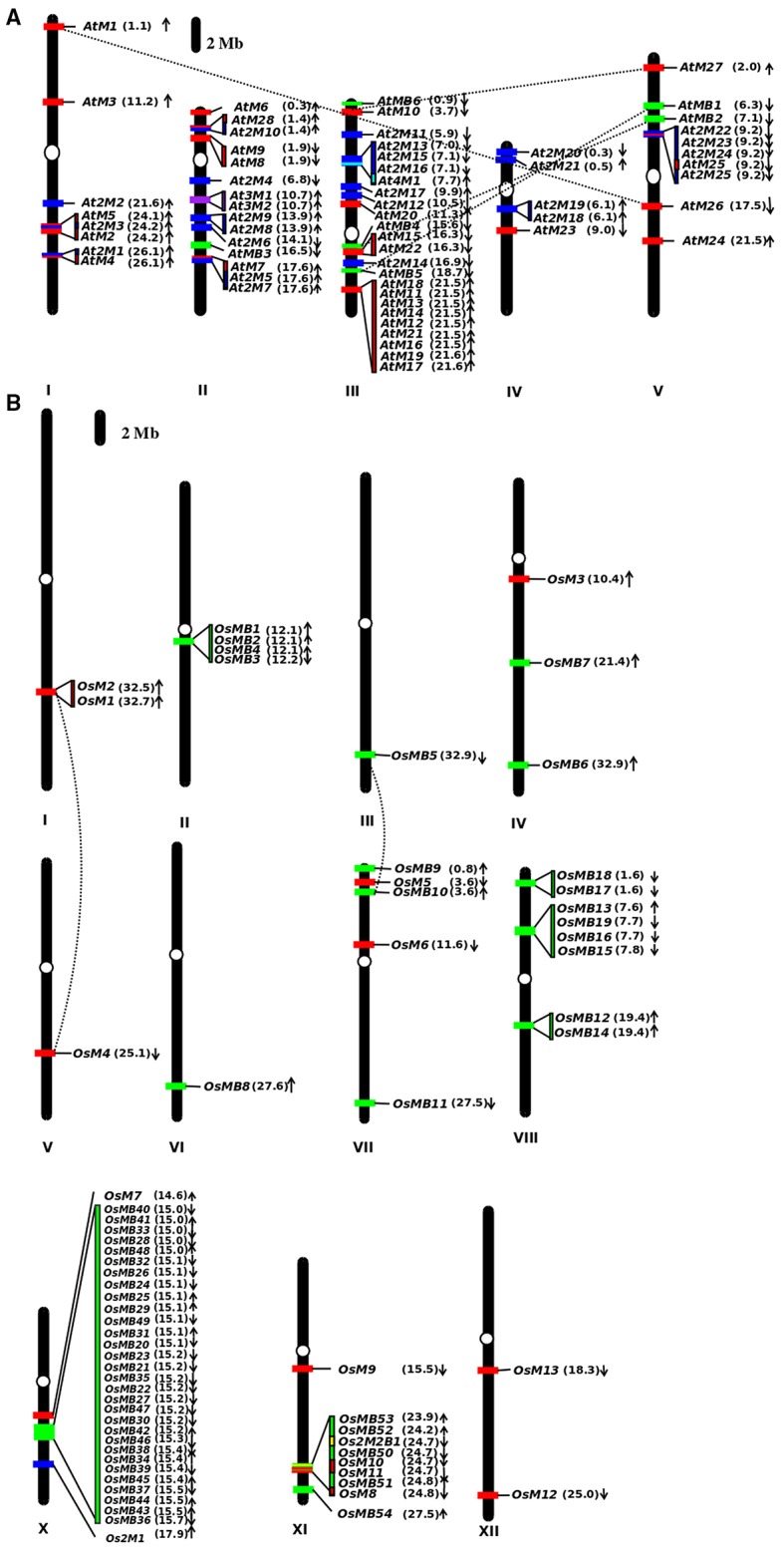
**Graphical scaled representation of the location of MDC protein encoding genes on the chromosomes of (A) Arabidopsis and (B) rice**. The centromeres are marked by ovals on the chromosomes. The position of MDC protein encoding genes has been marked in Mb in the parenthesis along with the direction of the ORF. The figure shows the MDC proteins having single MATH domain (red), MATH domain along with BTB domain (green), two MATH domains (blue), three MATH domains (purple), four MATH domains (cyan), and two MATH and two BTB domains (yellow).

The analysis of the localization of MDC protein encoding genes on the chromosomes of Arabidopsis and rice reveals an interesting pattern. In Arabidopsis, the majority (28) of single MATH domain containing protein encoding genes were found to be localized uniformly on all the chromosomes (Figure 2A). Interestingly, maximum i.e., thirteen number of MDC proteins encoding genes were found to be present on chromosome III in Arabidopsis. Out of these, nine were forming a cluster. Further, five single domain MDC protein encoding genes namely, *AtM1, AtM2, AtM3, AtM4*, and *AtM5* were located on chromosome I. The chromosome II and V were observed to contain four single domain MDC protein encoding genes. In Arabidopsis, 4 genes encoding MDC proteins were duplicated in the genome. The single domain MDC protein coding gene, *AtM1*, present on chromosome I was found to be duplicated on chromosome V as single domain MDC protein encoding gene *AtM26*. Another gene, *AtM10* from chromosome III was found to be duplicated as *AtM27* on chromosome V. Among the group of MDC proteins having BTB domain, *AtMB4* present on chromosome III was found to be duplicated as *AtMB2* on chromosome V. Another gene of the same group *AtMB1* from chromosome V was found to be duplicated with *AtMB5* on chromosome III (Figure 2A).

In rice, genes coding for one domain MDC were found to be scattered on various chromosomes (Figure 2B). It was found that out of 13 single domain containing genes, chromosome XI contained 4 genes (i.e., *OsM8, OsM9, OsM10*, and *OsM11*), chromosome I, VII, and XII contained 2 genes each, while chromosome IV, V, and IX contained only one single MATH domain coding gene. However, chromosome II, III, VI, and VIII did not contain any single MATH domain protein encoding gene. Surprisingly, in rice chromosome IX does not contain any MDC protein coding gene. Analysis of segmental duplications in MDC proteins revealed only two events of gene duplication in rice. The first instance where single-domain MDC protein encoding gene *OsM1* present on chromosome I was found to be duplicated as *OsM4* present on chromosome V. The other duplicated gene was MDC protein with a BTB domain, *OsMB5* present on chromosome III was found duplicated on chromosome VII as *OsMB10*.

In rice, genes coding for proteins having single MATH domain along with single BTB domain were found in large numbers (54) unlike *Arabidopsis* (6). In *Arabidopsis*, genes that belong to this group were found on chromosome II, III, and V. Interestingly, the maximum number (3) of genes are present on the chromosome III namely, *AtMB4, AtMB5*, and AtMB6 followed by two genes present on chromosome V namely, *AtMB1* and *AtMB2* (Figure 2A). In rice, striking observation was noticed with respect to these genes where most of the genes of the group (30) are present on the chromosome X in a cluster within the same region. Further, eight genes of the group were found on chromosome VIII followed by five on chromosome XI, three on chromosome VII and two on chromosome IV. Chromosome III and VI contains only single gene each belonging to this group only. The chromosome II was found to have genes (four in number) from the group in a small cluster (Figure 2B).

The genes encoding proteins having two MATH domains in Arabidopsis (25) are found to be distributed between all chromosomes while in rice, only one gene from this group is located on chromosome X. Further, their distribution on the chromosome in Arabidopsis also presents an interesting pattern. A large number of such genes (total seven in number) were found to be present on chromosome II and III and further four genes were present on chromosome IV and V while chromosome I was observed to have three genes encoding for proteins having two MATH domains.

With only single instance of a protein having two MATH domains along with two BTB domains (2M2B) in rice, the gene was found to be present on chromosome XI while none of the protein of this group was present in Arabidopsis. However, in Arabidopsis, two proteins having three MATH domains were found and the genes encoding both these proteins were located together on chromosome II. Further, only one protein that too in Arabidopsis, having four MATH domains was observed. The gene encoding this protein was found to be localized on chromosome III.

### Sub-cellular localization of MDC proteins

**Figure 3:**
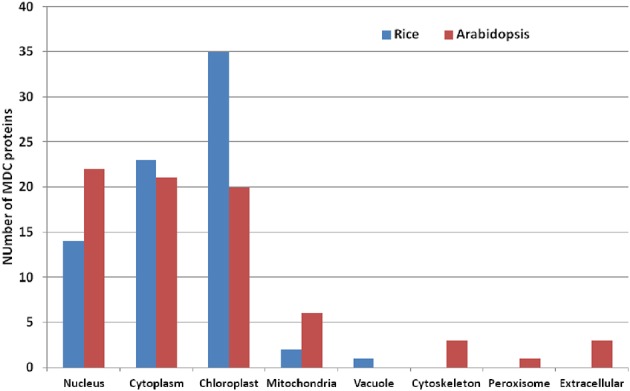
**Bar diagram showing predicted sub-cellular localization of the members of MDC proteins in Arabidopsis and rice**.

Analysis of the sub-cellular localization of MDC proteins in *Arabidopsis* and rice presented an interesting pattern (Figure 3). Twenty-two MDC proteins in *Arabidopsis* were predicted to be localized in the nucleus, 21 in the cytoplasm and 20 in the chloroplast (Table 2). In contrast, majority of the rice MDC proteins were predicted to be present in either the chloroplast (35) or in the cytoplasm (23) (Table 3). Further analysis in rice revealed that mostly single MDC proteins were predicted to be localized in the nucleus. However, proteins containing MATH domain along with the BTB domain were predicted to be localized in the cytoplasm and the chloroplast. In *Arabidopsis*, six MDC proteins were predicted to be localized in the mitochondria in comparison to two in rice. The MDC proteins in Arabidopsis were also predicted to be localized in other sub-cellular locations such as, cytoskeleton, peroxisome, and extracellular matrix. These were mainly one and two MATH domain containing proteins. Similarly, two MATH domain protein of rice (Os2M1) was specifically predicted to be localized in the vacuole.

### Expression analysis of MDC protein encoding genes

#### In various tissues

**Figure 4:**
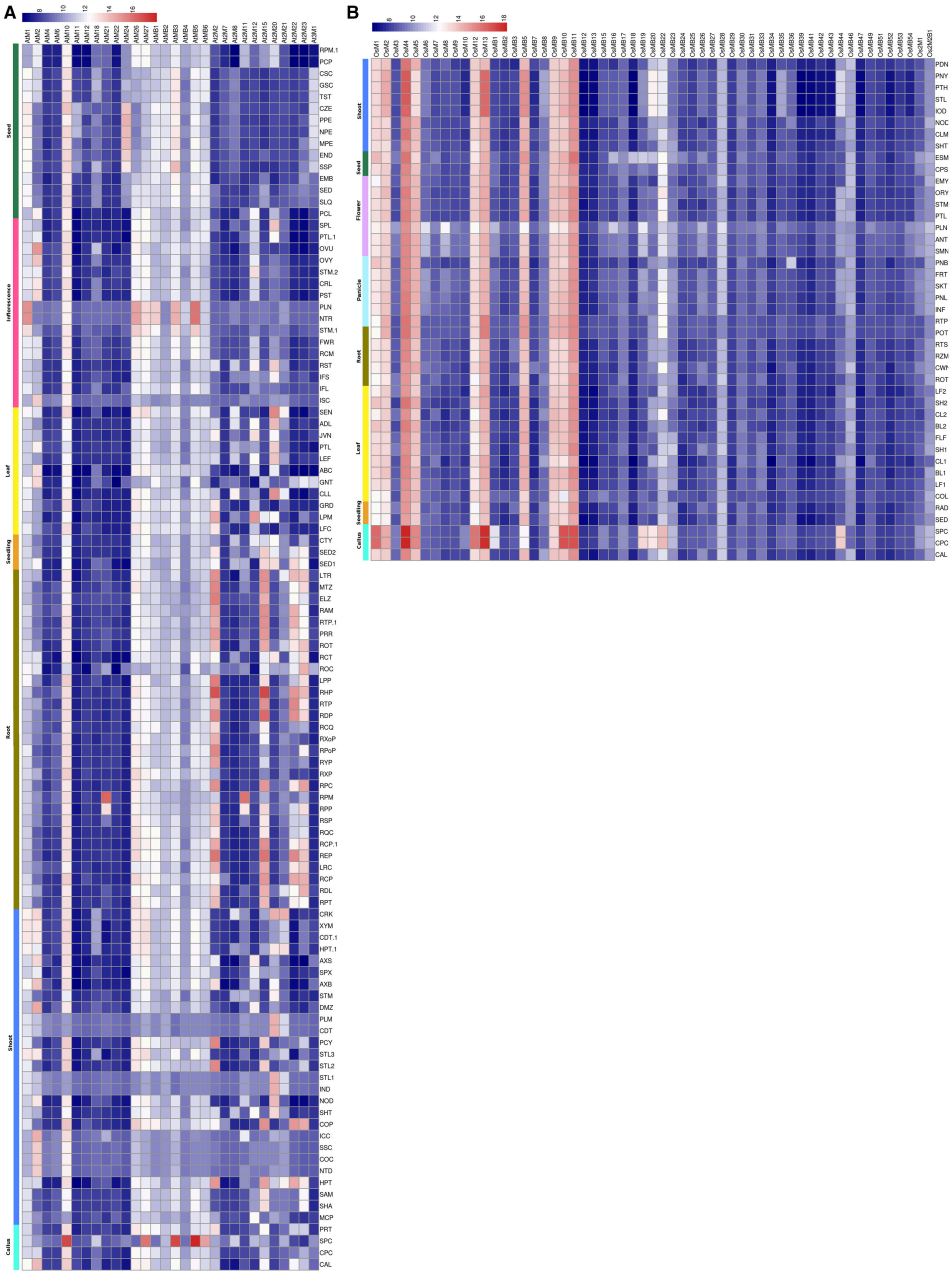
**Heatmap representation of the expression of MDC protein encoding genes in various tissues in Arabidopsis (A) and rice (B)**. The expression values were obtained from Affymetrix array databases using Genevestigator Response Viewer (https://www.genevestigator.com). For Arabidopsis, 22 K ATH1 genome array was chosen along with pre-existing microarray and in case of rice, microarray results of OS_51 K: Rice Genome 51 K pre-existing microarrays were chosen. The details of the libraries used in the current analysis are presented in Table S1.

The expression analysis of MDCP encoding genes in Arabidopsis using 22 K ATH1 genome array dataset showed that most of the MDCPs encoding genes showed transcript at low levels in various tissues (Figure 4A, Table S1). The genes coding for BTB domain containing MDCPs showed low or no expression in Arabidopsis except *AtMB1, AtMB3*, and *AtMB5* which showed increased expression in the inflorescence. Even in rice such genes showed similar levels of expression, except for *OsMB5, OsMB9, OsMB10*, and *OsMB11* which were up-regulated in various tissues (Figure 4B, Table S1). Expression analysis in calli showed increased levels of *AtMB1, AtMB3*, and *AtMB5* from Arabidopsis and *OsMB9, OsMB10*, and *OsMB11* from rice. In Arabidopsis, single-domain MDCP encoding genes showed low expression in various tissues, except for *AtM1* and *AtM2* which were found to be up-regulated in the inflorescence. Another single-domain MDCP encoding gene *AtM10* was found to be up-regulated in callus but also maintained a minimum level of expression across various tissues. In rice, six of the single-domain MDCP encoding genes viz. *OsM1, OsM2, OsM4, OsM5, OsM12*, and *OsM13* were found to be highly up-regulated in various tissues. The two MDCP encoding genes *At2M2* and *At2M15* in Arabidopsis showed high expression in roots. Further, *At2M23* showed variability in expression in roots but remained at low levels in other tissues. This analysis indicated that in rice at least 10 MDCP encoding genes were highly expressed throughout all tissues suggesting their possible role in the combinatorial transcriptional regulation of a broad set of genes in various tissues.

#### At various developmental stages

**Figure 5:**
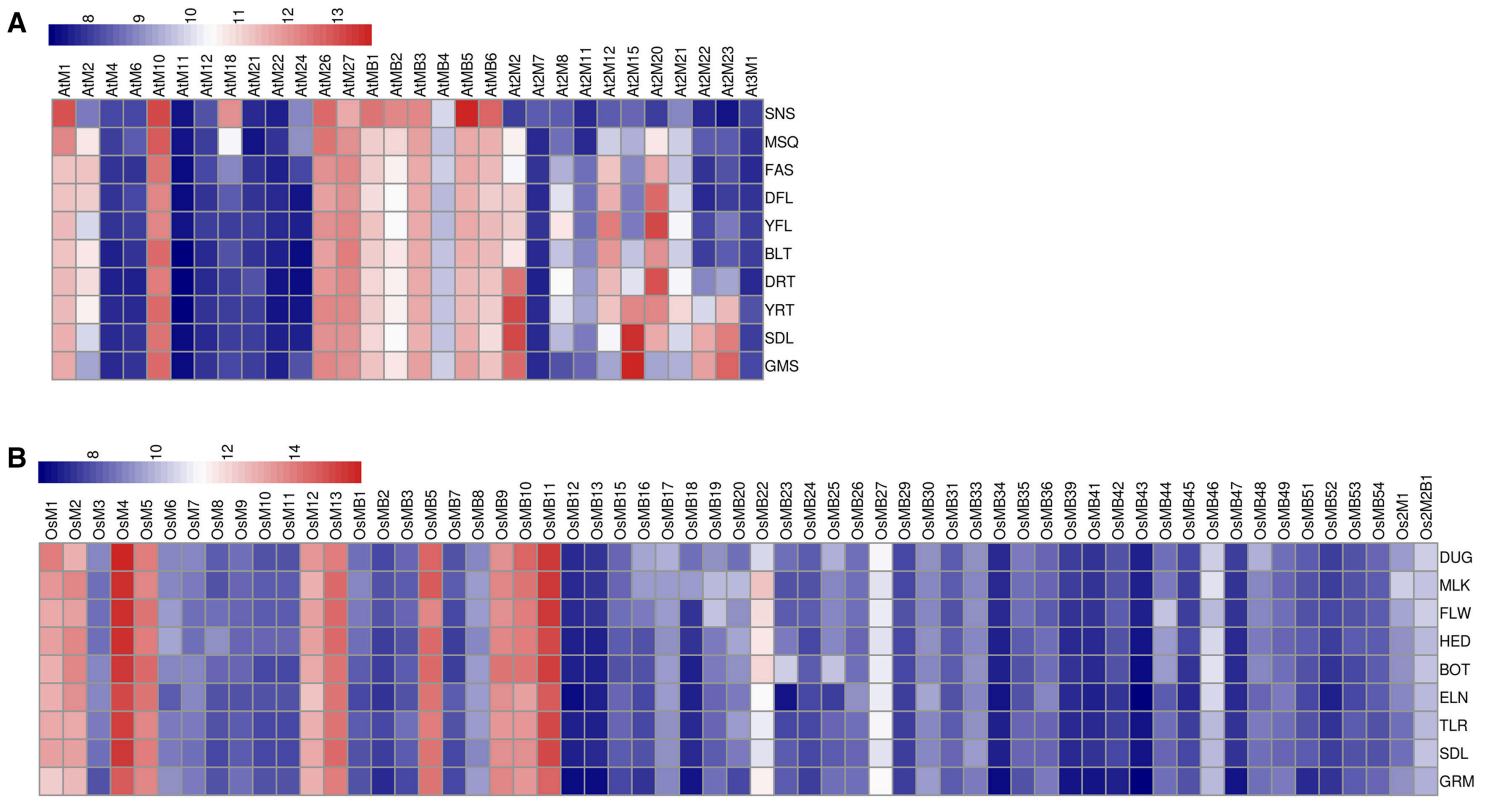
**Heatmap representation of the expression of MDC protein encoding genes at various developmental stages in (A) Arabidopsis and (B) rice**. The expression values were obtained from Affymetrix array databases using Genevestigator Response Viewer (https://www.genevestigator.com). For Arabidopsis, 22 K ATH1 genome array was chosen along with pre-existing microarray and in case of rice, microarray results of OS_51 K: Rice Genome 51 K pre-existing microarrays were chosen. The conditions considered for analysis in Arabidopsis are: (SNS), senescence; (MSQ), mature siliques;(FAS), flowers and siliques; (DFL), developed flower; (YFL), young flower;(BLT), bolting;(DRT), developed rosette; (YRT), young rosette; (SDL), seedling; (GMS), germinated seeds. The conditions considered for analysis in rice are: (DUG), dough stage; (MLK), milk stage; (FLW), flowering stage; (HED), heading stage; (BOT), booting stage; (ELN), stem elongation stage; (TLR), tillering stage;(SDL), seedling; (GRM), germination. The details of the libraries used in the current analysis are presented in Table S2.

To check the transcript levels of MDCPs encoding genes at various developmental stages of Arabidopsis and rice, publicly available microarray data was analyzed. In Arabidopsis, single-domain MDCPs encoding genes *AtM1, AtM10, AtM26*, and *AtM27* were found to be up-regulated during all the developmental stages while *AtM18* showed higher expression only during the senescence stage (Figure 5A, Table S2). Further, *AtM2* showed variable expression during various developmental stages except for senescence and germinating seed stage where its levels remained low. In rice, the single-domain *OsM4* showed significantly high expression at different developmental stages. The *OsM1, OsM2, OsM5, OsM12*, and *OsM13* also showed high expression throughout all the developmental stages (Figure 5B, Table S2). The genes encoding MDCPs with BTB domain showed comparatively higher expression during all the developmental stages in Arabidopsis. While in rice, only four genes viz. *OsMB5, OsMB9, OsMB10*, and *OsMB11* showed high expression during all the developmental stages. Rest of the other similar genes showed relatively lower expression in all the developing tissues in rice except for *OsMB22* gene which showed variable expression. The two domain MDCPs coding genes in Arabidopsis showed differential expression in various tissues. The *At2M2* gene showed comparatively high expression in the young rosette and seedling stage of the plant while maintaining variable expression in other tissues. Similarly, *At2M15* gene showed higher expression during seed germination and seedling stage, while maintaining lower levels in most of the other developing tissues. The *At2M20* showed minimal to high expression in all the developing tissues except for senescence and germinating seeds. The genes encoding two MATH domain MDC proteins in rice (*Os2M1* and *Os2M2B1*) were observed to be expressed at lower levels in all the developmental tissues.

#### In response to various abiotic stress conditions

In Arabidopsis, most of the MDCP-coding genes maintain minimal expression under various abiotic stress conditions, while in rice the expression of MDC protein coding genes gets down-regulated (Figure S5A,B, Table S3). Interestingly, gene encoding two domain MDC protein *At2M2*, was found to be up-regulated in both root and shoot tissues during the late phase of both salinity and osmotic stress. Another gene *At2M12* showed high expression under drought stress condition in both early and late phase in shoots. Similarly, *At2M23* showed higher expression in shoots during the late phase of wounding. On the other hand in rice, gene encoding MATH-BTB domain containing proteins i.e., *OsMB10* and *OsMB11* showed high expression under salinity as well as drought stress. However, slight up-regulation was observed for *OsMB12* and *OsMB5* under salinity and drought stress and for *OsMB19, OsMB20, OsMB22*, and *OsMB46* under heat stress. Interestingly, all the MATH domain encoding genes showed down-regulation under cold stress.

#### In response to various biotic stress conditions

**Figure 6:**
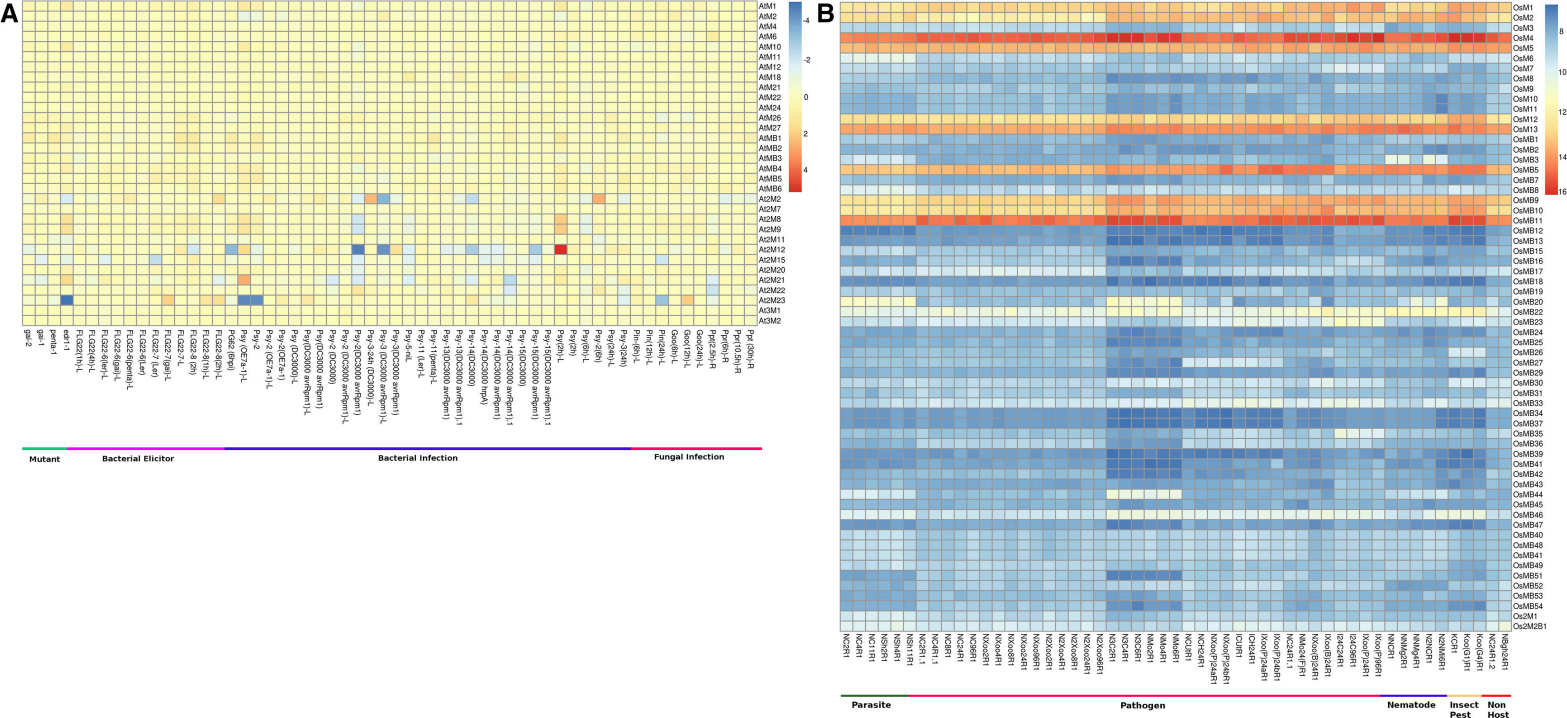
**Heatmap representation of the expression of MDC protein encoding genes in response to various biotic stresses in (A) Arabidopsis and (B) rice**. The expression values were obtained from Affymetrix array databases using Genevestigator Response Viewer (https://www.genevestigator.com). For Arabidopsis, 22 K ATH1 genome array was chosen along with pre-existing microarray and in case of rice, microarray results of OS_51 K: Rice Genome 51 K pre-existing microarrays were chosen. The details of the libraries used in the current analysis are presented in Table S4.

Under the biotic stresses, all the genes encoding single MDC proteins and also genes coding for proteins containing MATH with BTB domain showed very low expression in Arabidopsis (Figure 6A, Table S4). However, only genes coding for two MATH domain containing proteins showed differential expression under biotic stresses. On the other hand, MDCP encoding genes in rice showed an interesting pattern of expression. Single domain MDC protein encoding genes such as *OsM1, OsM2, OsM4, OsM5, OsM12*, and *OsM13* showed significant up-regulation in response to various biotic stress conditions studied here (Figure 6B, Table S4). The genes coding for MDC proteins having BTB domain such as OsMB9, OsMB10, and OsMB11 also showed high up-regulation under various biotic stress conditions. All the other MDC genes showed little response toward the biotic stresses.

#### qRT-PCR based expression analysis of MDCP coding genes under abiotic stresses

**Figure 7:**
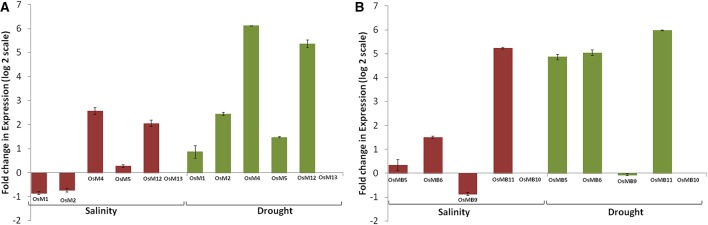
**qRT-PCR confirms altered expression of selected biotic stress responsive genes under abiotic stress conditions**. Bar diagram depicting fold change (log_2_ scale) in expression of selected single MATH domain containing genes **(A)** and single MATH along with single BTB domain containing genes **(B)** under salinity and drought stress conditions based on qRT-PCR analysis. For this analysis, 10 day old seedlings of IR64 variety (a moderately sensitive cultivar) of rice were subjected to stress treatments for 6 h followed by RNA isolation, first strand cDNA synthesis and real-time PCR. Error bars show standard deviation.

Expression analysis of large gene family members through the publicly available database and validation of selected gene expression pattern using qRT-PCR, is a useful approach, which provides primary information about the newly identified gene function (Singh et al., 2013). However, in few incidences, data retrieved through different resources may vary. Thus, to confirm the expression profile of MDCP encoding genes, we chose 11 representative OsMDCP encoding genes which were reported to be highly up-regulated in different tissues (Figure 4B), at different developmental stages (Figure 5B) as well as under different biotic stresses (Figure 6B). The level of expression of these selected 11 genes was further checked under abiotic stress conditions such as salinity (200 mM NaCl) and drought (air dry) to study their cross-inducibility. Our qRT-PCR results under these stresses corroborated with the expression pattern obtained by publicly available microarray data (Figure S5B). For instance, *OsM4, OsM5*, and *OsM12* expression was up-regulated after 6 h of salt and drought stress, while *OsM1* and *OsM2* were up-regulated under drought stress only (Figure 7A). Similarly, an up-regulation in *OsMB5, OsMB6* and *OsMB11* levels and down-regulation in *OsMB9* levels was observed under both salinity and drought stress (Figure 7B). The levels of *OsM13* and *OsMB10* could not detected in the qRT-PCR analysis. Our qRT-PCR results for *OsM2, OsM12, OsMB5, OsMB9*, and *OsMB11* under salinity stress and *OsM12, OsMB5*, and *OsMB9* under drought effectively validate the expression profile obtained from the publicly available database, thereby providing more authentic expression picture of MDCP family members. However, the transcript profile of *OsM1, OsM4, OsM5, OsMB6, OsM13*, and *OsMB13* under salinity stress, and *OsM1, OsM2, OsM4, OsM5, OsM13, OsMB6*, and *OsMB10* under drought stress did not corroborate well with their respective microarray data. These differences in expression levels in the publicly available microarray and qRT-PCR may be either due to genotypic differences between the samples or due to differences in the plant developmental stages.

In addition, when we compare our qRT-PCR data with the biotic stress data from the publicly available database we found that most of the salt stress-responsive MDCP encoding genes namely, *OsM4, OsM5, OsM12, OsMB5, OsMB6*, and *OsMB11* showed a positively correlated response to biotic stress. Similarly, most of the drought stress-responsive MDCP encoding genes namely, *OsM1, OsM2, OsM4, OsM5, OsM12, OsMB5, OsMB6*, and *OsMB11* showed a positively correlated response to biotic stress. This indicates toward a significant role of these genes in both abiotic and biotic stress response. However, certain genes showed an inverse correlation between biotic and abiotic stress response. These genes are *OsM1, OsM2, OsM13, OsMB9*, and *OsMB10* under salinity stress while *OsM13, OsMB9*, and *OsMB10* under drought stress. Importantly, most of the genes i.e., *OsM4, OsM5, OsM12, OsMB5, OsMB6*, and *OsMB11* showed positive correlation under all biotic and abiotic stress conditions, while *OsM13, OsMB9* and *OsMB10* showed an inverse correlation among biotic and abiotic stress response.

## Discussion

Using subtractive hybridization approach in two contrasting cultivars of rice, Pokkali (salt tolerant) and IR64 (salt sensitive) 1194 high-throughput ESTs (584 from IR64 and 610 from Pokkali) were obtained in our previous study (Kumari et al., 2009). These ESTs were believed to be playing a significant role in salt stress tolerance in rice at the seedling stage. The MDC proteins were identified through this study as potential candidates that may play a role in both abiotic and biotic stress response. Earlier, the MDC proteins have been reported and analyzed for their role in plant-microbe interaction (Oelmüller et al., 2005). The analysis suggested that the MATH domain containing protein located at the plasma membrane in roots of Arabidopsis perceives the first signal for the presence of basidiomycete *Piriformospora indica* (Shahollari et al., 2005). In the present analysis, we have identified and classified the MATH domain containing proteins in Arabidopsis and rice and further, analyzed their potential role in the abiotic stress response. We have identified a total of 156 MDC proteins, with 62 genes encoding 82 MDC proteins in Arabidopsis and 69 genes encoding 74 MDC proteins in rice in comparison to an earlier report by Oelmüller et al. (2005), which identified 59 genes in Arabidopsis. Another previous study has reported the presence of 6 MATH-BTB genes in Arabidopsis and 69 MATH-BTB genes in rice while analyzing BTB superfamily in grasses (Juranić and Dresselhaus, 2014). Similar analysis between *Brassica*, rice and Arabidopsis showed 90 genes encoding MATH-domain proteins from *B. rapa*, 63 genes in Arabidopsis and 36 genes in rice (Zhao et al., 2013). Further, BTB superfamily has been characterized in various dicots species and comprises protein members from MATH-BTB family (Gingerich et al., 2007). Analysis of domains present in the MDCPs in both Arabidopsis and rice showed the presence of BTB domain along with the MATH domain. The BTB domain (POZ domain) has been earlier known for its protein-protein interaction modules with its ability to self-associate and also to interact with other non-BTB proteins (Stogios et al., 2005). As reported earlier, the BTB domain was also found at the carboxy-terminal in the MDC proteins in both Arabidopsis and rice. MDCP family members were earlier shown to mediate the interaction of BTB/POZ-MATH (BPM) proteins with ethylene response factor/Apetala2 transcription factor family members (Weber and Hellmann, 2009).

In this study, we show that MDC proteins along with BTB domain are found in large number in rice than in Arabidopsis. This large number of members in rice can be attributed to major expansion and diversification events in monocots including rice, which have probably occurred after the split of monocot and dicot (Gingerich et al., 2007). The low sequence conservation within the group signifies the evolution of monocots as a component of an innate immunity system owing to sophisticated mechanisms developed by the pathogens (Gingerich et al., 2005, 2007). Phylogenetic relationship tree of the MDC proteins in Arabidopsis and rice showed a distinct evolution of these proteins in plants. This shows that BTB domains in the MDC proteins might have been evolving distinctly to the MATH domain contributing to the overall distinctness to the MDC proteins having BTB domain. Previously, a phylogenetic analysis in mosses, eudicots, and grasses has shown that the expansion in MATH-BTB gene family occurred largely due to local gene duplications (Juranić and Dresselhaus, 2014). The localization of the MDC protein encoding genes in both Arabidopsis and rice shows that the MDC genes lie in a cluster on various chromosomes. Interestingly in rice, the maximum number (30) of genes coding for MDC proteins having BTB domains were found to be clustered on the chromosome X. However, one of the earlier studies showed MATH domain proteins as part of the syntenic region on chromosome VIII (Juranić and Dresselhaus, 2014). However, these proteins possessed only the BTB domain in their sequence and lacked MATH domain. In contrast, a large number of genes (24) encoding MDC protein were found clustered on the chromosome III in Arabidopsis which is known for the presence of clustered gene families (Salanoubat et al., 2000). Thus, the clade-specific expansion in MATH-BTB gene family occurred largely due to tandem or segmental duplications (Juranić and Dresselhaus, 2014).

Plants frequently encounter various biotic and abiotic stresses throughout their life cycle (Singh V. K. et al., 2015). The transcriptome analysis of the molecular response in plants toward multiple stresses (abiotic and biotic) has identified several overlapping genes which are identified and proposed to be responsible for generalized stress response or found to be the points of cross-talk between signaling pathways (Atkinson and Urwin, 2012; Kissoudis et al., 2014; Foyer et al., 2016). MDCPs of BTB superfamily, function as substrate-specific adaptors of CULLIN (CUL3)-based ubiquitin E3 ligase to target protein for ubiquitination (Weber et al., 2005). Ubiquitin significantly affects physiology, development and homeostasis of all eukaryotes including embryogenesis, cell cycle, hormonal balance, photomorphogenesis, circadian rhythms, flower development, self-incompatibility, ecological adaptation, disease resistance as well as cell death (Gingerich et al., 2007; Zapata et al., 2007; Qi et al., 2009; Zhao et al., 2013). Moreover, types of recognition motifs in BTB protein are mostly conserved between Arabidopsis and rice indicating that similar substrates exist in both the species (Gingerich et al., 2007; Juranić and Dresselhaus, 2014). Therefore, to gain preliminary insight into the potential function of plant MDCP genes during stress response and development, we have explored publicly available microarray data for Arabidopsis and rice. Expression analysis of MDCP gene family members using rice microarray data revealed that all the 11 highly expressed genes under biotic stress also showed high transcript levels in all the tissues as well as at all the development stages in rice. These findings highlight the role of MDCP genes in overall plant growth and development.

In order to analyze the correlated response under biotic and abiotic stress, MDC protein encoding genes which are highly up-regulated in all biotic stresses were analyzed for salt and drought stress response. Interestingly, these selected MDC genes showed positively correlated response for abiotic and biotic stress which further signifies the coordinated response of various gene families pertaining to various types of stress (abiotic or biotic). Similarly, BTB/POZ protein ETO1 (ethylene overproducer 1) was found to interact with ethylene biosynthesis protein ACS5 and negatively affects ethylene biosynthesis (Wang et al., 2004). In contrast, MATH-BTB proteins were also shown to directly interact with a class I homeodomain leucine zipper (HD-ZIP) transcription factor ATHB6, which negatively regulates ABA responses (Lechner et al., 2011). ABA regulates different phases of plant development including seed dormancy, germination, and reproduction and also acts as a key factor in biotic and abiotic stress responses in plants, particularly salinity and drought (Ton et al., 2009; Raghavendra et al., 2010). It was also reported earlier that MDC proteins located on the plasma membrane primarily respond to fungal infection in Arabidopsis roots and are also involved in nodule formation in Medicago (Oelmüller et al., 2005). Similarly, Cosson et al. (2010) found that one of the restricted TEV movement (RTM) genes i.e., RTM3 which restricts the long-distance movement of various potyviruses in Arabidopsis, encodes an unknown protein containing MATH domain in its amino-terminal region. In maize, MATH-BTB genes were shown to be expressed in zygote and control spindle length during meiosis as well as nuclei identity during first pollen mitosis (Juranič et al., 2012). An analysis suggested that some genes in the plants are universally stress responsive which leads to the evolution of effective strategies toward understanding the stress behavior in plants (Narsai et al., 2013). Earlier, disease resistant pathway similar to the Arabidopsis NPR1 (AtNPR1), which also showed negative effects on viral infections, showed negative regulation of this gene in plants under salt and drought stress response (Quilis et al., 2008). These observations indicate toward possibly diverse roles of MDCP genes throughout the plant development and stress response in rice.

## Conclusions

The strategy of comparative genomics and transcriptomics had led to the discovery of many novel genes and gene families playing a role in various stress responses. One of the members identified in such strategic analysis toward salt stress led to the identification of MATH-domain family which has been earlier known for their role in the plant/microbe interaction. Apart from characterizing the family in both Arabidopsis and rice, we have attempted to establish their role in overall plant growth and development as well as abiotic and biotic stresses using the high-throughput expression data available in the public domain. Further, we narrowed down 11 potential candidate genes in rice which showed higher expression in all the developmental stages, tissues, as well as biotic stresses in rice. These genes were further validated through qRT-PCR with drought and salinity stress in rice. Combining the publicly available data and our study, we identified *OsM4* and *OsMB11* as the potential candidate genes ubiquitously expressed in all the tissues, developmental stages, biotic as well as abiotic stresses. This needs to be comprehensively analyzed further for functional validation of their specific roles in plant development and stress response in increasing environmental resilience in crops.

## Author contributions

SLS-P, AP conceived the idea and designed the experiments. RJ did the real time PCR work and its analysis. HK performed the MPSS and microarray database analysis. RJ, HK wrote the manuscript. SLS-P, AP edited the manuscript. All the authors approved the final manuscript.

### Conflict of interest statement

The authors declare that the research was conducted in the absence of any commercial or financial relationships that could be construed as a potential conflict of interest.

## Supplementary material

Figure S1 **Representative (unscaled) domain architecture of the MDC proteins in Arabidopsis and rice**. All the MDC proteins in rice and Arabidopsis were found to consist of MATH domain (PF00917) while few MDC proteins in both Arabidopsis and rice were found to contain BTB domains (PF00651) also.

Figure S2 **Multiple sequence alignment of full length sequences having single MATH domain in Arabidopsis and rice**. The sequence analysis was performed using Seaview (version 4) multiple sequence alignment editor (Gouy et al., 2010).

Figure S3 **Multiple sequence alignment of full length sequences having two MATH domains in Arabidopsis and rice**. The sequence analysis was performed using Seaview (version 4) multiple sequence alignment editor (Gouy et al., 2010).

Figure S4 **Multiple sequence alignment of full length sequences having MATH and BTB domains in Arabidopsis and rice**. The sequence analysis was performed using Seaview (version 4) multiple sequence alignment editor (Gouy et al., 2010).

Figure S5 **Heatmap representation of the expression of MDC protein encoding genes in response to various abiotic stresses (A) such as cold, drought, genotoxic, heat, osmotic, oxidative, salinity and wound in Arabidopsis and (B) salinity, heat, drought and cold in rice**. The expression values were obtained from Affymetrix array databases using Genevestigator Response Viewer (https://www.genevestigator.com). For Arabidopsis, 22 K ATH1 genome array was chosen along with pre-existing microarray and in case of rice, microarray results of OS_51 K: Rice Genome 51 K pre-existing microarrays were chosen. The details of the libraries used in the current are presented in Table S3.

Table S1 **List of libraries of different tissues with their abbreviations used in the expression analysis of MDC protein encoding genes in (a) rice and (b) Arabidopsis**.

Table S2 **List of libraries of different developmental stages with their abbreviations used in the expression analysis of MDC protein encoding genes in (a) rice and (b) Arabidopsis**.

Table S3 **List of libraries of various abiotic stresses with their abbreviations used in the expression analysis of MDC protein encoding genes in (a) rice and (b) Arabidopsis**.

Table S4 **List of libraries of various biotic stresses with their abbreviations used in the expression analysis of MDC protein encoding genes in (a) rice and (b) Arabidopsis**.

Table S5 **List of primers used for qRT-PCR analysis in the present study**.
