# Supplementary figures and images for: MATH-Domain Family Shows Response toward Abiotic Stress in Arabidopsis and Rice
Source: Front Plant Sci. 2016 Jun 28;7:923. doi: 10.3389/fpls.2016.00923 (PMC4923191; doi:10.3389/fpls.2016.00923)

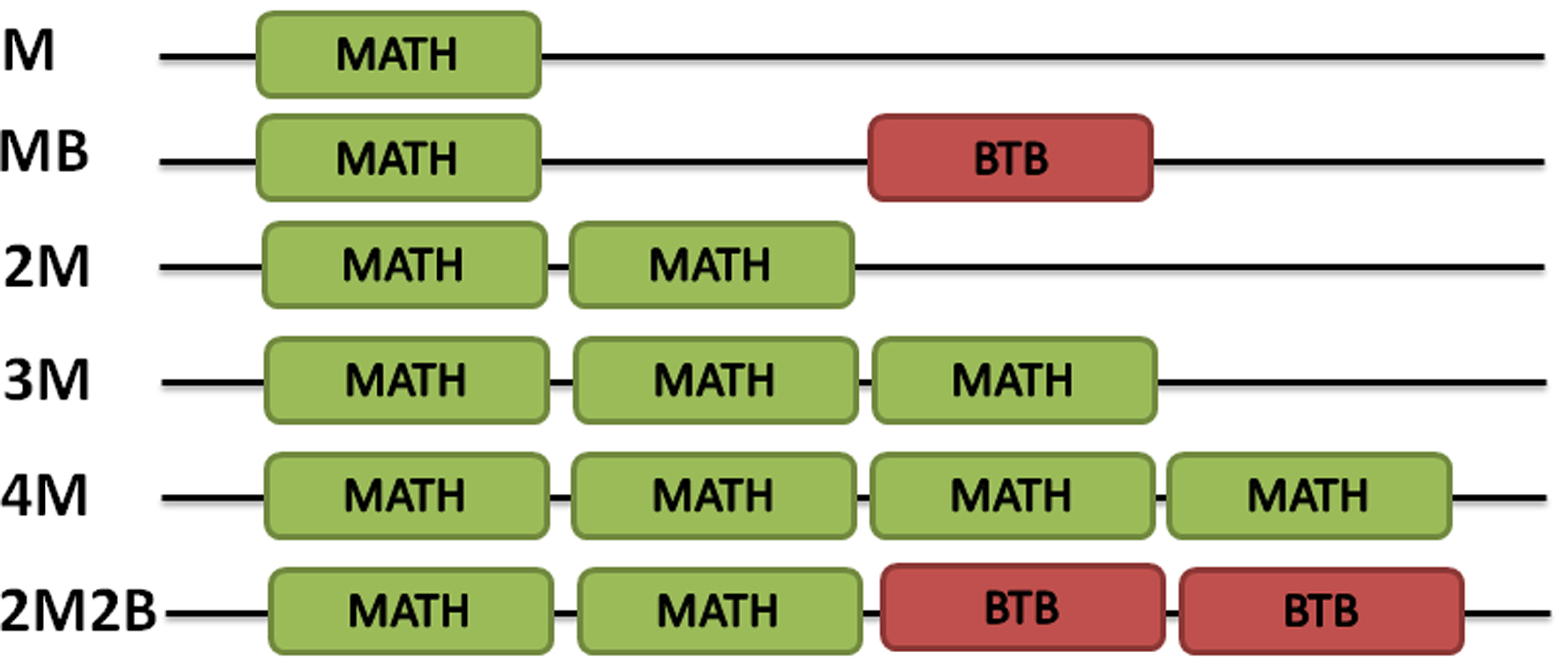

Supplement: Figure S1 — Representative (unscaled) domain architecture of the MDC proteins in Arabidopsis and rice. All the MDC proteins in rice and Arabidopsis were found to consist of MATH domain (PF00917) while few MDC proteins in both Arabidopsis and rice were found to contain BTB domains (PF00651) also. [file Image1.TIF]

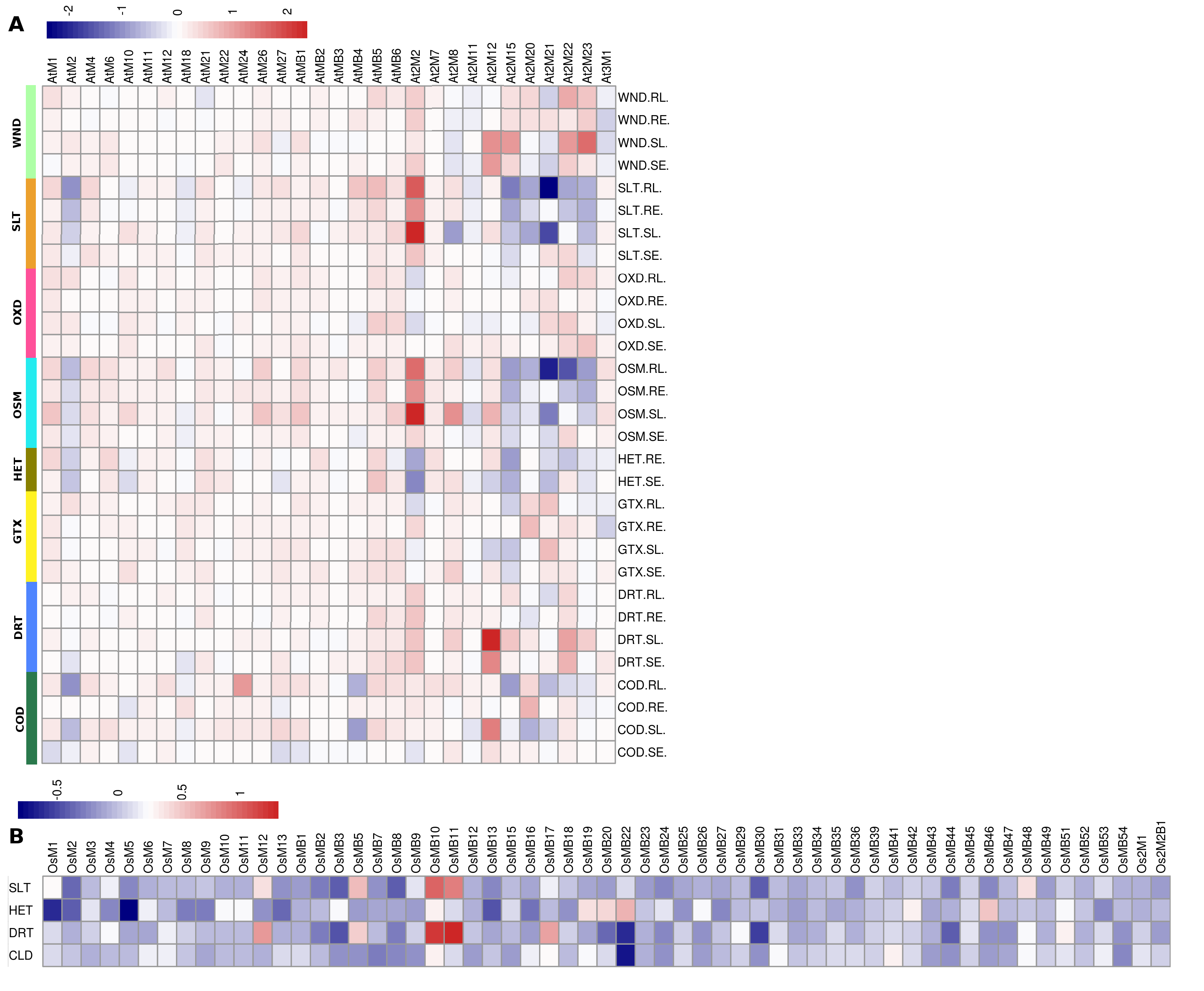

Supplement: Figure S5 — Heatmap representation of the expression of MDC protein encoding genes in response to various abiotic stresses (A) such as cold, drought, genotoxic, heat, osmotic, oxidative, salinity and wound in Arabidopsis and (B) salinity, heat, drought and cold in rice. The expression values were obtained from Affymetrix array databases using Genevestigator Response Viewer (https://www.genevestigator.com). For Arabidopsis, 22 K ATH1 genome array was chosen along with pre-existing microarray and in case of rice, microarray results of OS_51 K: Rice Genome 51 K pre-existing microarrays were chosen. The details of the libraries used in the current are presented in Table S3. [file Image5.TIF]
