# Supplementary material for: MATH-Domain Family Shows Response toward Abiotic Stress in Arabidopsis and Rice
Source: Front Plant Sci. 2016 Jun 28;7:923. doi: 10.3389/fpls.2016.00923 (PMC4923191; doi:10.3389/fpls.2016.00923)
Supplement: Figure S2 — Multiple sequence alignment of full length sequences having single MATH domain in Arabidopsis and rice. The sequence analysis was performed using Seaview (version 4) multiple sequence alignment editor (Gouy et al., 2010). [file Image2.PDF]

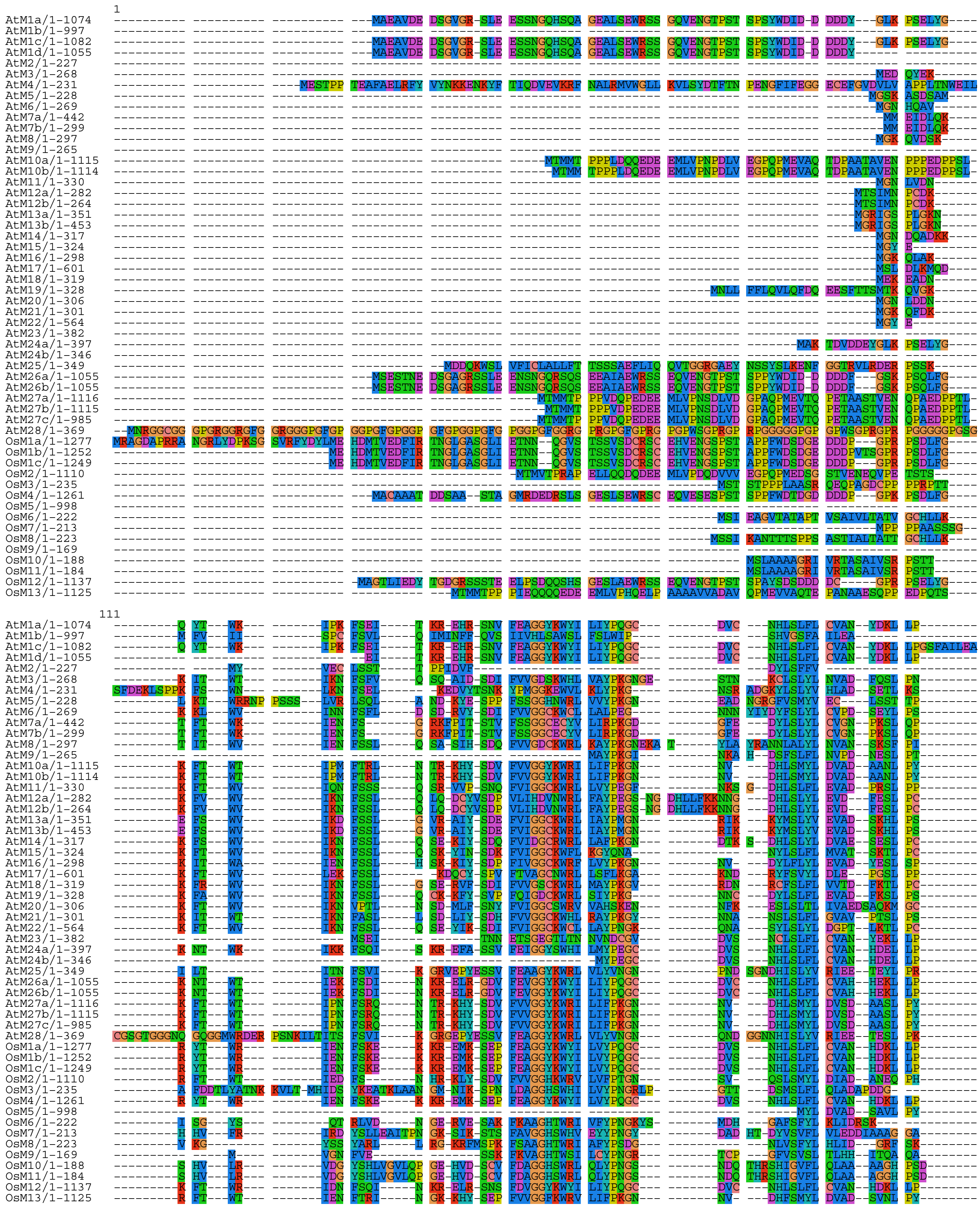

1
AtM1a/1-1074
AtM1b/1-997
AtM1c/1-1082
AtM1d/1-1055
AtM2/1-227
AtM3/1-268
AtM4/1-231
AtM5/1-228
AtM6/1-269
AtM7a/1-442
AtM7b/1-299
AtM8/1-297
AtM9/1-265
AtM10a/1-1115
AtM10b/1-1114
AtM11/1-330
AtM12a/1-282
AtM12b/1-264
AtM13a/1-351
AtM13b/1-453
AtM14/1-317
AtM15/1-324
AtM16/1-298
AtM17/1-601
AtM18/1-319
AtM19/1-328
AtM20/1-306
AtM21/1-301
AtM22/1-564
AtM23/1-382
AtM24a/1-397
AtM24b/1-346
AtM25/1-349
AtM26a/1-1055
AtM26b/1-1055
AtM27a/1-1116
AtM27b/1-1115
AtM27c/1-985
AtM28/1-369
OsM1a/1-1277
OsM1b/1-1252
OsM1c/1-1249
OsM2/1-1110
OsM3/1-235
OsM4/1-1261
OsM5/1-998
OsM6/1-222
OsM7/1-213
OsM8/1-223
OsM9/1-169
OsM10/1-188
OsM11/1-184
OsM12/1-1137
OsM13/1-1125
111

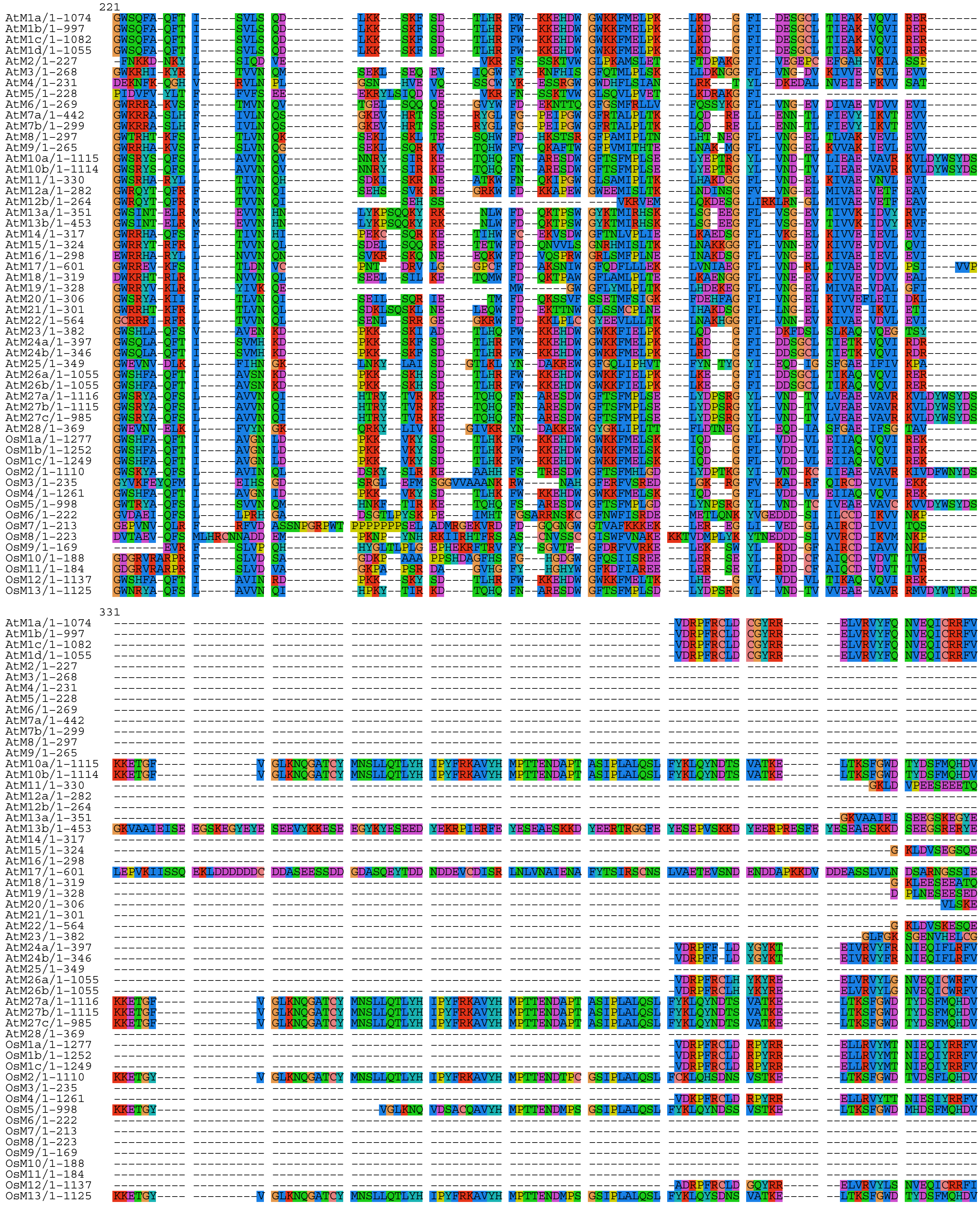

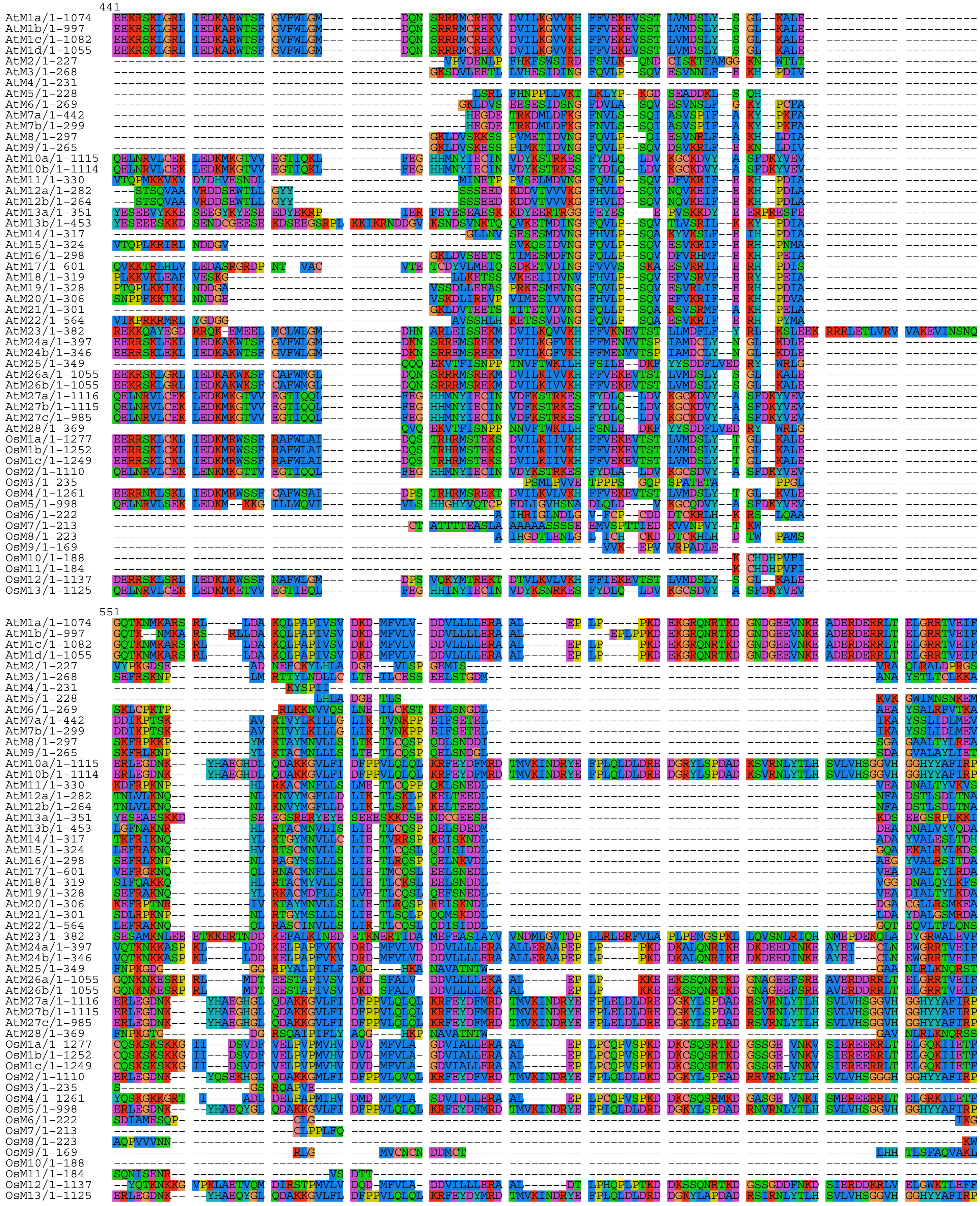
441
AtM1a/1-1074
AtM1b/1-997
AtM1c/1-1082
AtM1d/1-1055
AtM2/1-227
AtM3/1-268
AtM4/1-231
AtM5/1-228
AtM6/1-269
AtM7a/1-442
AtM7b/1-299
AtM8/1-297
AtM9/1-265
AtM10a/1-1115
AtM10b/1-1114
AtM11/1-330
AtM12a/1-282
AtM12b/1-264
AtM13a/1-351
AtM13b/1-453
AtM14/1-317
AtM15/1-324
AtM16/1-298
AtM17/1-601
AtM18/1-319
AtM19/1-328
AtM20/1-306
AtM21/1-301
AtM22/1-564
AtM23/1-382
AtM24a/1-397
AtM24b/1-346
AtM25/1-349
AtM26a/1-1055
AtM26b/1-1055
AtM27a/1-1116
AtM27b/1-1115
AtM27c/1-985
AtM28/1-369
OsM1a/1-1277
OsM1b/1-1252
OsM1c/1-1249
OsM2/1-1110
OsM3/1-235
OsM4/1-1261
OsM5/1-998
OsM6/1-222
OsM7/1-213
OsM8/1-223
OsM9/1-169
OsM10/1-188
OsM11/1-184
OsM12/1-1137
OsM13/1-1125
551

```
            661
AtM1a/1-1074   ILSHIFS--T K--------- ----IEVAHQ EAIALKRQEE LIR-EEEEAW LAET----EQ RAKR--GAAE REKKSKKKQA KQKRNKNKGK DKRKEEKVSF ATHAK---DL
AtM1b/1-997    ILSHIFS--T K--------- ----IEVAHQ EAIALKRQEE LIR-EEEEAW LAET----EQ RAKR--GAAE REKKSKKKQA KQKRNKNKGK DKRKEEKVSF ATHAKDLEEN
AtM1c/1-1082   ILSHIFS--T K--------- ----IEVAHQ EAIALKRQEE LIR-EEEEAW LAET----EQ RAKR--GAAE REKKSKKKQA KQKRNKNKGK DKRKEEKVSF ATHAK---DL
AtM1d/1-1055   ILSHIFS--T K--------- ----IEVAHQ EAIALKRQEE LIR-EEEEAW LAET----EQ RAKR--GAAE REKKSKKKQA KQKRNKNKGK DKRKEEKVSF ATHAK---DL
AtM2/1-227     KHKTVWL--- ---------- -----QQWIM AATKARGIPQ SLSLADLQEA YLDE------ ---------- ---------- ---------- ---------- ----------
AtM3/1-268     GFKLDWL--- ---------- -----EKKLK EVCEARVQEI DEEWKDLTDL KEKF------ ---------- ---------- ---------- ---------- ----------
AtM4/1-231     ---------- ---------- ---------- ---------- ---------- ---------- ---------- ---------- ---------- ---------- ----------
AtM5/1-228     GLPQSMS--- ---------- ---------- ---FDKIEGA YLDREGTLEV EIEC------ ---------- ---------- ---------- ---------- ----------
AtM6/1-269     GFKLDWL--- ---------- -----EKKLK E--------- -TGKSRLQEI EEDL------ ---------- ---------- ---------- ---------- ----------
AtM7a/1-442    GFRVKWL--- ---------- -----KSKLD EISLARKKKV DADAARVQEL EGKVKNQVEL DKEKTKSHVE PKMLSFKDYA SPLQEEGFLE NNKLIIRVEV KVAEE-----
AtM7b/1-299    GFRVKWL--- ---------- -----KSKLD EISLARKKKV DADAARVQEL EGKV------ ---------- ---------- ---------- ---------- ----------
AtM8/1-297     GFKLDWL--- ---------- -----EKKHG EIK-EKKKKE EASLKRLQEM EKQI------ ---------- ---------- ---------- ---------- ----------
AtM9/1-265     GLKLDWL--- ---------- -----EKKLD ELK-EKKKKE ESCLVRLREM DEQL------ ---------- ---------- ---------- ---------- ----------
AtM10a/1-1115  TLSDQWYKFD D--------- -----ERVTK EDVKRALEEQ YGGEEELPQN NPGF----NN PPFKFTKYSN AYMLVYIRES DKDKIICNVD EKDIAEHLRV RLKKEQEEKE
AtM10b/1-1114  TLSDQWYKFD D--------- -----ERVTK EDVKRALEEQ YGGEEELPQN NPGF----NN PPFKFTKYSN AYMLVYIRES DKDKIICNVD EKDIAEHLRV RLKKEQEEKE
AtM11/1-330    GFKVDWL--- ---------- -----EKKLE EVK-KKKEEE QTGEARIQEL EEEL------ ---------- ---------- ---------- ---------- ----------
AtM12a/1-282   GFMLDWL--- ---------- -----RLKLN RAL-EKQIAC DAG------- ---------- ---------- ---------- ---------- ---------- ----------
AtM12b/1-264   GFMLDWL--- ---------- -----RLKLN RAL-EKQIAC DAG------- ---------- ---------- ---------- ---------- ---------- ----------
AtM13a/1-351   KRNDDGV--- ---------- ---KSNDSVN KTQQVKETMD INGFQVLPSQ VSGN------ ---------- ---------- ---------- ---------- ----------
AtM13b/1-453   GFKVDWL--- ---------- -----DKKLK EVK-EKKVVE QTGKTRIQEL EEEL------ ---------- ---------- ---------- ---------- ----------
AtM14/1-317    GFKLDWL--- ---------- -----KKKLD QVT-QKKEKE AAGETRMHEI GEEL----KD LKLKCSDLEA QLDKV----- ---------- ---------- ----------
AtM15/1-324    DFKVDWL--- ---------- -----ERKLE EVK-EKKMEE QIGKSRMQEL EEEL------ ---------- ---------- ---------- ---------- ----------
AtM16/1-298    GFKVDWL--- ---------- -----EKKLV EIS-EKKNKE EAGETRMQEI NEEL------ ---------- ---------- ---------- ---------- ----------
AtM17/1-601    GFKVDWL--- ---------- -----EKKLD QLK-EKKEEE MSGLARLHEI EENLVILKQK WSDLGALAEK EKADFVASKT EVSNNENDYA NKEDVDDEAS SL--------
AtM18/1-319    GFKVDWL--- ---------- -----EKKLE EVK-EKKKEE QIGETRMQEM KV-------- ---------- ---------- ---------- ---------- ----------
AtM19/1-328    GFKVDWL--- ---------- -----EKKLD QVR-DKKEKE RSCLAKLQET EETL------ ---------- ---------- ---------- ---------- ----------
AtM20/1-306    GFKLDWL--- ---------- -----EKKLN EVL-EKKEKE ESYETRMREI EEEM------ ---------- ---------- ---------- ---------- ----------
AtM21/1-301    GFKLDWL--- ---------- -----EKKLY EVS-EKKENE EASETGLQEM EEEL------ ---------- ---------- ---------- ---------- ----------
AtM22/1-564    GFKVDWL--- ---------- -----ERKLE EVK-EKKIQE HIGKSRMQGL EEDL----KV FKKKCSDIEA LLEKEKEELK GLKQKCSDIE ALLEKEKGKV LAAAARTPLT
AtM23/1-382    VLDHIFC--- ---------- --NKIEFTYE ETIALKRQEE LIHEEEAE-- ---------- ---------- ---------- ---------- ---------- ----------
AtM24a/1-397   VLDHIFS--- ---------- ---KIEVAYK EAIALKRQEE LIREEEETSK RSKV------ ---------- ---------- ---------- ---------- ----------
AtM24b/1-346   VLDHIFS--- ---------- ---KIEVAYK EAIALKRQEE LIREEEETSK RSKV------ ---------- ---------- ---------- ---------- ----------
AtM25/1-349    NHRQIYT--- ---------- ----AAWYPI GSGYGVGVNN IILLADLNDA SQGY------ ---------- ---------- ---------- ---------- ----------
AtM26a/1-1055  VLAHIFS--N K--------- ----IEVAYQ EAIAWKRQEE LIR-EEEEAW LAES----EQ KGKR--GASE KEKKSKKKQA KQKKNKNKGK EMRKEDKVRT QTEER---EI
AtM26b/1-1055  VLAHIFS--N K--------- ----IEVAYQ EAIAWKRQEE LIR-EEEEAW LAES----EQ KGKR--GASE KEKKSKKKQA KQKKNKNKGK EMRKEDKVRT QTEER---EI
AtM27a/1-1116  TLSDQWYKFD D--------- -----ERVTK EDLKRALEEQ YGGEEELPQT NPGF---NNN PPFKFTKYSN AYMLVYIRES DKDKIICNVD EKDIAEHLRV RLKKEQEEKE
AtM27b/1-1115  TLSDQWYKFD D--------- -----ERVTK EDLKRALEEQ YGGEEELPQT NPGF---NNN PPFKFTKYSN AYMLVYIRES DKDKIICNVD EKDIAEHLRV RLKKEQEEKE
AtM27c/1-985   TLSDQWYKFD D--------- -----ERVTK EDLKRALEEQ YGGEEELPQT NPGF---NNN PPFKFTKYSN AYMLVYIRES DKDKIICNVD EKDIAEHLRV RLKKEQEEKE
AtM28/1-369    NHAQIYC--- ---------- -------KY- ---------- ---------- ---------- ---------- ---------- ---------- ---------- ----------
OsM1a/1-1277   ALSHIFS--- G--------- ----IEVAYQ EAVALKRQEE LIR-EEEEAW LLEN----EM KGKR-GSTTE KDKRAKKKQA KQKKNNRKVK DKDREEKCDS NFPERSQDEN
OsM1b/1-1252   ALSHIFS--- G--------- ----IEVAYQ EAVALKRQEE LIR-EEEEAW LLEN----EM KGKR-GSTTE KDKRAKKKQA KQKKNNRKVK DKDREEKCDS NFPERSQDEN
OsM1c/1-1249   ALSHIFS--- G--------- ----IEVAYQ EAVALKRQEE LIR-EEEEAW LLEN----EM KGKR-GSTTE KDKRAKKKQA KQKKNNRKVK DKDREEKCDS NFPERSQDEN
OsM2/1-1110    ALSDIWYKFD D--------- -----ERVTK EDMKRALEEQ YGGEEELPHT NPGL----NT TPLRFTKHSN AYMLVYIRES DREKIICNLD ENDIPEHLKI RLRKENEERE
OsM3/1-235     ---------- ---------- ---------- ----RAPRTA TSRSSPSPSW RMEIP----- ---------- ---------- ---------- ---------- ----------
OsM4/1-1261    VLSHIFS--- G--------- ----IEVAYQ EAVALKRQEE LIREEEEEAW LLGN----EM KGKRGGGANE KDKRAKKKQA KQKKNNRKIK DKERDEKFEA KILERLHDET
OsM5/1-998     TLSDQWFKFD D--------- -----ERVTK EDAKRALEEQ YGGEEELPQT NPGL----NN TPFKFTKYSN AYMLVYIRES DKDKIICNVD EKDIAEHLRI RLEKDREEKE
OsM6/1-222     GFKRLFS--- ---------- ---------- ---------- ---------- --------YF LA-------- ---------- ---------- ---------- ----------
OsM7/1-213     ---------- ---------- ---------- ---------- ---------- ---------- ---------- ---------- ---------- ---------- ----------
OsM8/1-223     AFARLFS--- ---------- ---------- ---------C FLA------- ---------- ---------- ---------- ---------- ---------- ----------
OsM9/1-169     KFLEFFL--- ---------- ---------- ---------- ---------- ---------- ---------- ---------- ---------- ---------- ----------
OsM10/1-188    --SQNFS--- ---------- -----ENRVS DAT------- ---------- ---------- ---------- ---------- ---------- ---------- ----------
OsM11/1-184    ---------- ---------- ---------- ---------- ---------- ---------- ---------- ---------- ---------- ---------- ----------
OsM12/1-1137   ALAHIFSSSF GCLVFSPYKS CISRIEVAYQ EAVALKRQEE LIREEEAAGL AEIE------ -LKAKRSAAE KEKRIRKKQA KQKKNSRKNN KGKNEKVDIK EIVIESSSPP
OsM13/1-1125   TLSDQWYKFD D--------- -----ERVTK EDTKKALEEQ YGGEEELPQI NPGF----NN TPFKFTKYSN AYMLVYIRES DKDKIMCNVD EKDIAEHLRI RLKKEQEEKE

            771
AtM1a/1-1074   EENQNQNQND EEEKDSVTEK AQSSAEKPDT LGDV------ ------SDIS DSVDGSADIL QPDLEDRDSS SVLWDTDALE IHPPSSEGSS RGRGISISTP NGITEGKSHS
AtM1b/1-997    QNQNQNDEEE KDSVTEKAQS SAEKPDTLGD V--------- ------SDIS DSVDGSADIL QPDLEDRDSS SVLWDTDALE IHPPSSEGSS RGRGISISTP NGITEGKSHS
AtM1c/1-1082   EENQNQNQND EEEKDSVTEK AQSSAEKPDT LGDV------ ------SDIS DSVDGSADIL QPDLEDRDSS SVLWDTDALE IHPPSSEGSS RGRGISISTP NGITEGKSHS
AtM1d/1-1055   EENQNQNQND EEEKDSVTEK AQSSAEKPDT LGDV------ ------SDIS DSVDGSADIL QPDLEDRDSS SVLWDTDALE IHPPSSEGSS RGRGISISTP NGITEGKSHS
AtM2/1-227     ---------- ---------- ---------- ---------- ---------- ---------- ---------- ---------- ---------- ---------- ----------
AtM3/1-268     ---------- ---------- ---------- ---------- ---------- ---------- ---------- ---------- ---------- ---------- ----------
AtM4/1-231     ---------- ---------- ---------- ---------- ---------- ---------- ---------- ---------- ---------- ---------- ----------
AtM5/1-228     ---------- ---------- ---------- ---------- ---------- ---------- ---------- ---------- ---------- ---------- ----------
AtM6/1-269     ---------- ---------- ---------- ---------- ---------- ---------- ---------- ---------- ---------- ---------- ----------
AtM7a/1-442    ---------- ---------- ---------- ---------- ---------- ---------- ---------- ---------- ---------- ---------- ----------
AtM7b/1-299    ---------- ---------- ---------- ---------- ---------- ---------- ---------- ---------- ---------- ---------- ----------
AtM8/1-297     ---------- ---------- ---------- ---------- ---------- ---------- ---------- ---------- ---------- ---------- ----------
AtM9/1-265     ---------- ---------- ---------- ---------- ---------- ---------- ---------- ---------- ---------- ---------- ----------
AtM10a/1-1115  DKRKYKAQAH LFTTIKVARD DDITEQI--- ---------- ---------- -GKNIYFDLV DHEKVRSFRI QKQTPFQQFK EEVAKEFGVP VQLQRFWIWA KRQNHTYRPN
AtM10b/1-1114  DKRKYKAQAH LFTTIKVARD DDITEQI--- ---------- ---------- -GKNIYFDLV DHEKVRSFRI QKQTPFQQFK EEVAKEFGVP VQLQRFWIWA KRQNHTYRPN
AtM11/1-330    ---------- ---------- ---------- ---------- ---------- ---------- ---------- ---------- ---------- ---------- ----------
AtM12a/1-282   ---------- ---------- ---------- ---------- ---------- ---------- ---------- ---------- ---------- ---------- ----------
AtM12b/1-264   ---------- ---------- ---------- ---------- ---------- ---------- ---------- ---------- ---------- ---------- ----------
AtM13a/1-351   ---------- ---------- ---------- ---------- ---------- ---------- ---------- ---------- ---------- ---------- ----------
AtM13b/1-453   ---------- ---------- ---------- ---------- ---------- ---------- ---------- ---------- ---------- ---------- ----------
AtM14/1-317    ---------- ---------- ---------- ---------- ---------- ---------- ---------- ---------- ---------- ---------- ----------
AtM15/1-324    ---------- ---------- ---------- ---------- ---------- ---------- ---------- ---------- ---------- ---------- ----------
AtM16/1-298    ---------- ---------- ---------- ---------- ---------- ---------- ---------- ---------- ---------- ---------- ----------
AtM17/1-601    ---------- ---------- ---------- ---------- ---------- ---------- ---------- ---------- ---------- -----VWTDS ARDTKTGGKA
AtM18/1-319    ---------- ---------- ---------- ---------- ---------- ---------- ---------- ---------- ---------- ---------- ----------
AtM19/1-328    ---------- ---------- ---------- ---------- ---------- ---------- ---------- ---------- ---------- ---------- ----------
AtM20/1-306    ---------- ---------- ---------- ---------- ---------- ---------- ---------- ---------- ---------- ---------- ----------
AtM21/1-301    ---------- ---------- ---------- ---------- ---------- ---------- ---------- ---------- ---------- ---------- ----------
AtM22/1-564    LDRMNILIGY REMISLTKLN AKKGGFLVNN KVKIVVEVDV LQVVGGLDVS QEVTQPLKRI RLNDDGAAST HLQTIDVNGF LVLASQVESV KRIFESHPEM TLTFRANNQQ
AtM23/1-382    ---------- ---------- ---------- ---------- ---------- ---------- ---------- ---------- ---------- ---------- ----------
AtM24a/1-397   ---------- ---------- ---------- ---------- ---------- ---------- ---------- ---------- ---------- ---------- ----------
AtM24b/1-346   ---------- ---------- ---------- ---------- ---------- ---------- ---------- ---------- ---------- ---------- ----------
AtM25/1-349    ---------- ---------- ---------- ---------- ---------- ---------- ---------- ---------- ---------- ---------- ----------
AtM26a/1-1055  ---------- -EKEECVRAI AESSAEKPDT LGDV------ ------SDVS DSVDSSAEIL QLDSEDRESS PVHWEMDASE VHPPSAGDTS RGRGNSFSIP NGVAERKGLS
AtM26b/1-1055  ---------- -EKEECVRAI AESSAEKPDT LGDV------ ------SDVS DSVDSSAEIL QLDSEDRESS PVHWEMDASE VHPPSAGDTS RGRGNSFSIP NGVAERKGLS
AtM27a/1-1116  DKRRYKAQAH LYTIIKVARD EDLKEQI--- ---------- ---------- -GKDIYFDLV DHDKVRSFRI QKQTPFQQFK EEVAKEFGVP VQLQRFWIWA KRQNHTYRPN
AtM27b/1-1115  DKRRYKAQAH LYTIIKVARD EDLKEQI--- ---------- ---------- -GKDIYFDLV DHDKVRSFRI QKQTPFQQFK EEVAKEFGVP VQLQRFWIWA KRQNHTYRPN
AtM27c/1-985   DKRRYKAQAH LYTIIKVARD EDLKEQI--- ---------- ---------- -GKDIYFDLV DHDKVRSFRI QKQTPFQQFK EEVAKEFGVP VQLQRFWIWA KRQNHTYRPN
AtM28/1-369    ---------- ---------- ---------- ---------- ---------- ---------- ---------- ---------- ---------- ---------- ----------
OsM1a/1-1277   TIHDRE--DS KQAGQISMKV DTSEEGA--- ---------- ------SDVS DNLDGSIEI- -----QKKHS TMENKSLSCS SESATMNNAQ GKINNLLESK DQISRNRGKA
OsM1b/1-1252   TIHDRE--DS KQAGQISMKV DTSEEGA--- ---------- ------SDVS DNLDGSIEI- -----QKKHS TMENKSLSCS SESATMNNAQ GKINNLLESK DQISRNRGKA
OsM1c/1-1249   TIHDRE--DS KQAGQISMKV DTSEEGA--- ---------- ------SDVS DNLDGSIEI- -----QKKHS TMENKSLSCS SESATMNNAQ GKINNLLESK DQISRNRGKA
OsM2/1-1110    YKKKEKAEAH MFTALKVARD SDFAEQI--- ---------- ---------- -GKHIYFDLV DYDNIQSLRA PKHLTINQAK VDLSKEFGIP VQSQRFWLWA KRQNHTFRPV
OsM3/1-235     ---------- ---------- ---------- ---------- ---------- ---------- ---------- ---------- ---------- ---------- ----------
OsM4/1-1261    AIDDSDGLSS KQAEEVTTKV ENLEEGASDR QGDLDSSEIA HRPDSGDKYP RQMNGLSDVT GNAQKVKKAS SMEANSPVFL ADSVAASGTH SRGNNLSDSK NRMTPNRGKN
OsM5/1-998     RRKKEKAEAH LYTIIKVARD DDLTTQI--- ---------- ---------- -GKDIYFDLV DHDKVPSFRI QKQMPFTQFK EEVAKEFGIP TQFQRFWLWA KRQNHTYRPN
OsM6/1-222     ---------- ---------- ---------- ---------- ---------- ---------- ---------- ---------- ---------- ---------- ----------
OsM7/1-213     ---------- ---------- ---------- ---------- ---------- ---------- ---------- ---------- ---------- ---------- ----------
OsM8/1-223     ---------- ---------- ---------- ---------- ---------- ---------- ---------- ---------- ---------- ---------- ----------
OsM9/1-169     ---------- ---------- ---------- ---------- ---------- ---------- ---------- ---------- ---------- ---------- ----------
OsM10/1-188    ---------- ---------- ---------- ---------- ---------- ---------- ---------- ---------- ---------- ---------- ----------
OsM11/1-184    ---------- ---------- ---------- ---------- ---------- ---------- ---------- ---------- ---------- ---------- ----------
OsM12/1-1137   DDRILDDLSS QAEEITSNAD NPEEV----- ---------- ------SDIS DNRDDSSELL HNDLEDSESS PVNWETDASE TQATVPEGGE ------MQNE HAGKRTSFLD
OsM13/1-1125   HKKKEKAEAH LYTIIKVARD EDLKEQI--- ---------- ---------- -GKNIYFDLV DHEKVRSFRI QKQLPFTSFK EEVAKECGIP VQFQRFWLWA KRQNHTYRPN
```

```
             881
AtM1a/1-1074  TMDDSSSTCS NDSIRSGVTN GSYQGNS--- ---------LN FRNQKSPNKG KNQQVKAMTD AHSLASETDD QPSTL----G TDPKGQNYSS EASNVGESDW VVVSHIQEPE
AtM1b/1-997   TMDDSSSTCS NDSIRSGVTN GSYQGNS--- ---------LN FRNQKSPNKG KNQQVKAMTD AHSLASETDD QPSTL----G TDPKGQNYSS EASNVGESDW VVVSHIQEPE
AtM1c/1-1082  TMDDSSSTCS NDSIRSGVTN GSYQGNS--- ---------LN FRNQKSPNKG KNQQVKAMTD AHSLASETDD QPSTL----G TDPKGQNYSS EASNVGESDW VVVSHIQEPE
AtM1d/1-1055  TMDDSSSTCS NDSIRSGVTN GSYQGNS--- ---------LN FRNQKSPNKG KNQQVKAMTD AHSLASETDD QPSTL----G TDPKGQNYSS EASNVGESDW VVVSHIQEPE
AtM2/1-227    ---------- ---------- ---------- ---------- ---------- ---------- ---------- ---------- ---------- DTLNVEIECE VVNSRKMF--
AtM3/1-268    ---------- ---------- ---------- ---------- ---------- ---------- ---------- ---------- ---------- ---------- ----------
AtM4/1-231    ---------- ---------- ---------- ---------- ---------- ---------- ---------- ---------- ---------- ---------- ----------
AtM5/1-228    ---------- ---------- ---------- ---------- ---------- ---------- ---------- ---------- ---------- ---------E IENSHKNHPF
AtM6/1-269    ---------- ---------- ---------- ---------- ---------- ---------- ---------- ---------- KDLKVKCADM DALLEFLR-- ----------
AtM7a/1-442   ---------- ---------- ---------- ---------- -----GYLTG KEMFEIKGFE VPSSQVISVS QLFMKHPDIA TGFKLNGKGL KTAYMSLLLS LIETLRRPPL
AtM7b/1-299   ---------- ---------- ---------- ---------- ---------- ---------- --------KN QVELDKEKTK SHVEPKMLSF KDYASPVSPW IPRRKRSD--
AtM8/1-297    ---------- ---------- ---------- ---------- ---------- ---------- ---------- ---------- ---------- FNEAQIYKEK VLAARAPLSL
AtM9/1-265    ---------- ---------- ---------- ---------- ---------- ---------- ---------- ---------- QPFKKRCLDI EDQISKEKEE LL--AAREPL
AtM10a/1-1115 RPLSPNEELQ TVGQIREASN KANNAELKLF LEIERGPDDL PIPPPEKTSE DILLFFKLYD PENAVLRYVG RLMVKSSSKP MDIVGQLNKM AGFAPDEEIE LFEEIKFEPC
AtM10b/1-1114 RPLSPNEELQ TVGQIREASN KANNAELKLF LEIERGPDDL PIPPPEKTSE DILLFFKLYD PENAVLRYVG RLMVKSSSKP MDIVGQLNKM AGFAPDEEIE LFEEIKFEPC
AtM11/1-330   ---------- ---------- ---------- ---------- ---------- ---------- ---------- ---------- KEFKQKCLDR EAMLEKEKAK VL--TARAPL
AtM12a/1-282  ---------- ---------- ---------- ---------- ---------- ---------- ---------- ---------- ---------- ---------- ----------
AtM12b/1-264  ---------- ---------- ---------- ---------- ---------- ---------- ---------- ---------- ---------- ---------- ----------
AtM13a/1-351  ---------- ---------- ---------- ---------- ---------- ---------- ---------- ---------- FKLLRHCYHL TLVDFGTKGV TLLNTEKM--
AtM13b/1-453  ---------- ---------- ---------- ---------- ---------- ---------- ---------- ---------- KEFKQKCLDR EALLEKEKAK VL--AARASL
AtM14/1-317   ---------- ---------- ---------- ---------- ---------- ---------- ---------- ---------- ---KWKCSDL EAQLVKEKAD VLAAIAPLGS
AtM15/1-324   ---------- ---------- ---------- ---------- ---------- ---------- ------KIFK QKCSDIEAQL EKEKQKCSDI EALLEKEKAK SLAAARAPPL
AtM16/1-298   ---------- ---------- ---------- ---------- ---------- ---------- ---------- ---------- ESLKQKCLDL EAQLEKEKAE VF--AAKSPL
AtM17/1-601   FNNVASVAET SNNVLTEIPP AKETTDVNGF EVFMSQTKLD EVSRKKKMEQ GSGPRLQTME ER-------- ---------L QKLKLLFVDL ESQLQKEKVE AL--VARAPL
AtM18/1-319   ---------- ---------- ---------- ---------- ---------- ---------- ---------- ---------- --FKQKCSDI EALMEREKSK LLV-TRGSPL
AtM19/1-328   ---------- ---------- ---------- ---------- ---------- ---------- ---------- ---------- LKLKQKCTEL DALMDTEKAE L--SAIRTPL
AtM20/1-306   ---------- ---------- ---------- ---------- ---------- ---------- ---------- ---------- KDLKAKALDV GA-------- --------PL
AtM21/1-301   ---------- ---------- ---------- ---------- ---------- ---------- ---------- ---------- KDMKQKCLEM EALVEKEKAK V--STAKAPI
AtM22/1-564   LRTSCINVLL NLIETMCMSP QNLSIADLGQ TEQALTYLKN SGFKVDWLER KLEEVTEKKI QEHIGKSRMQ GLEEDLKEEL KDLKQKCSDT EALLEKEKTK VLAAARAPPL
AtM23/1-382   ---------- ---------- ---------- ---------- ---------- ---------- ---------- ---------- -----KNTKL KKTFVGKKVN ----------
AtM24a/1-397  ---------- ---------- ---------- ---------- ---------- ---------- ---------- ---------L KKGFGKCRLN ---------- ----------
AtM24b/1-346  ---------- ---------- ---------- ---------- ---------- ---------- ---------- ---------L KKGFGKCRLN ---------- ----------
AtM25/1-349   ---------- ---------- ---------- ---------- ---------- ---------- ---------- ---------- --LVNNAIIF EAAMVKVSVT NIVSS-----
AtM26a/1-1055 TMDDSSSTCS NDSIQSGVAN GSYKGNV--- ---------LN CQSQKWFSNG KIQPGK-VSD SNSLASEKEH QPSRL----A SDPKNQSHSS DIRRVGEAD- IVISHIQKPE
AtM26b/1-1055 TMDDSSSTCS NDSIQSGVAN GSYKGNV--- ---------LN CQSQKWFSNG KIQPGK-VSD SNSLASEKEH QPSRL----A SDPKNQSHSS DIRRVGEAD- IVISHIQKPE
AtM27a/1-1116 RPLTPQEELQ PVGQIREASN KANTAELKLF LEVEH-LDLR PIPPPEKSKE DILLFFKLYD PEKAVLSYAG RLMVKSSSKP MDITGKLNEM VGFAPDEEIE LFEEIKFEPC
AtM27b/1-1115 RPLTPQEELQ PVGQIREASN KANTAELKLF LEVEH-LDLR PIPPPEKSKE DILLFFKLYD PEKAVLSYAG RLMVKSSSKP MDITGKLNEM VGFAPDEEIE LFEEIKFEPC
AtM27c/1-985  RPLTPQEELQ PVGQIREASN KANTAELKLF LEVEH-LDLR PIPPPEKSKE DILLFFKLYD PEKAVLSYAG RLMVKSSSKP MDITGKLNEM VGFAPDEEIE LFEEIKFEPC
AtM28/1-369   ---------- ---------- ---------- ---------- ---------- ---------- ---------- ---------- ---------- ---------- ----------
OsM1a/1-1277  -RSRSTSNMN ITEDVDDLPS STTSSDR-NT SGCGPAPKLD QETVLLTLKD RLRKLGQRLH EKEIEGRKLL QAHLEKKAAA ESATGSSSSL SSNSLEETPE VLKSPDQSSV
OsM1b/1-1252  -RSRSTSNMN ITEDVDDLPS STTSSDR-NT SGCGPAPKLD QETVLLTLKD RLRKLGQRLH EKEIEGRKLL QAHLEKKAAA ESATGSSSSL SSNSLEETPE VLKSPDQSSV
OsM1c/1-1249  -RSRSTSNMN ITEDVDDLPS STTSSDR-NT SGCGPAPKLD QETVLLTLKD RLRKLGQRLH EKEIEGRKLL QAHLEKKAAA ESATGSSSSL SSNSLEETPE VLKSPDQSSV
OsM2/1-1110   RPLTLQEEAS SIGHLTDPSN KSLNSELKLF LEVALGQENH PISVPPKTKD DILIFFKLYD PEKEELRYVG KLLVKASGKP ADIVQKLQEM AGFQSDEDIE LYEEVMFEPS
OsM3/1-235    ---------- ---------- ---------- ---------- ---------- ---------- ---------- ---------- ---------- ---------- ----------
OsM4/1-1261   QRNKGISIIS FSEEGEGIPS SSTGGSARCS SSCGTSAKLD QDTVLLTLKD KLRKLGQRLH EKNMEGRKLL QAHFE----A MEAKTSGSSP SSSPLDETPD VVKSPEQSAE
OsM5/1-998    RPLTPQEETH TVGQLKEAAN KAHNAELKLF LEVELGLDLK PLPLPDKTRE DILLFFKLYD PEKEQLRYVG RLFVKASGKP QDILPKLRKM AGFSQDEEIE LYEEIKFEPN
OsM6/1-222    ---------- ---------- ---------- ---------- ---------- ---------- ---------- ---------- ---------- ---------- ----------
OsM7/1-213    ---------- ---------- ---------- ---------- ---------- ---------- ---------- ---------- ---------- ---------- ----------
OsM8/1-223    ---------- ---------- ---------- ---------- ---------- ---------- ---------- ---------- ---------- ---------- ----------
OsM9/1-169    ---------- ---------- ---------- ---------- ---------- ---------- ---------- ---------- -----GCFPL ---------- ----------
OsM10/1-188   ---------- ---------- ---------- ---------- ---------- ---------- ---------- ---------- ---------- ---------- ----------
OsM11/1-184   ---------- ---------- ---------- ---------- ---------- ---------- ---------- ---------- ---------- ---------- ----------
OsM12/1-1137  DSSSTCSSDS VPSVILNTGG AWTNVKSSSN RGNNRRNNKD HDARSGLGHG GLNSVPNGIA GSSSSTSVNS KDHQHEDDRV VSQKKQNAHR QTDVMSPSKL RMTESSVSPI
OsM13/1-1125  RPLGPHEESQ SVGQLREVSN KAHNAELKLF LEVETGVDLR PIRPPEKSKE DILLFFKLYN PEKEELCFVG RLFVKATGKP SEILTKLNEM AGFAPNEEIE LYEEIKFEPN

             991
AtM1a/1-1074  GSRNRIPVG- --R---ERKT VQSIVNSVDM DRPKEKSTAV LS-------- ---------- ---------- ---------- --------SP RNVAKNPSPL TQTK------
AtM1b/1-997   GSRNRIPVGR ------ERKT VQSIVNSVDM DRPKEKSTAV LS-------- ---------- ---------- ---------- --------SP RNVAKNPSPL TQTKPEKKSI
AtM1c/1-1082  GSRNRIPVG- --R---ERKT VQSIVNSVDM DRPKEKSTAV LS-------- ---------- ---------- ---------- --------SP RNVAKNPSPL TQTK------
AtM1d/1-1055  GSRNRIPVG- --R---ERKT VQSIVNSVDM DRPKEKSTAV LS-------- ---------- ---------- ---------- --------SP RNVAKNPSPL TQTK------
AtM2/1-227    ---------- ---------- ---------- ---------- ---------- ---------- ---------- ---------- ---------- ---------- ----------
AtM3/1-268    ---------- ---------- ---------- ---------- ---------- ---------- ---------- ---------- ---------- ---------- ----------
AtM4/1-231    ---------- ---------- ---------- ---------- ---------- ---------- ---------- ---------- ---------- ---------- ----------
AtM5/1-228    F--------- ---------- ---------- ---------- ---------- ---------- ---------- ---------- ---------- ---------- ----------
AtM6/1-269    ---------- ---------- ---------- ---------- ---------- ---------- ---------- ---------- ---------- ---------- ----------
AtM7a/1-442   SFSDIE---- ---------- ---------- ---------- ---------- ---------- ---------- ---------- ---------- ---------- ----------
AtM7b/1-299   ---------- ---------- ---------- ---------- ---------- ---------- ---------- ---------- ---------- ---------- ----------
AtM8/1-297    NEDNVF---- ---------- ---------- ---------- ---------- ---------- ---------- ---------- ---------- ---------- ----------
AtM9/1-265    SLYDDIDNNV ---------- ---------- ---------- ---------- ---------- ---------- ---------- ---------- ---------- ----------
AtM10a/1-1115 VMCEQIDKKT SFR------- ---------- ---------- ---------- ---------- ---------- ---------- ---------- ---------- ----------
AtM10b/1-1114 VMCEQIDKKT SFR------- ---------- ---------- ---------- ---------- ---------- ---------- ---------- ---------- ----------
AtM11/1-330   TLDDVI---- ---------- ---------- ---------- ---------- ---------- ---------- ---------- ---------- ---------- ----------
AtM12a/1-282  ---------- ---------- ---------- ---------- ---------- ---------- ---------- ---------- ---------- ---------- ----------
AtM12b/1-264  ---------- ---------- ---------- ---------- ---------- ---------- ---------- ---------- ---------- ---------- ----------
AtM13a/1-351  ---------- ---------- ---------- ---------- ---------- ---------- ---------- ---------- ---------- ---------- ----------
AtM13b/1-453  TLDDFV---- ---------- ---------- ---------- ---------- ---------- ---------- ---------- ---------- ---------- ----------
AtM14/1-317   SDDGVFDDFF ---------- ---------- ---------- ---------- ---------- ---------- ---------- ---------- ---------- ----------
AtM15/1-324   TLDDVV---- ---------- ---------- ---------- ---------- ---------- ---------- ---------- ---------- ---------- ----------
AtM16/1-298   SFNDVF---- ---------- ---------- ---------- ---------- ---------- ---------- ---------- ---------- ---------- ----------
AtM17/1-601   SFNDGV---- ---------- ---------- ---------- ---------- ---------- ---------- ---------- ---------- ---------- ----------
AtM18/1-319   TLDDVL---- ---------- ---------- ---------- ---------- ---------- ---------- ---------- ---------- ---------- ----------
AtM19/1-328   SFEDVV---- ---------- ---------- ---------- ---------- ---------- ---------- ---------- ---------- ---------- ----------
AtM20/1-306   RLDDVV---- ---------- ---------- ---------- ---------- ---------- ---------- ---------- ---------- ---------- ----------
AtM21/1-301   SFDDIV---- ---------- ---------- ---------- ---------- ---------- ---------- ---------- ---------- ---------- ----------
AtM22/1-564   TLDDVVS--- ---------- ---------- ---------- ---------- ---------- ---------- ---------- ---------- ---------- ----------
AtM23/1-382   ---------- ---------- ---------- ---------- ---------- ---------- ---------- ---------- ---------- ---------- ----------
AtM24a/1-397  ---------- ---------- ---------- ---------- ---------- ---------- ---------- ---------- ---------- ---------- ----------
AtM24b/1-346  ---------- ---------- ---------- ---------- ---------- ---------- ---------- ---------- ---------- ---------- ----------
AtM25/1-349   ---------- ---------- ---------- ---------- ---------- ---------- ---------- ---------- ---------- ---------- ----------
AtM26a/1-1055 SPKERSPVS- --K---DPNM IQM------- ---KEKSAAV LS-------- ---------- ---------- ---------- --------PS RAAPWNPPSP VQAK------
AtM26b/1-1055 SPKERSPVS- --K---DPNM IQM------- ---KEKSAAV LS-------- ---------- ---------- ---------- --------PS RAAPWNPPSP VQAK------
AtM27a/1-1116 VMCEHLDKKT SFR------- ---------- ---------- ---------- ---------- ---------- ---------- ---------- ---------- ----------
AtM27b/1-1115 VMCEHLDKKT SFR------- ---------- ---------- ---------- ---------- ---------- ---------- ---------- ---------- ----------
AtM27c/1-985  VMCEHLDKKT SFR------- ---------- ---------- ---------- ---------- ---------- ---------- ---------- ---------- ----------
AtM28/1-369   ---------- ---------- ---------- ---------- ---------- ---------- ---------- ---------- ---------- ---------- ----------
OsM1a/1-1277  TISDADINAS PSKFGASKEV TPVTPTTILS TEPVPTVAST LSKDEPVLCE DHVSCSTPQI DTPITSNPPQ VVKTVTLPSG MLLVGH--AI QAPSRSPAPQ VDRVSKAIAA
OsM1b/1-1252  TISDADINAS PSKFGASKEV TPVTPTTILS TEPVPTVAST LSKDEPVLCE DHVSCSTPQI DTPITSNPPQ VVKTVTLPSG MLLVGH--AI QAPSRSPAPQ VDRVSKAIAA
OsM1c/1-1249  TISDADINAS PSKFGASKEV TPVTPTTILS TEPVPTVAST LSKDEPVLCE DHVSCSTPQI DTPITSNPPQ VVKTVTLPSG MLLVGH--AI QAPSRSPAPQ VDRVSKAIAA
OsM2/1-1110   VMCEPI---- ---------- ---------- ---------- ---------- ---------- ---------- ---------- ---------- ---------- ----------
OsM3/1-235    ---------- ---------- ---------- ---------- ---------- ---------- ---------- ---------- ---------- ---------- ----------
OsM4/1-1261   GTTDAKANGT PNK---DEPV TNCVAEESVS VMPGTKSTEA LSGMALAKTK VEPVSNKDHV PKPTLQANRA SANCSKSTPV DMEKDVPLPS RSPQINKPAP VPPKSPQVGN
OsM5/1-998    VMCEYIDNRL LFR------- ---------- ---------- ---------- ---------- ---------- ---------- ---------- ---------- ----------
OsM6/1-222    ---------- ---------- ---------- ---------- ---------- ---------- ---------- ---------- ---------- ---------- ----------
OsM7/1-213    ---------- ---------- ---------- ---------- ---------- ---------- ---------- ---------- ---------- ---------- ----------
OsM8/1-223    ---------- ---------- ---------- ---------- ---------- ---------- ---------- ---------- ---------- ---------- ----------
OsM9/1-169    ---------- ---------- ---------- ---------- ---------- ---------- ---------- ---------- ---------- ---------- ----------
OsM10/1-188   ---------- ---------- ---------- ---------- ---------- ---------- ---------- ---------- ---------- ---------- ----------
OsM11/1-184   ---------- ---------- ---------- ---------- ---------- ---------- ---------- ---------- ---------- ---------- ----------
OsM12/1-1137  KKQHIFSQQP KSSLESANNL SSRASEASGA VTASSVMGVT TTPTAQLV-- ---------- ---------- ---------- ---------- ---------- ----------
OsM13/1-1125  VMCEHIDKKL TFR------- ---------- ---------- ---------- ---------- ---------- ---------- ---------- ---------- ----------
```

```
                 1101
AtM1a/1-1074     ---------- --------PE KKSISTADGI ---------- --PNRKVLAT GPPSS----- ----SQVVLP SDIQSQTVGL RADMQKLSAP K--QPPA--- --TTISRPSS
AtM1b/1-997      STADGIPNRK VLATGPPSS- ---------- ---------- ---------- ---------- ----SQVVLP SDIQSQTVGL RADMQKLSAP KQPPA----- --TTISRPSS
AtM1c/1-1082     ---------- --------PE KKSISTADGI ---------- --PNRKVLAT GPPSS----- ----SQVVLP SDIQSQTVGL RADMQKLSAP K--QPPA--- --TTISRPSS
AtM1d/1-1055     ---------- --------PE KKSISTADGI ---------- --PNRKVLAT GPPSS----- ----SQVVLP SDIQSQTVGL RADMQKLSAP K--QPPA--- --TTISRPSS
AtM2/1-227       ---------- ---------- ---------- ---------- ---------- ---------- ---------- ---------- ---------- ---------- ----------
AtM3/1-268       ---------- ---------- ---------- ---------- ---------- ---------- ---------- ---------- ---------- ---------- ----------
AtM4/1-231       ---------- ---------- ---------- ---------- ---------- ---------- ---------- ---------- ---------- ---------- ----------
AtM5/1-228       ---------- ---------- ---------- ---------- ---------- ---------- ---------- ---------- ---------- ---------- ----------
AtM6/1-269       ---------- ---------- ---------- ---------- ---------- ---------- ---------- ---------- ---------- ---------- ----------
AtM7a/1-442      ---------- ---------- ---------- ---------- ---------- ---------- ---------- ---------- ---------- ---------- ----------
AtM7b/1-299      ---------- ---------- ---------- ---------- ---------- ---------- ---------- ---------- ---------- ---------- ----------
AtM8/1-297       ---------- ---------- ---------- ---------- ---------- ---------- ---------- ---------- ---------- ---------- ----------
AtM9/1-265       ---------- ---------- ---------- ---------- ---------- ---------- ---------- ---------- ---------- ---------- ----------
AtM10a/1-1115    ---------- ---------- ---------- ---------- ---------- ---------- ---------- ---------- ---------- -----LCQIE DGDIICYQKP
AtM10b/1-1114    ---------- ---------- ---------- ---------- ---------- ---------- ---------- ---------- ---------- -----LCQIE DGDIICYQKP
AtM11/1-330      ---------- ---------- ---------- ---------- ---------- ---------- ---------- ---------- ---------- ---------- ----------
AtM12a/1-282     ---------- ---------- ---------- ---------- ---------- ---------- ---------- ---------- ---------- ---------- ----------
AtM12b/1-264     ---------- ---------- ---------- ---------- ---------- ---------- ---------- ---------- ---------- ---------- ----------
AtM13a/1-351     ---------- ---------- ---------- ---------- ---------- ---------- ---------- ---------- ---------- ---------- ----------
AtM13b/1-453     ---------- ---------- ---------- ---------- ---------- ---------- ---------- ---------- ---------- ---------- ----------
AtM14/1-317      ---------- ---------- ---------- ---------- ---------- ---------- ---------- ---------- ---------- ---------- ----------
AtM15/1-324      ---------- ---------- ---------- ---------- ---------- ---------- ---------- ---------- ---------- ---------- ----------
AtM16/1-298      ---------- ---------- ---------- ---------- ---------- ---------- ---------- ---------- ---------- ---------- ----------
AtM17/1-601      ---------- ---------- ---------- ---------- ---------- ---------- ---------- ---------- ---------- ---------- ----------
AtM18/1-319      ---------- ---------- ---------- ---------- ---------- ---------- ---------- ---------- ---------- ---------- ----------
AtM19/1-328      ---------- ---------- ---------- ---------- ---------- ---------- ---------- ---------- ---------- ---------- ----------
AtM20/1-306      ---------- ---------- ---------- ---------- ---------- ---------- ---------- ---------- ---------- ---------- ----------
AtM21/1-301      ---------- ---------- ---------- ---------- ---------- ---------- ---------- ---------- ---------- ---------- ----------
AtM22/1-564      ---------- ---------- ---------- ---------- ---------- ---------- ---------- ---------- ---------- ---------- ----------
AtM23/1-382      ---------- ---------- ---------- ---------- ---------- ---------- ---------- ---------- ---------- ---------- ----------
AtM24a/1-397     ---------- ---------- ---------- ---------- ---------- ---------- ---------- ---------- ---------- ---------- ----------
AtM24b/1-346     ---------- ---------- ---------- ---------- ---------- ---------- ---------- ---------- ---------- ---------- ----------
AtM25/1-349      ---------- ---------- ---------- ---------- ---------- ---------- ---------- ---------- ---------- ---------- ----------
AtM26a/1-1055    ---------- --------PE KKGVSNVEAV ---------- --PNRKVISV KSPSS----- ----HHASPS REAQLQTVGP RADIQKIASP KPVEQPA--- --PPMSRPLS
AtM26b/1-1055    ---------- --------PE KKGVSNVEAV ---------- --PNRKVISV KSPSS----- ----HHASPS REAQLQTVGP RADIQKIASP KPVEQPA--- --PPMSRPLS
AtM27a/1-1116    ---------- ---------- ---------- ---------- ---------- ---------- ---------- ---------- ---------- -----LCQIE DGDIICFQKP
AtM27b/1-1115    ---------- ---------- ---------- ---------- ---------- ---------- ---------- ---------- ---------- -----LCQIE DGDIICFQKP
AtM27c/1-985     ---------- ---------- ---------- ---------- ---------- ---------- ---------- ---------- ---------- -----LCQIE DGDIICFQKP
AtM28/1-369      ---------- ---------- ---------- ---------- ---------- ---------- ---------- ---------- ---------- ---------- ----------
OsM1a/1-1277     PTKSPAPQVD KVSIAVPTPS KSPATQGEKV AKAILVPPKS LAPQVGKVAK TIPTP-KQPA PLVDKVTSLD PVSKQMSSMS NSEAREAILP KKAAVLSVSQ T-PAISRPSS
OsM1b/1-1252     PTKSPAPQVD KVSIAVPTPS KSPATQGEKV AKAILVPPKS LAPQVGKVAK TIPTP-KQPA PLVDKVTSLD PVSKQMSSMS NSEAREAILP KKAAVLSVSQ T-PAISRPSS
OsM1c/1-1249     PTKSPAPQVD KVSIAVPTPS KSPATQGEKV AKAILVPPKS LAPQVGKVAK TIPTP-KQPA PLVDKVTSLD PVSKQMSSMS NSEAREAILP KKAAVLSVSQ T-PAISRPSS
OsM2/1-1110      ---------- ---------- ---------- ---------- ---------- ---------- ---------- ---------- -------NIN DSFLSSQLED GDIICYQKRC
OsM3/1-235       ---------- ---------- ---------- ---------- ---------- ---------- ---------- ---------- ---------- ---------- ----------
OsM4/1-1261      ATPVPPKSPP IEKACPVPPK SPPSAKDTSL ---------- --PSVRSLQI DKPVPVPPRL PQVDKAASLS SELPQTSTTS NSEAQEETAA IRVASPSVSE VTVTASRPSS
OsM5/1-998       ---------- ---------- ---------- ---------- ---------- ---------- ---------- ---------- ---------- ----ACQLED GDIVCFQKSP
OsM6/1-222       ---------- ---------- ---------- ---------- ---------- ---------- ---------- ---------- ---------- ---------- ----------
OsM7/1-213       ---------- ---------- ---------- ---------- ---------- ---------- ---------- ---------- ---------- ---------- ----------
OsM8/1-223       ---------- ---------- ---------- ---------- ---------- ---------- ---------- ---------- ---------- ---------- ----------
OsM9/1-169       ---------- ---------- ---------- ---------- ---------- ---------- ---------- ---------- ---------- ---------- ----------
OsM10/1-188      ---------- ---------- ---------- ---------- ---------- ---------- ---------- ---------- ---------- ---------- ----------
OsM11/1-184      ---------- ---------- ---------- ---------- ---------- ---------- ---------- ---------- ---------- ---------- ----------
OsM12/1-1137     ---------- ---------- ---------- ---------- ---------- ------SNKG PLTIQATHNE RSVPVARHPQ VPTVSKSEAQ KQTSLVSSGT ATQAITVSRP
OsM13/1-1125     ---------- ---------- ---------- ---------- ---------- ---------- ---------- ---------- ---------- ----SSQLED GDIICFQKSP

                 1211
AtM1a/1-1074     APIIPAMRPS PITVSSSVQT TTSLPRSVSS AGRLGPDPSL HNQQTYTPQS YKNAIVGNSL GSSSSSFNHH PSSHGVVPTT LPSSSYSQAP TSSYQSSFPY -------SQD
AtM1b/1-997      APIIPAMRPS PITVSSSVQT TTSLPRSVSS AGRLGPDPSL HNQQTYTPQS YKNAIVGNSL GSSSSSFNHH PSSHGVVPTT LPSSSYSQAP TSSYQSSFPY -------SQD
AtM1c/1-1082     APIIPAMRPS PITVSSSVQT TTSLPRSVSS AGRLGPDPSL HNQQTYTPQS YKNAIVGNSL GSSSSSFNHH PSSHGVVPTT LPSSSYSQAP TSSYQSSFPY -------SQD
AtM1d/1-1055     APIIPAMRPS PITVSSSVQT TTSLPRSVSS AGRLGPDPSL HNQQTYTPQS YKNAIVGNSL GSSSSSFNHH PSSHGVVPTT LPSSSYSQAP TSSYQSSFPY -------SQD
AtM2/1-227       ---------- ---------- ---------- ---------- ---------- ---------- ---------- ---------- ---------- ---------- ----------
AtM3/1-268       ---------- ---------- ---------- ---------- ---------- ---------- ---------- ---------- ---------- ---------- ----------
AtM4/1-231       ---------- ---------- ---------- ---------- ---------- ---------- ---------- ---------- ---------- ---------- ----------
AtM5/1-228       ---------- ---------- ---------- ---------- ---------- ---------- ---------- ---------- ---------- ---------- ----------
AtM6/1-269       ---------- ---------- ---------- ---------- ---------- ---------- ---------- ---------- ---------- ---------- ----------
AtM7a/1-442      ---------- ---------- ---------- -----LSKAN STLRELTEAG FKLDWLKKKL EEVSLKRKNA VDDGSRVKQV EERIKILKVD IVGTQR---- ----------
AtM7b/1-299      ---------- ---------- ---------- ---------- ---------- ---------- ---------- ---------- ---------- ---------- ----------
AtM8/1-297       ---------- ---------- ---------- ---------- ---------- ---------- ---------- ---------- ---------- ---------- ----------
AtM9/1-265       ---------- ---------- ---------- ---------- ---------- ---------- ---------- ---------- ---------- ---------- ----------
AtM10a/1-1115    LSIEESEFRY PDVPSFLEYV QNRELVRFRT LEKPKEDEFT MELSKLHTYD DVVERVAEKL GLDDPSKLRL TSHNCYSQQP KPQPIKYRGV DHLSDMLVHY NQTSDILYYE
AtM10b/1-1114    LSIEESEFRY PDVPSFLEYV QNRELVRFRT LEKPKEDEFT MELSKLHTYD DVVERVAEKL GLDDPSKLRL TSHNCYSQQP KPQPIKYRGV DHLSDMLVHY NQTSDILYYE
AtM11/1-330      ---------- ---------- ---------- ---------- ---------- ---------- ---------- ---------- ---------- ---------- ----------
AtM12a/1-282     ---------- ---------- ---------- ---------- ---------- ---------- ---------- ---------- ---------- ---------- ----------
AtM12b/1-264     ---------- ---------- ---------- ---------- ---------- ---------- ---------- ---------- ---------- ---------- ----------
AtM13a/1-351     ---------- ---------- ---------- ---------- ---------- ---------- ---------- ---------- ---------- ---------- ----------
AtM13b/1-453     ---------- ---------- ---------- ---------- ---------- ---------- ---------- ---------- ---------- ---------- ----------
AtM14/1-317      ---------- ---------- ---------- ---------- ---------- ---------- ---------- ---------- ---------- ---------- ----------
AtM15/1-324      ---------- ---------- ---------- ---------- ---------- ---------- ---------- ---------- ---------- ---------- ----------
AtM16/1-298      ---------- ---------- ---------- ---------- ---------- ---------- ---------- ---------- ---------- ---------- ----------
AtM17/1-601      ---------- ---------- ---------- ---------- ---------- ---------- -------CRF SGFCGFVGES LFSYAWKQGP SLYSLSFMSN GTE-------
AtM18/1-319      ---------- ---------- ---------- ---------- ---------- ---------- ---------- ---------- ---------- ---------- ----------
AtM19/1-328      ---------- ---------- ---------- ---------- ---------- ---------- ---------- ---------- ---------- ---------- ----------
AtM20/1-306      ---------- ---------- ---------- ---------- ---------- ---------- ---------- ---------- ---------- ---------- ----------
AtM21/1-301      ---------- ---------- ---------- ---------- ---------- ---------- ---------- ---------- ---------- ---------- ----------
AtM22/1-564      ---------- ---------- ---------- ---------- ---------- ---------- ---------- ---------- ---------- ---------- ----------
AtM23/1-382      ---------- ---------- ---------- ---------- ---------- ---------- ---------- ---------- ---------- ---------- ----------
AtM24a/1-397     ---------- ---------- ---------- ---------- ---------- ---------- ---------- ---------- ---------- ---------- ----------
AtM24b/1-346     ---------- ---------- ---------- ---------- ---------- ---------- ---------- ---------- ---------- ---------- ----------
AtM25/1-349      ---------- ---------- ---------- ---------- ---------- ---------- ---------- ---------- ---------- ---------- ----------
AtM26a/1-1055    APIIPPTQAA PV-ISAVQTS TASLARSMSS TGRLG---SP THSQAYNPQS YKHAIVG--- ----SSGFTH PSSQSSGTST LPPYSHPSPI SVSNQSGFPI NVGSWDVSSG
AtM26b/1-1055    APIIPPTQAA PV-ISAVQTS TASLARSMSS TGRLG---SP THSQAYNPQS YKHAIVG--- ----SSGFTH PSSQSSGTST LPPYSHPSPI SVSNQSGFPI NVGSWDVSSG
AtM27a/1-1116    LVNKEIECLY PAVPSFLEYV QNRQLVRFRA LEKPKEDEFV LELSKQHTYD DVVEKVAEKL GLDDPSKLRL TSHNCYSQQP KPQPIKYRGV DHLSDMLVHY NQTSDILYYE
AtM27b/1-1115    LVNKEIECLY PAVPSFLEYV QNRQLVRFRA LEKPKEDEFV LELSKQHTYD DVVEKVAEKL GLDDPSKLRL TSHNCYSQQP KPQPIKYRGV DHLSDMLVHY NQTSDILYYE
AtM27c/1-985     LVNKEIECLY PAVPSFLEYV QNRQLVRFRA LEKPKEDEFV LELSKQHTYD DVVEKVAEKL GLDDPSKLRL TSHNCYSQQP KPQPIKYRGV DHLSDMLVHY NQTSDILYYE
AtM28/1-369      ---------- ---------- ---------- ---------- ---------- ---------- ---------- ---------- ---------- ---------- ----------
OsM1a/1-1277     APLFQVPRST LP-PTPAVQV PPMLSRSMTL AGR-SRNEPS PSVPSYTAQT YRNAIIGKSN LDTASASLDH --STSFGQNV ALSQPLSSYA SAASAMVPPV GRNGQLPGKQ
OsM1b/1-1252     APLFQVPRST LP-PTPAVQV PPMLSRSMTL AGR-SRNEPS PSVPSYTAQT YRNAIIGKSN LDTASASLDH --STSFGQNV ALSQPLSSYA SAASAMVPPV GRNGQLPGKQ
OsM1c/1-1249     APLFQVPRST LP-PTPAVQV PPMLSRSMTL AGR-SRNEPS PSVPSYTAQT YRNAIIGKSN LDTASASLDH --STSFGQNV ALSQPLSSYA SAASAMVPPV GRNGQLPGKQ
OsM2/1-1110      SPEKLDHYRC ADVPSFFEYI QNRQVVHFRL LENPKDDDFT LELSKRFTYD DVVEKVANQL GLDDPSKLRL TQHLPYSQMP KSHYIKYRGL DHLSDMLRNG NQMSDILYYE
OsM3/1-235       ---------- ---------- ---------- ---------- ---------- ---------- ---------- ---------- ---------- ---------- ----------
OsM4/1-1261      APVFPAPRST V--PATQVQV STLLSRSMSE ATRRSGNDPS PSAPAYIPQT YRNAIIG-KH GRGTTSGTTA YQSTSLGQGT ALSQPLSTYA PTMSVTMPPA GRNDQFSGRH
OsM5/1-998       KPDTADQYRY PDVPSFLVYI RNRQVVHFRS LEKPKEDDFC LEMSKAFTYD EVVEKVAQKL GVDDPTKIRL TSHNCYSQQP KPQPIKYRGV ERLLDMLIHY NQTSDILYYE
OsM6/1-222       ---------- ---------- ---------- ---------- ---------- ---------- ---------- ---------- ---------- ---------- ----------
OsM7/1-213       ---------- ---------- ---------- ---------- ---------- ---------- ---------- ---------- ---------- ---------- ----------
OsM8/1-223       ---------- ---------- ---------- ---------- ---------- ---------- ---------- ---------- ---------- ---------- ----------
OsM9/1-169       ---------- ---------- ---------- ---------- ---------- ---------- ---------- ---------- ---------- ---------- ----------
OsM10/1-188      ---------- ---------- ---------- ---------- ---------- ---------- ---------- ---------- ---------- ---------- ----------
OsM11/1-184      ---------- ---------- ---------- ---------- ---------- ---------- ---------- ---------- ---------- ---------- ----------
OsM12/1-1137     LSAPQVPAAK QTAPVASAVQ TVPLLSHSMS AVGRLGNEPS ASAPSYIPRS YRNAMMERSS VGASSFTHQT SSEQRVAQSQ SMFSLSPSIL SPEHLIG--- --NDRSSLRQ
OsM13/1-1125     VSDGETQVRY PDVPSFLEYV HNRQVVHFRS LEKPKEDDFC LELSKLHTYD DVVERVARQL GLDDPSKIRL TSHNCYSQQP KPQPIRYRGV EHLLDMLVHY NQTSDILYYE
```

```
             1321
AtM1a/1-1074  GLLWTGRSPS SVNM-----G MY-------- ---------N NTYSPAVTSN RSL------- ---------- -------NHM DVQIAQQQAQ SMMTDEFPHL DIINDLLEDE
AtM1b/1-997   GLLWTGRSPS SVNM------ ---------- ---------- ---------- ---------- GMYNNTYSPA VTSNRSLNHM DVQIAQQQAQ SMMTDEFPHL DIINDLLEDE
AtM1c/1-1082  GLLWTGRSPS SVNM-----G MY-------- ---------N NTYSPAVTSN RSL------- ---------- -------NHM DVQIAQQQAQ SMMTDEFPHL DIINDLLEDE
AtM1d/1-1055  GLLWTGRSPS SVNM-----G MY-------- ---------N NTYSPAVTSN RSL------- ---------- -------NHM DVQIAQQQAQ SMMTDEFPHL DIINDLLEDE
AtM2/1-227    ---------- ---------- ---------- ---------- ---------- ---------- ---------- ---------- ---------- ---------- ----------
AtM3/1-268    ---------- ---------- ---------- ---------- ---------- ---------- ---------- ---------- ---------- ---------- ----------
AtM4/1-231    ---------- ---------- ---------- ---------- ---------- ---------- ---------- ---------- ---------- ---------- ----------
AtM5/1-228    ---------- ---------- ---------- ---------- ---------- ---------- ---------- ---------- ---------- ---------- ----------
AtM6/1-269    ---------- ---------- ---------- ---------- ---------- ---------- ---------- ---------- ---------- ---------- ----------
AtM7a/1-442   ---------- ---------- ---------- ---------- ---------- ---------- ---------- ---------- ---------- ---------- ----------
AtM7b/1-299   ---------- ---------- ---------- ---------- ---------- ---------- ---------- ---------- ---------- ---------- ----------
AtM8/1-297    ---------- ---------- ---------- ---------- ---------- ---------- ---------- ---------- ---------- ---------- ----------
AtM9/1-265    ---------- ---------- ---------- ---------- ---------- ---------- ---------- ---------- ---------- ---------- ----------
AtM10a/1-1115 VLDIPLPELQ GLKTLKVAFH SATKDEVIIH NIRLPKQSTV GDVINE--LK TKVELSHQDA ELRLLEVFFH KIYKIFPSTE RIENINDQYW TLRAEEIPEE EKNIGPNDRL
AtM10b/1-1114 VLDIPLPELQ GLKTLKVAFH SATKDEVIIH NIRLPKQSTV GDVINE--LK TKVELSHQDA ELRLLEVFFH KIYKIFPSTE RIENINDQYW TLRAEEIPEE EKNIGPNDRL
AtM11/1-330   ---------- ---------- ---------- ---------- ---------- ---------- ---------- ---------- ---------- ---------- ----------
AtM12a/1-282  ---------- ---------- ---------- ---------- ---------- ---------- ---------- ---------- ---------- ---------- ----------
AtM12b/1-264  ---------- ---------- ---------- ---------- ---------- ---------- ---------- ---------- ---------- ---------- ----------
AtM13a/1-351  ---------- ---------- ---------- ---------- ---------- ---------- ---------- ---------- ---------- ---------- ----------
AtM13b/1-453  ---------- ---------- ---------- ---------- ---------- ---------- ---------- ---------- ---------- ---------- ----------
AtM14/1-317   ---------- ---------- ---------- ---------- ---------- ---------- ---------- ---------- ---------- ---------- ----------
AtM15/1-324   ---------- ---------- ---------- ---------- ---------- ---------- ---------- ---------- ---------- ---------- ----------
AtM16/1-298   ---------- ---------- ---------- ---------- ---------- ---------- ---------- ---------- ---------- ---------- ----------
AtM17/1-601   ---------- ---------- ---------- ---------- ---------- ---------- ---------- ---------- ---------- ---------- ----------
AtM18/1-319   ---------- ---------- ---------- ---------- ---------- ---------- ---------- ---------- ---------- ---------- ----------
AtM19/1-328   ---------- ---------- ---------- ---------- ---------- ---------- ---------- ---------- ---------- ---------- ----------
AtM20/1-306   ---------- ---------- ---------- ---------- ---------- ---------- ---------- ---------- ---------- ---------- ----------
AtM21/1-301   ---------- ---------- ---------- ---------- ---------- ---------- ---------- ---------- ---------- ---------- ----------
AtM22/1-564   ---------- ---------- ---------- ---------- ---------- ---------- ---------- ---------- ---------- ---------- ----------
AtM23/1-382   ---------- ---------- ---------- ---------- ---------- ---------- ---------- ---------- ---------- ---------- ----------
AtM24a/1-397  ---------- ---------- ---------- ---------- ---------- ---------- ---------- ---------- ---------- ---------- ----------
AtM24b/1-346  ---------- ---------- ---------- ---------- ---------- ---------- ---------- ---------- ---------- ---------- ----------
AtM25/1-349   ---------- ---------- ---------- ---------- ---------- ---------- ---------- ---------- ---------- ---------- ----------
AtM26a/1-1055 GLLWTGGSSS TRDTTTTISG NH-------- ---------K TNTYNAPVVT TSI------- ---------- -------RPT NVQIG-RTAQ SLMTDEFPHL DIINDLLADE
AtM26b/1-1055 GLLWTGGSSS TRDTTTTISG NH-------- ---------K TNTYNAPVVT TSI------- ---------- -------RPT NVQIG-RTAQ SLMTDEFPHL DIINDLLADE
AtM27a/1-1116 VLDIPLPELQ GLKTLKVAFH HATKEEVVIH NIRLPKQSTV GDVINE--LK TKVELSHPDA ELRLLEVFYH KIYKIFPSTE RIENINDQYW TLRAEEIPEE EKNIGPNDRL
AtM27b/1-1115 VLDIPLPELQ GLKTLKVAFH HATKEEVVIH NIRLPKQSTV GDVINE--LK TKVELSHPDA ELRLLEVFYH KIYKIFPSTE RIENINDQYW TLRAEEIPEE EKNIGPNDRL
AtM27c/1-985  VLDIPLPELQ GLKTLKVAFH HATKEEVVIH NIRLPKQSTV GDVINE--LK TKVELSHPDA ELRLLEVFYH KIYKIFPSTE RIENINDQYW TLRAEEVYY- ----------
AtM28/1-369   ---------- ---------- ---------- ---------- ---------- ---------- ---------- ---------- ---------- ---------- ----------
OsM1a/1-1277  GFMFGQGKSE AIDNWNPWKG DSNANKYMWK DDSPYHQMTK GDAHTQSWRD NSYQQAGCSG TGEQGEFGGL QY-RQFQREI PTNLVSYQLP GPVGEEFPHL DIINDLLEEE
OsM1b/1-1252  GFMFGQGKSE AIDNWNPWKG DSNANKYMWK DDSPYHQMTK GDAHTQSWRD NSYQQAGCSG TGEQGEFGGL QY-RQFQREI PTNLVSYQLP GPVGEEFPHL DIINDLLEEE
OsM1c/1-1249  GFMFGQGKSE AIDNWNPWKG DSNANKYMWK DDSPYHQMTK GDAHTQSWRD NSYQQAGCSG TGEQGEFGGL QY-RQFQREI PTNLVSYQLP GPVGEEFPHL DIINDLLEEE
OsM2/1-1110   ILDIPLPVLQ DLITLRVAFY HATNNEVSSH FIRLPKGSTM SDLIED--MK SKVELSYSDA EFRLFEVYKN KIRKVYQPSE KISVNEFNGL -LCVEEVPEE EKNAGVRDRL
OsM3/1-235    ---------- ---------- ---------- ---------- ---------- ---------- ---------- ---------- ---------- ---------- ----------
OsM4/1-1261   GLESGLGKPE ARDSWQPWNA NRHVDKHLWR DDSTYQQTTN GHAYPQPWKD VNFLQARGTE TEIPSRFGGP QLPRQFQAET HADYLLQQPQ GPVAEEFPHL DIINDLLEEE
OsM5/1-998    VLDIPLPELQ ALKTLKVTYH HGTKDEVSVH SIRLPKNSTV GDVLND--IK SKVELSHPNA ELRLLEVFYH KIYKIFAPNE KIENINDQYW TLRAEEVPEE EKNLGPFDRL
OsM6/1-222    ---------- ---------- ---------- ---------- ---------- ---------- ---------- ---------- ---------- ---------- ----------
OsM7/1-213    ---------- ---------- ---------- ---------- ---------- ---------- ---------- ---------- ---------- ---------- ----------
OsM8/1-223    ---------- ---------- ---------- ---------- ---------- ---------- ---------- ---------- ---------- ---------- ----------
OsM9/1-169    ---------- ---------- ---------- ---------- ---------- ---------- ---------- ---------- ---------- ---------- ----------
OsM10/1-188   ---------- ---------- ---------- ---------- ---------- ---------- ---------- ---------- ---------- ---------- ----------
OsM11/1-184   ---------- ---------- ---------- ---------- ---------- ---------- ---------- ---------- ---------- ---------- ----------
OsM12/1-1137  GLTFGTVKPE TLNQWRE--- ---------- -----ESSQQ ASSSSSSSSN DHGAVSSSNG GEFEKFYLFG KPRSKQLSEI SARSTPFQPQ GLVSDEFPHL DIINDLLNDE
OsM13/1-1125  VLDIPLPELQ CLKTLKVAFH HATKDEVVIH SIRLPKNSTI SDVITD--LK TKVELSNPDA ELRLLEVFYH KIYKIFPPHE KIENINDQYW TLRAEEIPEE EKNLGPHDRL

             1431
AtM1a/1-1074  QCSNMVYNGS IFNPQPQVFN GQYSSYH--- ---------- -----GELLS ---------- GGRTRSFGE- EGLHYMARGP YGTDGMMPRQ WQMTNMDLSL PAMRSNGMED
AtM1b/1-997   QCSNMVYNGS IFNPQPQVFN GQYSSYH--- ---------- ---------- ------GELL SGGRTRSFGE EGLHYMARGP YGTDGMMPRQ WQMTNMDLSL PAMRSNGMED
AtM1c/1-1082  QCSNMVYNGS IFNPQPQVFN GQYSSYH--- ---------- -----GELLS ---------- GGRTRSFGE- EGLHYMARGP YGTDGMMPRQ WQMTNMDLSL PAMRSNGMED
AtM1d/1-1055  QCSNMVYNGS IFNPQPQVFN GQYSSYH--- ---------- -----GELLS ---------- GGRTRSFGE- EGLHYMARGP YGTDGMMPRQ WQMTNMDLSL PAMRSNGMED
AtM2/1-227    ---------- ---------- ---------- ---------- ---------- ---------- ---------- ---------- ---------- ---------- ----------
AtM3/1-268    ---------- ---------- ---------- ---------- ---------- ---------- ---------- ---------- ---------- ---------- ----------
AtM4/1-231    ---------- ---------- ---------- ---------- ---------- ---------- ---------- ---------- ---------- ---------- ----------
AtM5/1-228    ---------- ---------- ---------- ---------- ---------- ---------- ---------- ---------- ---------- ---------- ----------
AtM6/1-269    ---------- ---------- ---------- ---------- ---------- ---------- ---------- ---------- ---------- ---------- ----------
AtM7a/1-442   ---------- ---------- ---------- ---------- ---------- ---------- ---------- ---------- ---------- ---------- ----------
AtM7b/1-299   ---------- ---------- ---------- ---------- ---------- ---------- ---------- ---------- ---------- ---------- ----------
AtM8/1-297    ---------- ---------- ---------- ---------- ---------- ---------- ---------- ---------- ---------- ---------- ----------
AtM9/1-265    ---------- ---------- ---------- ---------- ---------- ---------- ---------- ---------- ---------- ---------- ----------
AtM10a/1-1115 IHVYHFTKEA GQNQQVQNFG EPFFLVIHEG ETLEEIKTRI QKKLHVPDED FAKWKFASFS MGRPDYLLDT DVVYN-RFQR RDVYGAWEQY LGLEHIDNAP KRAYAANQNR
AtM10b/1-1114 IHVYHFTKEA GQNQQVQNFG EPFFLVIHEG ETLEEIKTRI QKKLHVPDED FAKWKFASFS MGRPDYLLDT DVVYN-RFQR RDVYGAWEQY LGLEHIDNAP KRAYAANQNR
AtM11/1-330   ---------- ---------- ---------- ---------- ---------- ---------- ---------- ---------- ---------- ---------- ----------
AtM12a/1-282  ---------- ---------- ---------- ---------- ---------- ---------- ---------- ---------- ---------- ---------- ----------
AtM12b/1-264  ---------- ---------- ---------- ---------- ---------- ---------- ---------- ---------- ---------- ---------- ----------
AtM13a/1-351  ---------- ---------- ---------- ---------- ---------- ---------- ---------- ---------- ---------- ---------- ----------
AtM13b/1-453  ---------- ---------- ---------- ---------- ---------- ---------- ---------- ---------- ---------- ---------- ----------
AtM14/1-317   ---------- ---------- ---------- ---------- ---------- ---------- ---------- ---------- ---------- ---------- ----------
AtM15/1-324   ---------- ---------- ---------- ---------- ---------- ---------- ---------- ---------- ---------- ---------- ----------
AtM16/1-298   ---------- ---------- ---------- ---------- ---------- ---------- ---------- ---------- ---------- ---------- ----------
AtM17/1-601   ---------- ---------- ---------- ---------- ---------- ---------- ---------- ---------- ---------- ---------- ----------
AtM18/1-319   ---------- ---------- ---------- ---------- ---------- ---------- ---------- ---------- ---------- ---------- ----------
AtM19/1-328   ---------- ---------- ---------- ---------- ---------- ---------- ---------- ---------- ---------- ---------- ----------
AtM20/1-306   ---------- ---------- ---------- ---------- ---------- ---------- ---------- ---------- ---------- ---------- ----------
AtM21/1-301   ---------- ---------- ---------- ---------- ---------- ---------- ---------- ---------- ---------- ---------- ----------
AtM22/1-564   ---------- ---------- ---------- ---------- ---------- ---------- ---------- ---------- ---------- ---------- ----------
AtM23/1-382   ---------- ---------- ---------- ---------- ---------- ---------- ---------- ---------- ---------- ---------- ----------
AtM24a/1-397  ---------- ---------- ---------- ---------- ---------- ---------- ---------- ---------- ---------- ---------- ----------
AtM24b/1-346  ---------- ---------- ---------- ---------- ---------- ---------- ---------- ---------- ---------- ---------- ----------
AtM25/1-349   ---------- ---------- ---------- ---------- ---------- ---------- ---------- ---------- ---------- ---------- ----------
AtM26a/1-1055 HGT----MDN SVYRVPQQFN NQYSYHG--- ---------- -----GADLG ISSRSRSYSD DGFHQSYGE- YMPHSASSSP YG-NGQTQSQ WQMANMDFSL PAMRN---QD
AtM26b/1-1055 HGT----MDN SVYRVPQQFN NQYSYHG--- ---------- -----GADLG ISSRSRSYSD DGFHQSYGE- YMPHSASSSP YG-NGQTQSQ WQMANMDFSL PAMRN---QD
AtM27a/1-1116 ILVYHFAKET GQNQQVQNFG EPFFLVIHEG ETLEEIKNRI QKKLHVSDED FAKWKFAFMS MGRPEYLQDT DVVYN-RFQR RDVYGAFEQY LGLEHADTTP KRAYAANQNR
AtM27b/1-1115 ILVYHFAKET GQNQQVQNFG EPFFLVIHEG ETLEEIKNRI QKKLHVSDED FAKWKFAFMS MGRPEYLQDT DVVYN-RFQR RDVYGAFEQY LGLEHADTTP KRAYAANQNR
AtM27c/1-985  ---------- ---------- ---------- ---------- ---------- ---------- ---------- ---------- ---------- ---------- ----------
AtM28/1-369   ---------- ---------- ---------- ---------- ---------- ---------- ---------- ---------- ---------- ---------- ----------
OsM1a/1-1277  QSS------- --GSMAEPTL HGYHTLG--L PYSSRGNLVD SEVTSISSSG RLNLADHYYD EGYPMAYDRL NALYRLREGQ N--STLDAYS NGRMDSITSK PWLHN--FSN
OsM1b/1-1252  QSS------- --GSMAEPTL HGYHTLG--L PYSSRGNLVD SEVTSISSSG RLNLADHYYD EGYPMAYDRL NALYRLREGQ N--STLDAYS NGRMDSITSK PWLHN--FSN
OsM1c/1-1249  QSS------- --GSMAEPTL HGYHTLG--L PYSSRGNLVD SEVTSISSSG RLNLADHYYD EGYPMAYDRL NALYRLREGQ N--STLDAYS NGRMDSITSK PWLHN--FSN
OsM2/1-1110   VHVCHFIIE- --KQHIDYYG EPFFFLIRDG ETLSDIKVRI QKKLLVSDEQ FAKWKFAYIA HNRLAGEYFQ DSDIVLSRFQ KDVYGPWEQC LGLEHSDVTP KRSCLSNQNR
OsM3/1-235    ---------- ---------- ---------- ---------- ---------- ---------- ---------- ---------- ---------- ---------- ----------
OsM4/1-1261   QSN------- --GSMPESIG HDYHTFGLPL PFLLRGNLAD QEMASASSPG RFNLTEPYYD EGYSRAYDM- SAFQGTRERQ F--PSLDAYS NGLSDMSPSK PWL--NGSPN
OsM5/1-998    IHVYHFTKDT QNQTQVQNFG EPFFMVIRED ETLSSIKERI QKKLKVPDED FSKWKFAYIS LGRPDYFEDS DTVA--SRFQ RNMYGAWEQY LGLEHPDTAP RKTHNANQNR
OsM6/1-222    ---------- ---------- ---------- ---------- ---------- ---------- ---------- ---------- ---------- ---------- ----------
OsM7/1-213    ---------- ---------- ---------- ---------- ---------- ---------- ---------- ---------- ---------- ---------- ----------
OsM8/1-223    ---------- ---------- ---------- ---------- ---------- ---------- ---------- ---------- ---------- ---------- ----------
OsM9/1-169    ---------- ---------- ---------- ---------- ---------- ---------- ---------- ---------- ---------- ---------- ----------
OsM10/1-188   ---------- ---------- ---------- ---------- ---------- ---------- ---------- ---------- ---------- ---------- ----------
OsM11/1-184   ---------- ---------- ---------- ---------- ---------- ---------- ---------- ---------- ---------- ---------- ----------
OsM12/1-1137  QCE------- --RRTLKVLQ RSQQAFARQY SMPNNSSTPD YGMFAQSDPY LFDQPEQYYD EEIPRFYNSL NGTSRGLRER SYSHFDLPSY SNGQFDDMMM NQWPYGQTDL
OsM13/1-1125  IHVYHFMKDP NQNQQIQNFG DPFLLVIREG ETAAEILERI QKKLRVPDEE FSKWKLAFIS MNRPEYLQDV DVVSA-RFQR RDVYGAWEQY LGLEHTDTTP KRSYTANQNR
```

```
                  1541
AtM1a/1-1074    GTSSAANYHH SYFGLDASNP SFTSG-INGY TEFRPSNGH- --
AtM1b/1-997     GTSSAANYHH SYFGLDASNP SFTSG-INGY TEFRPSNGH- --
AtM1c/1-1082    GTSSAANYHH SYFGLDASNP SFTSG-INGY TEFRPSNGH- --
AtM1d/1-1055    GTSSAANYHH SYFGLDASNP SFTSG-INGY TEFRPSNGH- --
AtM2/1-227      ---------- ---------- ---------- ---------- --
AtM3/1-268      ---------- ---------- ---------- ---------- --
AtM4/1-231      ---------- ---------- ---------- ---------- --
AtM5/1-228      ---------- ---------- ---------- ---------- --
AtM6/1-269      ---------- ---------- ---------- ---------- --
AtM7a/1-442     ---------- ---------- ---------- ---------- --
AtM7b/1-299     ---------- ---------- ---------- ---------- --
AtM8/1-297      ---------- ---------- ---------- ---------- --
AtM9/1-265      ---------- ---------- ---------- ---------- --
AtM10a/1-1115   HAYEKPVKIY N--------- ---------- ---------- --
AtM10b/1-1114   HAYEKPVKIY N--------- ---------- ---------- --
AtM11/1-330     ---------- ---------- ---------- ---------- --
AtM12a/1-282    ---------- ---------- ---------- ---------- --
AtM12b/1-264    ---------- ---------- ---------- ---------- --
AtM13a/1-351    ---------- ---------- ---------- ---------- --
AtM13b/1-453    ---------- ---------- ---------- ---------- --
AtM14/1-317     ---------- ---------- ---------- ---------- --
AtM15/1-324     ---------- ---------- ---------- ---------- --
AtM16/1-298     ---------- ---------- ---------- ---------- --
AtM17/1-601     ---------- ---------- ---------- ---------- --
AtM18/1-319     ---------- ---------- ---------- ---------- --
AtM19/1-328     ---------- ---------- ---------- ---------- --
AtM20/1-306     ---------- ---------- ---------- ---------- --
AtM21/1-301     ---------- ---------- ---------- ---------- --
AtM22/1-564     ---------- ---------- ---------- ---------- --
AtM23/1-382     ---------- ---------- ---------- ---------- --
AtM24a/1-397    ---------- ---------- ---------- ---------- --
AtM24b/1-346    ---------- ---------- ---------- ---------- --
AtM25/1-349     ---------- ---------- ---------- ---------- --
AtM26a/1-1055   DVSASATATY SYFDLDSSNP NL-SG-INGY RDFRPSNGH- --
AtM26b/1-1055   DVSASATATY SYFDLDSSNP NL-SG-INGY RDFRPSNGH- --
AtM27a/1-1116   HAYEKPVKIY N--------- ---------- ---------- --
AtM27b/1-1115   HAYEKPVKIY N--------- ---------- ---------- --
AtM27c/1-985    ---------- ---------- ---------- ---------- --
AtM28/1-369     ---------- ---------- ---------- ---------- --
OsM1a/1-1277    PAVNLGVNPN GFSQQMGNYT NLGSGRVNGE HLYRHANGQ- -W
OsM1b/1-1252    PAVNLGVNPN GFSQQMGNYT NLGSGRVNGE HLYRHANGQ- -W
OsM1c/1-1249    PAVNLGVNPN GFSQQMGNYT NLGSGRVNGE HLYRHANGQ- -W
OsM2/1-1110     NSFDKAVKIF N--------- ---------- ---------- --
OsM3/1-235      ---------- ---------- ---------- ---------- --
OsM4/1-1261     PSMNHAVGTN GYPQQIPDYT NLASE-LNGA SLYHRRYANG RW
OsM5/1-998      HSFERPVKIY N--------- ---------- ---------- --
OsM6/1-222      ---------- ---------- ---------- ---------- --
OsM7/1-213      ---------- ---------- ---------- ---------- --
OsM8/1-223      ---------- ---------- ---------- ---------- --
OsM9/1-169      ---------- ---------- ---------- ---------- --
OsM10/1-188     ---------- ---------- ---------- ---------- --
OsM11/1-184     ---------- ---------- ---------- ---------- --
OsM12/1-1137    SLPNFGADMN GYPYQSSDYP NSANG-LSRY PPYRPANGH- --
OsM13/1-1125    HTFEKPVKIY N--------- ---------- ---------- --
```
