# Supplementary material for: MATH-Domain Family Shows Response toward Abiotic Stress in Arabidopsis and Rice
Source: Front Plant Sci. 2016 Jun 28;7:923. doi: 10.3389/fpls.2016.00923 (PMC4923191; doi:10.3389/fpls.2016.00923)
Supplement: Figure S3 — Multiple sequence alignment of full length sequences having two MATH domains in Arabidopsis and rice. The sequence analysis was performed using Seaview (version 4) multiple sequence alignment editor (Gouy et al., 2010). [file Image3.PDF]

```
              1
At2M1/1-294   ---------- ---------- ---------- ---------- ---------- ---MGASCSA PVSSTIKK--
At2M2/1-396   ---------- ---------- ---------- MYSEEKKSRN YGSIFVYCFF CFVLIVEVAR FAKPYINLQN
At2M3a/1-296  ---------- ---------- ---------- ---------- ---------- ---MGSNASD PAVLK-----
At2M3b/1-296  ---------- ---------- ---------- ---------- ---------- ---MGSNASD PAVLK-----
At2M4/1-365   ---------- ---------- ---------- ---MMCHGRK VKMRDNEMSQ IRQSLIPTGT EVSAGDGERR
At2M5/1-898   ---------- ---------- ---------- ---------- ---------- -MSINIKQTC EILPSNKSLR
At2M6a/1-420  ---------- ---------M NRGGCGGGPG RGGRGFGGRG GGPGFGPGGP GFGPGGPGFG PGGPGFGPGG
At2M6b/1-420  ---------- ---------M NRGGCGGGPG RGGRGFGGRG GGPGFGPGGP GFGPGGPGFG PGGPGFGPGG
At2M6c/1-298  ---------- ---------- ---------- ---------- ---------- ---------- ----------
At2M6d/1-298  ---------- ---------- ---------- ---------- ---------- ---------- ----------
At2M7/1-743   ---------- ---------- ---------- ---------- ---------- ---------- ----------
At2M8/1-318   ---------- ---------- ---------- ---------- ---------- MLKMGLTSFE DAIKE-----
At2M9/1-416   -------MNN SFRNKIHEQS KENLSDDRHK KSDRTLIVFN RKTPLYFYFF EQVRYITGSI YILIETPSSR
At2M10/1-411  ---------- ---------- ---------- -MEIVGHRGG WGDFPGKGVG SCVFGGGGGG PAFGGRGGGP
At2M11/1-309  ---------- ---------- ---------- ---------- ---------- -----MASSG DYDEIIK---
At2M12/1-370  ---------- ---------- --MSEKGLIK KEDHMFQEEK RKTNYGAIGL AC-FISCFFA FQFMKIVTTQ
At2M13/1-363  ---------- ---------- ---------- ---MMSYHYI NTLCIVISLL SCLFITSSFA GPVPNQENGS
At2M14/1-291  ---------- ---------- ---------- ---------- ---------- MSKYELKVTS KGSQKHEKLK
At2M15/1-379  ---------- ---------- ---------- ---MASHYRN TSAIAYLLLC LFITSATAHS FIRQITDDLK
At2M16/1-375  ---------- ---------- ---------- ---MTSLYRN TSSFVYLLFC LFITSSSAGS FIRQFSDDFN
At2M17/1-358  ---------- ---------- ---------- ---------- ---------- ---------- ----------
At2M18/1-427  ---------- ---------- ---------- -------MTL FSRVLLFLKK KSFSIAVSSL YFYICKSHFC
At2M19a/1-297 ---------- ---------- ---------- ---------- ---------- ---------- ----------
At2M19b/1-297 ---------- ---------- ---------- ---------- ---------- ---------- ----------
At2M20/1-299  ---------- ---------- ---------- ---------- ---------- ---MSRPIPI EEMVR-----
At2M21/1-300  ---------- ---------- ---------- ---------- ---------- ---MSRPISL EEMVR-----
At2M22/1-351  ---------- ---------- ---------- -----MDSHK WS--LGFTLL AFLFITSSSA ELIIKQVTQG
At2M23a/1-350 ---------- ---------- ---------- -----MNNQK WS--IGFISL AFLFITSSSA EFIIQQVTKG
At2M23b/1-327 ---------- ---------- ---------- -----MNNQK WS--IGFISL AFLFITSSSA EFIIQQVTKG
At2M24/1-333  ---------- ---------- ---------- -----MDTQK WS--IGFISL AFLFITSSSA ELLIKQRNRI
At2M25/1-352  ---------- ---------- ---------- -----MDYQK WS--LGFISL VFLLITSSSA ELLIKQVTEG
Os2M1/1-685   MTVLPLLLVC FTSIKPPLVP RASPPTSARK RRCCDCECEI IKNPIELGFL IKICTVVEDP HVTNIFFLSE

             71
At2M1/1-294   ---------- ---------- ---------- ---------- ---------- ---------- ----------
At2M2/1-396   LIETEAVVEE GFMAVGNSGN LPCGSSKPSS ASVRAHDEQK LSQAVTT--- ---------- ----------
At2M3a/1-296  ---------- ---------- ---------- ---------- ---------- ---------- ----------
At2M3b/1-296  ---------- ---------- ---------- ---------- ---------- ---------- ----------
At2M4/1-365   RCKTPEGQRE KVVSDNDFVE EDCVLSKGGE LLFGFDITQL LGQQNWQGDS TIVN------ ----------
At2M5/1-898   KRLGR----- ---------- ---------- ---------- ---------- ---------- ----------
At2M6a/1-420  PGFGGRGPRG PGFGPRGPGP WSGPRGPRPG GGGGPGPGPW SGPRGPRPGG GGGPGSGCGS GTGGGNQGQG
At2M6b/1-420  PGFGGRGPRG PGFGPRGPGP WSGPRGPRPG GGGGPGPGPW SGPRGPRPGG GGGPGSGCGS GTGGGNQGQG
At2M6c/1-298  ---------- ---------- ---------- ---------- ---------- ---------- ----------
At2M6d/1-298  ---------- ---------- ---------- ---------- ---------- ---------- ----------
At2M7/1-743   ---------- ---------- ---------- ---------- ---------- ---------- ----------
At2M8/1-318   ---------- ---------- ---------- ---------- ---------- ---------- ----------
At2M9/1-416   NLPIYSNICI PISSHPNTNY RPKKRKKNPP TVYLTTIILI MGLLSLEDTI KE-------- ----------
At2M10/1-411  GRGYGGGPRV HGPGYGIGSR GPDPGPGFFF GGAGPGPGYG GGGGHGPGYG GGGDGRGYGS ETGGGNHGPE
At2M11/1-309  ---------- ---------- ---------- ---------- ---------- ---------- ----------
At2M12/1-370  PSTTTTSSIA VIDSPMSSHK LSDRRE---- ---------- ---------- ---------- ----------
At2M13/1-363  QKIFPTQISS RDSKVSLSST VK-------- ---------- ---------- ---------- ----------
At2M14/1-291  TALLP----- ---------- ---------- ---------- ---------- ---------- ----------
At2M15/1-379  TNLQQEVGAE PIQNLDVGHY LQENKEISSR DYKVSASNAV K--------- ---------- ----------
At2M16/1-375  TIQQQKGKDG PTPNLEKGNY LHKHNEISSS LDYKVSASNI VK-------- ---------- ----------
At2M17/1-358  ---------- ---------- ---------- ---------- ---------- ---------- ----------
At2M18/1-427  LPYIYTTLKS TIFCPKKFII ME-------- ---------- ---------- ---------- ----------
At2M19a/1-297 ---------- -ME------- ---------- ---------- ---------- ---------- ----------
At2M19b/1-297 ---------- -ME------- ---------- ---------- ---------- ---------- ----------
At2M20/1-299  ---------- ---------- ---------- ---------- ---------- ---------- ----------
At2M21/1-300  ---------- ---------- ---------- ---------- ---------- ---------- ----------
At2M22/1-351  RGIEYNNSYS LTSNLGVTTR ---------- ---------- ---------- ---------- ----------
At2M23a/1-350 RGIEYNSSYS LEENLGVTR- ---------- ---------- ---------- ---------- ----------
At2M23b/1-327 RGIEYNSSYS LEENLGVTR- ---------- ---------- ---------- ---------- ----------
At2M24/1-333  QQFSQFTNGV TR-------- ---------- ---------- ---------- ---------- ----------
At2M25/1-352  RGIEYNNSYS LKTNLGLTR- ---------- ---------- ---------- ---------- ----------
Os2M1/1-685   MCNIVQVPSS ALLAEDGFHL QVIPKKTKCW ETIWSRSFIM LHRKNGFFH- ---------- ----------
```

```
             141
At2M1/1-294      ---NWRDHPP SS-------- -YSLKIHNFS QLEKTTSFSG HKYQSRLFSS G-DIT----- ---GNVK--D
At2M2/1-396      ---DTRTRPP NS-------- -YCVKFQSFV TMAKQVKENG GKYESRPFSV G-GYNWTLLI YPVIYIP--T
At2M3a/1-296     ---TWRRNPP SS-------- -TLVRLSQL- --------AN EKYESPPFSS G-AHNWRLVV HPKGNEA--D
At2M3b/1-296     ---TWRRNPP SS-------- -TLVRLSQL- --------AN EKYESPPFSS G-AHNWRLVV HPKGNEA--D
At2M4/1-365      ---NLREHPP SS-------- -YSLKINKLS QL------TF DKYESHRFLS G-GYNWRLVI YPKGNEK--D
At2M5/1-898      ---DMENHEQ TS-------- -FTFEIDNFL E-------KG DAI-SPIFIS G-GCEWFIRV WQIE------
At2M6a/1-420     G--MWRDERP SN-------- -KILTITSFS VIKG----RG EPYESSVFEA G-GYKWRLVL YVNGNQN--D
At2M6b/1-420     G--MWRDERP SN-------- -KILTITSFS VIKG----RG EPYESSVFEA G-GYKWRLVL YVNGNQN--D
At2M6c/1-298     ---MWRDERP SN-------- -KILTITSFS VIKG----RG EPYESSVFEA G-GYKWRLVL YVNGNQN--D
At2M6d/1-298     ---MWRDERP SN-------- -KILTITSFS VIKG----RG EPYESSVFEA G-GYKWRLVL YVNGNQN--D
At2M7/1-743      ---MWNQKP- -C-------- -FRFEIDNFS E-------KK DVIASKAFVS G-GCEWFLYL YPKGQSL--N
At2M8/1-318      ---QLKERKN AH-------- -FML-VDGMS KLLTE---KV NNFQSLDFQV S-GLKWRLLI QPAVGVK---
At2M9/1-416      ---KLKDRKN AH-------- -FML-VDGMS KLLTE---KV KNCQSLDFQV S-GVKWRLVI RLSRGRK---
At2M10/1-411     VTRTLRDEPP SH-------- -RILTITNFS EIIG----RE EPYESSVFEA YFEHKWRLIL YVNGNQN--D
At2M11/1-309     ---SISDAPP TH-------- -YMVKIESFS LLTKH---AI ERYETESFEA G-GYKWKLVL YPNGNKS--K
At2M12/1-370     ---IWRVIPP ST-------- -YCLKIESFI KFATSP--NA EKYESRPFES G-GYNWTLIV YPKGNIK--E
At2M13/1-363     ---GLRERPP SS-------- -YSLKMESFN TLMKSV--YT ERYESRPFRV G-RYNWTLVV YPKGNKN--D
At2M14/1-291     ---YWRERPP TT-------- -YSVTFESFG KMMELV--NN GYYESLPFTV D-GINWTFKI YPNGNS---D
At2M15/1-379     ---GLRDRPP SS-------- -YSLKMESFN TLLKST--YT EKYVSRPFSV G-GYNWTLVV FPNGNKK--D
At2M16/1-375     ---GLTEVPP SS-------- -YSFKIESYN SFLKI---PY LGFESRPFAA G-GYNWVLKV HPNGLTW--D
At2M17/1-358     ---MGNHQAD KK-------- -FAWVIKNFN SL------DT TRVYSDTFKA G-RCKWRLVA YPKRR----D
At2M18/1-427     ---TLREEAP SS-------- -YLMKLVGFS EVKF----SH QPYESADFDA A-GHKWRLIF YPAGKVE--E
At2M19a/1-297    ---TIREEAP SS-------- -YLMKLVGFS EVKF----SH QPYESADFDV G-GHKWRLIF YPAGKLE--E
At2M19b/1-297    ---TIREEAP SS-------- -YLMKLVGFS EVKF----SH QPYESADFDV G-GHKWRLIF YPAGKLE--E
At2M20/1-299     ---LFKIRHT TS-------- -HLFKIDNFS LLKKH---GI EKVESSVFDL A-GHKWKLSV YPNGHKN--A
At2M21/1-300     ---LFKVRHA TA-------- -HMFKIDHFS LLRKH---GI EKVESSVFDL A-GHKWKLSV HPNGHTN--A
At2M22/1-351     ---ELRDERP SS-------- -KIVTITSFS VIKD----RG EPYESSIFEA A-GYKWRLVL YVKGNPK--G
At2M23a/1-350    ---ELREERP SS-------- -KIVTITSFS VIKG----RG EPYESSVFEA A-GYKWRLVL YVNGNKN--D
At2M23b/1-327    ---ELREERP SS-------- -KIVTITSFS VIKG----RG EPYESSVFEA A-GYKW---- ----------
At2M24/1-333     ---VWRDDRP SD-------- -KILSITSFS IIRT----RP EPYESSVFEA V-GYKWRLVL YVNGNEK--D
At2M25/1-352     ---VLREERP SS-------- -KIVTITSFS VIKG----RS EAFESSTFEA A-GYKWRFML FVNGNQNDPD
Os2M1/1-685      ---FFRKRST DALLVKIHNP VFLWKVYGFS ALLQR---GA LAAKSAAFHC S-GYNWYLKV SPMHKTL--G

             211
At2M1/1-294      NGS-GFISMY VELDSSSLME SKPPTEVFAE LRFFVYNKKQ NKYFT-KD-- ---------- ----------
At2M2/1-396      DSG-GYVSIY VRVDNSSL-- ITNPKDVYAE ITFLAYKSST DKYQISQE-- ---------- ----------
At2M3a/1-296     NGS-GFVSMY VECLSSTT-- --PPIDVFAY LTFFVFSEEE KKYLSFQD-- ---------- ----------
At2M3b/1-296     NGS-GFVSMY VECLSSTT-- --PPIDVFAY LTFFVFSEEE KKYLSFQD-- ---------- ----------
At2M4/1-365      KGS-GFISMY VEFDNTKV-S STSPMEVFAY IIFFVYNKKE NKYFTIQD-- ---------- ----------
At2M5/1-898      ----DHLAVT LSVPNLES-- LRYGWERRTK YSFIVLNQSG RELERTFE-- ---------- ----------
At2M6a/1-420     GGN-NHISLY VRIEETES-- LPKGWEVNVE LKLFVYNGKQ RKYLIVKD-- ---------- ----------
At2M6b/1-420     GGN-NHISLY VRIEETES-- LPKGWEVNVE LKLFVYNGKQ RKYLIVKD-- ---------- ----------
At2M6c/1-298     GGN-NHISLY VRIEETES-- LPKGWEVNVE LKLFVYNGKQ RKYLIVKD-- ---------- ----------
At2M6d/1-298     GGN-NHISLY VRIEETES-- LPKGWEVNVE LKLFVYNGKQ RKYLIVKD-- ---------- ----------
At2M7/1-743      D---DHMSLY LSVANSKS-- LGSGWKRSAK FYFSVLNESD KELYRSTI-- ---------- ----------
At2M8/1-318      ----DYLSVA VWIIDEKC-- TGPNWEVKFN FKIGLLPQTG PEYFY----- ---------- ----------
At2M9/1-416      ----DHLSFV LEITDEKC-- TGSTWDVKFN FKIGIVPQTG PDYCF----- ---------- ----------
At2M10/1-411     GGS-NHISLY LRSEETDH-- LTYDGSINFV LKLFVYNGKQ DKYLTVTD-- ---------- ----------
At2M11/1-309     NTK-DHVSVY LSLADSSS-- LSPGWEVYAV FRLYLLDQNK DNYLILQ--- ---------- ----------
At2M12/1-370     GAPLNYVSMY VQIDNSTL-- LNSPKEVYAE VKFFIYNRKE DKYLTYQE-- ---------- ----------
At2M13/1-363     NGT-GHISLY VVLDNSTL-- TSQSEEVHVD LRFYVFNKKE TKYFTIQD-- ---------- ----------
At2M14/1-291     TTR-GLIYLY VKIDDSSI-- TDPPLDVYAE IKFFVYNYGI SEYYTYQE-- ---------- ----------
At2M15/1-379     SGS-GYLSLY VAIDNSTL-- --GQQEIYAD LRFYIFNKNE RKYFTIQD-- ---------- ----------
At2M16/1-375     GTS-GYVSLY VLLHESTP-- ITADQVVYAD LRFYIFNNNE KKYFTVQD-- ---------- ----------
At2M17/1-358     RYT-TSFSLF LCVPDSES-- LPSGWRRRA- -KLIGYN--- ---------- ---------- ----------
At2M18/1-427     GGK-DHISIY ARVENVGA-- --SEMQIDVE LKFFLYNHNA KKYSVFQD-- ---------- ----------
At2M19a/1-297    GGK-DHVSIY ARIENVGA-- ---SMQIDAE LKFFIYNHNN KQYSVFQD-- ---------- ----------
At2M19b/1-297    GGK-DHVSIY ARIENVGA-- ---SMQIDAE LKFFIYNHNN KQYSVFQD-- ---------- ----------
At2M20/1-299     KGT--HVSMF LVNQVPVNDM PTYELLVVSQ LERKWHTHGR DEFDINPE-- ---------- ----------
At2M21/1-300     KGT-HYVSLY LMNQAPVYDT LTYELLAVSQ LEPKWHTHGR DEYETNEE-- ---------- ----------
At2M22/1-351     GIN-NHISLY ARIEETET-- LPRGWEVNVD LKLFVHNRKL KKYLSVTD-- ---------- ----------
At2M23a/1-350    GGN-DHISLY ARIEETNS-- LPLGWEVNVD LKLFVHNGKL HKYLTVTD-- ---------- ----------
At2M23b/1-327    ---------- --IEETNS-- LPLGWEVNVD LKLFVHNGKL HKYLTVTD-- ---------- ----------
At2M24/1-333     GGK-DHVSLY AKIVETES-- LPVGWEVNVD LKLFVYNGKL NKYLIVT--- ---------- ----------
At2M25/1-352     GGH-ENMALY VGIKETES-- FPRGWEVNVD LKLFVHNEKL HKYLTVSD-- ---------- ----------
Os2M1/1-685      DGT-PHVALS LVLSRLSF-- -KPDYTMNAV FVLSMYNHSK GNFLVVKEVL FLQKKKFVSV QNLFLQKKDF
```

```
               281
At2M1/1-294    ---VEIKRFD AL-------- ---------- ---------- ---------- ---------- ----------
At2M2/1-396    ---TEAQRFH LF-------- ---------- ---------- ---------- ---------- ----------
At2M3a/1-296   ---VEVKRFN SS-------- ---------- ---------- ---------- ---------- ----------
At2M3b/1-296   ---VEVKRFN SS-------- ---------- ---------- ---------- ---------- ----------
At2M4/1-365    ---VEVKRFN AL-------- ---------- ---------- ---------- ---------- ----------
At2M5/1-898    ----VEGLFC TE-------- ---------- ---------- ---------- ---------- ----------
At2M6a/1-420   ---GIVKRYN DA-------- ---------- ---------- ---------- ---------- ----------
At2M6b/1-420   ---GIVKRYN DA-------- ---------- ---------- ---------- ---------- ----------
At2M6c/1-298   ---GIVKRYN DA-------- ---------- ---------- ---------- ---------- ----------
At2M6d/1-298   ---GIVKRYN DA-------- ---------- ---------- ---------- ---------- ----------
At2M7/1-743    --SQEFCLFC VQ-------- ---------- ---------- ---------- ---------- ----------
At2M8/1-318    ---VSVGCHN EK-------- ---------- ---------- ---------- ---------- ----------
At2M9/1-416    ---VLVGHQN EK-------- ---------- ---------- ---------- ---------- ----------
At2M10/1-411   ---GIQKRYN YK-------- ---------- ---------- ---------- ---------- ----------
At2M11/1-309   ---GNERRFH SV-------- ---------- ---------- ---------- ---------- ----------
At2M12/1-370   ---TDAKRFF LF-------- ---------- ---------- ---------- ---------- ----------
At2M13/1-363   ---TDVWRFS AI-------- ---------- ---------- ---------- ---------- ----------
At2M14/1-291   ---VEPVKFD SV-------- ---------- ---------- ---------- ---------- ----------
At2M15/1-379   ---TDVWKFS VF-------- ---------- ---------- ---------- ---------- ----------
At2M16/1-375   ---TNVWKFT AP-------- ---------- ---------- ---------- ---------- ----------
At2M17/1-358   ------KKYS SS-------- ---------- ---------- ---------- ---------- ----------
At2M18/1-427   ---GTVKHYS KE-------- ---------- ---------- ---------- ---------- ----------
At2M19a/1-297  ---GTMKHYN KE-------- ---------- ---------- ---------- ---------- ----------
At2M19b/1-297  ---GTMKHYN KE-------- ---------- ---------- ---------- ---------- ----------
At2M20/1-299   ---------- ---------- ---------- ---------- ---------- ---------- ----------
At2M21/1-300   ---------- ---------- ---------- ---------- ---------- ---------- ----------
At2M22/1-351   ---GTVKRYN DA-------- ---------- ---------- ---------- ---------- ----------
At2M23a/1-350  ---GLVKRYN NA-------- ---------- ---------- ---------- ---------- ----------
At2M23b/1-327  ---GLVKRYN NA-------- ---------- ---------- ---------- ---------- ----------
At2M24/1-333   -----VKRYN NA-------- ---------- ---------- ---------- ---------- ----------
At2M25/1-352   ---GTVKRYE AA-------- ---------- ---------- ---------- ---------- ----------
Os2M1/1-685    TKGDYTWTMN NFPELDLKPS VLSPAFEIGR RKWFIRMYPR GDEYSTNSLS MYLFPQSWDK LLPEPGMMIE

               351
At2M1/1-294    ---------- ---------- ----KMVWGL PK-------- ---------- ---------- ----------
At2M2/1-396    ---------- ---------- ----KQQWGL LQFLPIYYFE NP-------- ---------- ----------
At2M3a/1-296   ---------- ---------- ----KTVWGL SKALPVETLK DR-------- ---------- ----------
At2M3b/1-296   ---------- ---------- ----KTVWGL SKALPVETLK DR-------- ---------- ----------
At2M4/1-365    ---------- ---------- ----RTVWGL SQVLSLETFN DL-------- ---------- ----------
At2M5/1-898    ---------- ---------- ----LLEWCH PKVMPTNKLQ EVCLENNKLI IEVQVKVLEV VHEGGVTTEK
At2M6a/1-420   ---------- ---------- ----KKEWGY GKLIPLTTFL DT-------- ---------- ----------
At2M6b/1-420   ---------- ---------- ----KKEWGY GKLIPLTTFL DT-------- ---------- ----------
At2M6c/1-298   ---------- ---------- ----KKEWGY GKLIPLTTFL DT-------- ---------- ----------
At2M6d/1-298   ---------- ---------- ----KKEWGY GKLIPLTTFL DT-------- ---------- ----------
At2M7/1-743    ---------- ---------- ----ALAWGI RKALPLSKFE EK-------- ---------- ----------
At2M8/1-318    ---------- ---------- ----QPAQGV VKFITHTQLK ER-------- ---------- ----------
At2M9/1-416    ---------- ---------- ----KRSQGL ANFISHTDLK ER-------- ---------- ----------
At2M10/1-411   ---------- ---------- ----NKEWGY GKLIPLSTFL DT-------- ---------- ----------
At2M11/1-309   ---------- ---------- ----KREWGF DKFIPTGTFS DA-------- ---------- ----------
At2M12/1-370   ---------- ---------- ----KPYWGY GNVRPYTDVA NP-------- ---------- ----------
At2M13/1-363   ---------- ---------- ----KRMWGF SKVLPLITFN NL-------- ---------- ----------
At2M14/1-291   ---------- ---------- ----QQEWGR ---------- ---------- ---------- ----------
At2M15/1-379   ---------- ---------- ----KTMWGF SQVLPIDTFK DP-------- ---------- ----------
At2M16/1-375   ---------- ---------- ----KRLLGF PKVMSADQFE DL-------- ---------- ----------
At2M17/1-358   ---------- ---------- ----RTM--- ---------- ---------- ---------- ----------
At2M18/1-427   ---------- ---------- ----KKECGF AQMLLRSKFN DP-------- ---------- ----------
At2M19a/1-297  ---------- ---------- ----KKECGF AQMLLFSKFN DP-------- ---------- ----------
At2M19b/1-297  ---------- ---------- ----KKECGF AQMLLFSKFN DP-------- ---------- ----------
At2M20/1-299   ---------- ---------- ----PASEGF LRFISLADLE ---------- ---------- ----------
At2M21/1-300   ---------- ---------- ----LGSEGF REFISLVDLK ---------- ---------- ----------
At2M22/1-351   ---------- ---------- ----KKEWGF TQLISLPTFY NA-------- ---------- ----------
At2M23a/1-350  ---------- ---------- ----KKEWGF GQLIPRSTFY NA-------- ---------- ----------
At2M23b/1-327  ---------- ---------- ----KKEWGF GQLIPRSTFY NA-------- ---------- ----------
At2M24/1-333   ---------- ---------- ----TKELGY GQLIPQSTFY DG-------- ---------- ----------
At2M25/1-352   ---------- ---------- ----KTYWGF GNLIPRTTLL DP-------- ---------- ----------
Os2M1/1-685    LTLSILNQNN AQLHKVSGRF VFASKNGWGW SNFIALNKLK DLVGSSCIVK ADITIIGSSS ESQIVYMLRL
```

```
              421
At2M1/1-294   ---------- ---------- ---------- ---------- ---------- ---------- ----------
At2M2/1-396   ---------- ---------- ---------- ---------- ---------- ---------- ----------
At2M3a/1-296  ---------- ---------- ---------- ---------- ---------- ---------- ----------
At2M3b/1-296  ---------- ---------- ---------- ---------- ---------- ---------- ----------
At2M4/1-365   ---------- ---------- ---------- ---------- ---------- ---------- ----------
At2M5/1-898   EMFNIEGFDV LYTQVSRVSW LFVEHPNIAV DVRIKNQLVR TAYINVLLGL IETLDRSPRS LSETDLRDAH
At2M6a/1-420  ---------- ---------- ---------- ---------- ---------- ---------- ----------
At2M6b/1-420  ---------- ---------- ---------- ---------- ---------- ---------- ----------
At2M6c/1-298  ---------- ---------- ---------- ---------- ---------- ---------- ----------
At2M6d/1-298  ---------- ---------- ---------- ---------- ---------- ---------- ----------
At2M7/1-743   ---GFLEKDK LIVEVYIKNF EAVDGEGGGV SKKEEEETVE IIGSQDYASQ ASFKGSVTLV RKIFAEHPEI
At2M8/1-318   ---------- ---------- ---------- ---------- ---------- ---------- ----------
At2M9/1-416   ---------- ---------- ---------- ---------- ---------- ---------- ----------
At2M10/1-411  ---------- ---------- ---------- ---------- ---------- ---------- ----------
At2M11/1-309  ---------- ---------- ---------- ---------- ---------- ---------- ----------
At2M12/1-370  ---------- ---------- ---------- ---------- ---------- ---------- ----------
At2M13/1-363  ---------- ---------- ---------- ---------- ---------- ---------- ----------
At2M14/1-291  ---------- ---------- ---------- ---------- ---------- ---------- ----------
At2M15/1-379  ---------- ---------- ---------- ---------- ---------- ---------- ----------
At2M16/1-375  ---------- ---------- ---------- ---------- ---------- ---------- ----------
At2M17/1-358  ---------- ---------- ---------- ---------- ---------- ---------- ----------
At2M18/1-427  ---------- ---------- ---------- ---------- ---------- ---------- ----------
At2M19a/1-297 ---------- ---------- ---------- ---------- ---------- ---------- ----------
At2M19b/1-297 ---------- ---------- ---------- ---------- ---------- ---------- ----------
At2M20/1-299  ---------- ---------- ---------- ---------- ---------- ---------- ----------
At2M21/1-300  ---------- ---------- ---------- ---------- ---------- ---------- ----------
At2M22/1-351  ---------- ---------- ---------- ---------- ---------- ---------- ----------
At2M23a/1-350 ---------- ---------- ---------- ---------- ---------- ---------- ----------
At2M23b/1-327 ---------- ---------- ---------- ---------- ---------- ---------- ----------
At2M24/1-333  ---------- ---------- ---------- ---------- ---------- ---------- ----------
At2M25/1-352  ---------- ---------- ---------- ---------- ---------- ---------- ----------
Os2M1/1-685   IYWRRDLKRE LEERTMGNVA GRAAGALLFC ITADDPDPRF MASLRYFSEE PHQSPLICST TVGTPGLKED

              491
At2M1/1-294   ---------- ---------- ---------- ---------- ---------- ---------- ----------
At2M2/1-396   ---------- ---------- ---------- ---------- ---------- ---------- ----------
At2M3a/1-296  ---------- ---------- ---------- ---------- ---------- ---------- ----------
At2M3b/1-296  ---------- ---------- ---------- ---------- ---------- ---------- ----------
At2M4/1-365   ---------- ---------- ---------- ---------- ---------- ---------- ----------
At2M5/1-898   IELSELTEAG FKVDWLKKKL EEVSLARKND ISDGSQVEEL EEHVKNLKLE LDNEKIKSST ASERVLLLEK
At2M6a/1-420  ---------- ---------- ---------- ---------- ---------- ---------- ----------
At2M6b/1-420  ---------- ---------- ---------- ---------- ---------- ---------- ----------
At2M6c/1-298  ---------- ---------- ---------- ---------- ---------- ---------- ----------
At2M6d/1-298  ---------- ---------- ---------- ---------- ---------- ---------- ----------
At2M7/1-743   AEEFKPKNQV FKKEYMNILR NAYRKVSELA EVKMDWVKSK IEEVSLEIKK RNDEVSEVPL DNKIADDDDD
At2M8/1-318   ---------- ---------- ---------- ---------- ---------- ---------- ----------
At2M9/1-416   ---------- ---------- ---------- ---------- ---------- ---------- ----------
At2M10/1-411  ---------- ---------- ---------- ---------- ---------- ---------- ----------
At2M11/1-309  ---------- ---------- ---------- ---------- ---------- ---------- ----------
At2M12/1-370  ---------- ---------- ---------- ---------- ---------- ---------- ----------
At2M13/1-363  ---------- ---------- ---------- ---------- ---------- ---------- ----------
At2M14/1-291  ---------- ---------- ---------- ---------- ---------- ---------- ----------
At2M15/1-379  ---------- ---------- ---------- ---------- ---------- ---------- ----------
At2M16/1-375  ---------- ---------- ---------- ---------- ---------- ---------- ----------
At2M17/1-358  ---------- ---------- ---------- ---------- ---------- ---------- ----------
At2M18/1-427  ---------- ---------- ---------- ---------- ---------- ---------- ----------
At2M19a/1-297 ---------- ---------- ---------- ---------- ---------- ---------- ----------
At2M19b/1-297 ---------- ---------- ---------- ---------- ---------- ---------- ----------
At2M20/1-299  ---------- ---------- ---------- ---------- ---------- ---------- ----------
At2M21/1-300  ---------- ---------- ---------- ---------- ---------- ---------- ----------
At2M22/1-351  ---------- ---------- ---------- ---------- ---------- ---------- ----------
At2M23a/1-350 ---------- ---------- ---------- ---------- ---------- ---------- ----------
At2M23b/1-327 ---------- ---------- ---------- ---------- ---------- ---------- ----------
At2M24/1-333  ---------- ---------- ---------- ---------- ---------- ---------- ----------
At2M25/1-352  ---------- ---------- ---------- ---------- ---------- ---------- ----------
Os2M1/1-685   IVVDTT---- ---------- ---------- ---------- ---------- ---------- -------FEL
```

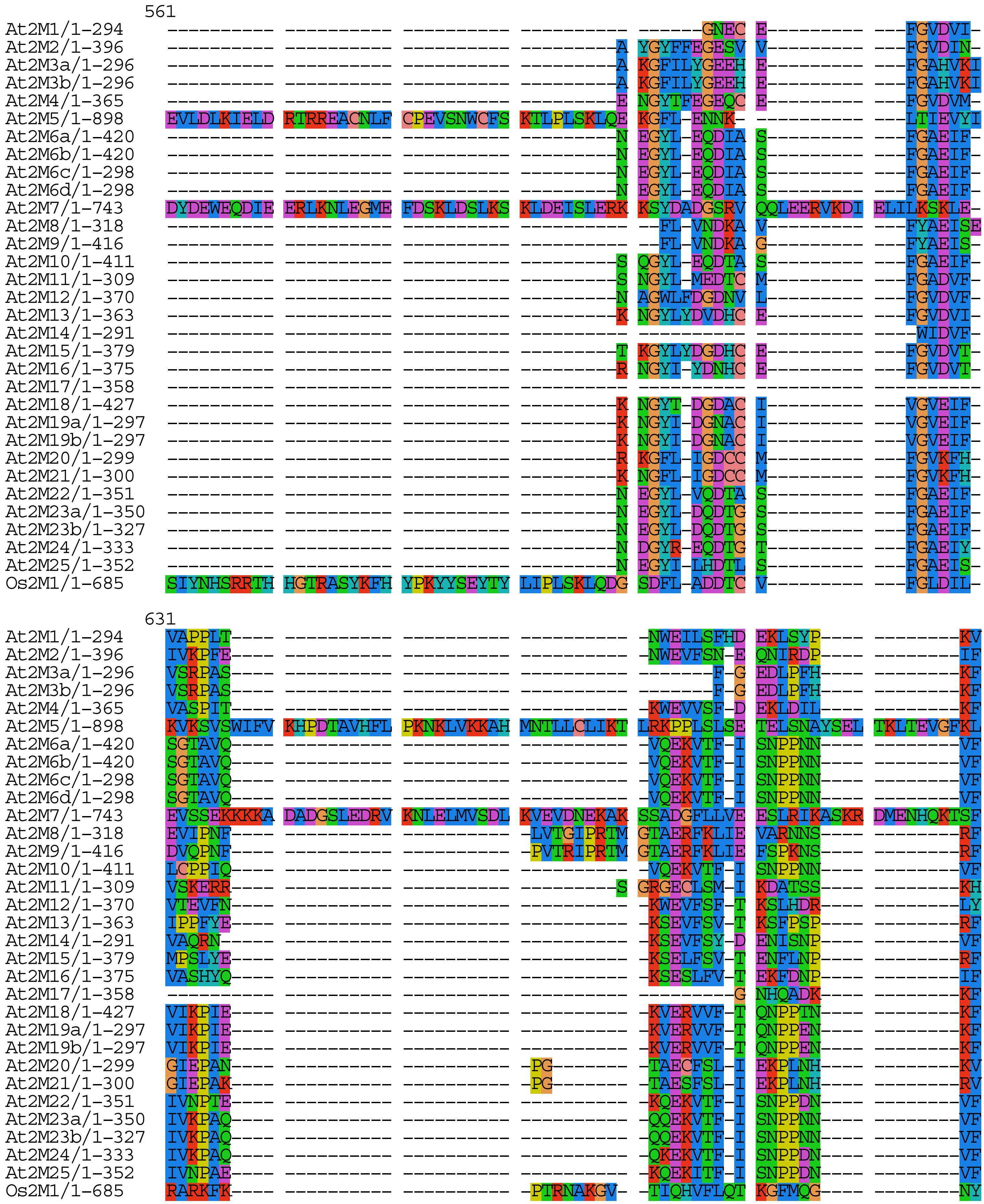

561
At2M1/1-294
At2M2/1-396
At2M3a/1-296
At2M3b/1-296
At2M4/1-365
At2M5/1-898
At2M6a/1-420
At2M6b/1-420
At2M6c/1-298
At2M6d/1-298
At2M7/1-743
At2M8/1-318
At2M9/1-416
At2M10/1-411
At2M11/1-309
At2M12/1-370
At2M13/1-363
At2M14/1-291
At2M15/1-379
At2M16/1-375
At2M17/1-358
At2M18/1-427
At2M19a/1-297
At2M19b/1-297
At2M20/1-299
At2M21/1-300
At2M22/1-351
At2M23a/1-350
At2M23b/1-327
At2M24/1-333
At2M25/1-352
Os2M1/1-685
631
At2M1/1-294
At2M2/1-396
At2M3a/1-296
At2M3b/1-296
At2M4/1-365
At2M5/1-898
At2M6a/1-420
At2M6b/1-420
At2M6c/1-298
At2M6d/1-298
At2M7/1-743
At2M8/1-318
At2M9/1-416
At2M10/1-411
At2M11/1-309
At2M12/1-370
At2M13/1-363
At2M14/1-291
At2M15/1-379
At2M16/1-375
At2M17/1-358
At2M18/1-427
At2M19a/1-297
At2M19b/1-297
At2M20/1-299
At2M21/1-300
At2M22/1-351
At2M23a/1-350
At2M23b/1-327
At2M24/1-333
At2M25/1-352
Os2M1/1-685

```
                 701
At2M1/1-294      TW-------- ---------- ---------- ---------- ---------- ---------- ------SVKN
At2M2/1-396      EW-------- ---------- ---------- ---------- ---------- ---------- ------RLTK
At2M3a/1-296     SW-------- ---------- ---------- ---------- ---------- ---------- ------TIRD
At2M3b/1-296     SW-------- ---------- ---------- ---------- ---------- ---------- ------TIRD
At2M4/1-365      SW-------- ---------- ---------- ---------- ---------- ---------- ------SVKD
At2M5/1-898      DWLKSKLEKA SLERKKSVSD GSQISAYDSR IKQISKYFVC FLLPRRTTKT LKSKQMGTQF RKALTLTVTN
At2M6a/1-420     TW-------- ---------- ---------- ---------- ---------- ---------- ------KILH
At2M6b/1-420     TW-------- ---------- ---------- ---------- ---------- ---------- ------KILH
At2M6c/1-298     TW-------- ---------- ---------- ---------- ---------- ---------- ------KILH
At2M6d/1-298     TW-------- ---------- ---------- ---------- ---------- ---------- ------KILH
At2M7/1-743      TF-------- ---------- ---------- ---------- ---------- ---------- ------EIEN
At2M8/1-318      TW-------- ---------- ---------- ---------- ---------- ---------- ------KITK
At2M9/1-416      TW-------- ---------- ---------- ---------- ---------- ---------- ------KITQ
At2M10/1-411     TW-------- ---------- ---------- ---------- ---------- ---------- ------KILH
At2M11/1-309     VW-------- ---------- ---------- ---------- ---------- ---------- ------KIEN
At2M12/1-370     KW-------- ---------- ---------- ---------- ---------- ---------- ------TLPN
At2M13/1-363     TW-------- ---------- ---------- ---------- ---------- ---------- ------YIQG
At2M14/1-291     TW-------- ---------- ---------- ---------- ---------- ---------- ------SLPN
At2M15/1-379     TW-------- ---------- ---------- ---------- ---------- ---------- ------TIRG
At2M16/1-375     TY-------- ---------- ---------- ---------- ---------- ---------- ------ALLR
At2M17/1-358     TW-------- ---------- ---------- ---------- ---------- ---------- ------VIKN
At2M18/1-427     TW-------- ---------- ---------- ---------- ---------- ---------- ------KISD
At2M19a/1-297    TW-------- ---------- ---------- ---------- ---------- ---------- ------KISH
At2M19b/1-297    TW-------- ---------- ---------- ---------- ---------- ---------- ------KISH
At2M20/1-299     TW-------- ---------- ---------- ---------- ---------- ---------- ------MMSK
At2M21/1-300     TW-------- ---------- ---------- ---------- ---------- ---------- ------MMTM
At2M22/1-351     TW-------- ---------- ---------- ---------- ---------- ---------- ------KILR
At2M23a/1-350    TW-------- ---------- ---------- ---------- ---------- ---------- ------KILR
At2M23b/1-327    TW-------- ---------- ---------- ---------- ---------- ---------- ------KILR
At2M24/1-333     TW-------- ---------- ---------- ---------- ---------- ---------- ------KILH
At2M25/1-352     TW-------- ---------- ---------- ---------- ---------- ---------- ------KILR
Os2M1/1-685      TW-------- ---------- ---------- ---------- ---------- ---------- ------NIED

                 771
At2M1/1-294      FSQWKENECS KPNRFSIGGR EWVLKLFPKG NSRAKGKYLS VFLYLADNET -L--KPDEKI FTQVVVRILN
At2M2/1-396      FSTRFLD-SY TSDSFSSGGR NWALKVYPNG VGNATGNSLS LYLLSD---- ----QSNDKG YVEAKLRVID
At2M3a/1-296     FALLEQN-DY VSKTFHMGEK DWTLKLYPKG DSEADDKLIQ -HLHLADGET -L--AKGELI FVRVNLKVLD
At2M3b/1-296     FALLEQN-DY VSKTFHMGEK DWTLKLYPKG DSEADDKLIQ -HLHLADGET -L--AKGELI FVRVNLKVLD
At2M4/1-365      FSVLKEE-FY VSERFSMGGR LWDLQMYPKG DPRRDKKWLS IFLRLSGSET -L--TVDEKI YVIAHLRVLD
At2M5/1-898      FSQKSSP--I NSPPFPSGGC NWYIKFYPKG --SADDNYLS LFLSPDDPKS -L--GLNWKR RANFYFVLLN
At2M6a/1-420     FSNLEDK-FY YSDDFLVEDR YWRLGFNPKG TGDGRSQAIP IFLYAQGHKP -N--AVATNT WGAVNLRLKN
At2M6b/1-420     FSNLEDK-FY YSDDFLVEDR YWRLGFNPKG TGDGRSQAIP IFLYAQGHKP -N--AVATNT WGAVNLRLKN
At2M6c/1-298     FSNLEDK-FY YSDDFLVEDR YWRLGFNPKG TGDGRSQAIP IFLYAQGHKP -N--AVATNT WGAVNLRLKN
At2M6d/1-298     FSNLEDK-FY YSDDFLVEDR YWRLGFNPKG TGDGRSQAIP IFLYAQGHKP -N--AVATNT WGAVNLRLKN
At2M7/1-743      FS--ERKYLI WSPIFISGQC HWFVKVYPIK --DNNYDHVS VYLHVANPQS -L--RPGWKR RAHFSLILSN
At2M8/1-318      FSSFNGE-EH SSYEFTVGPR RWKLVMYPRG TGDGKGNSLS LYLNASNYVT NN--GPKGRT FAVYKLRVLD
At2M9/1-416      FSSFDGE-EH SSYEFTVGPR RWKLVMYPKG NGDGKGNSLS LYLFASDYVT -N--GPKGGT LAIYKLRVLD
At2M10/1-411     FSTLEDI-VY YSDDFLVEDR YWRLGVNPKG TGDGRSQAIK IFLYAQGHKP -N--AVVSST WGAVNLRVKN
At2M11/1-309     FSKLDKE-SY DSNAFFAGDR KWKIEFYPTG TKQGTGTHLS IYLTLVDPET -I--SDGTKI FVEFTIRIFD
At2M12/1-370     FSSLEKQ-YY VSDKFVIGGR SWALKVYPSG DGEGQGNSLS LYVVAVDVKP -------YDKI YLKAKLRIIN
At2M13/1-363     YSTLPTD--Y LSEEFIIGGK SWNLRIFKNG FGAFEGKNLS LYLNLGPQEL -LKAKPYDKV YVRAKLRVPN
At2M14/1-291     FSTLTLD-SY TSDPFSSGDR NWVLKVYPNG DGVGKDNSLS LYLLSESNEK -N-------- YVRATLRVLN
At2M15/1-379     FSTLLKN-SY LSEVFSIGGR SWNIQINPSG LGTGEGKALS MYLGLNVNEI -F--RPYEKI YVRAKLRALN
At2M16/1-375     FSTLLKE-SY QSDVFSIGGR SMYLQVFPNG RNLSKGKAMS LYLNINDK-- -F--KPFEMI YVRAKLRVLN
At2M17/1-358     YNSLGSG-SV YSDTFKAGRC KWRLLAFPKG --NNIYDYFF LYICVPNSES -L--PSGWRR RAKVSFTMVN
At2M18/1-427     FSKLGDKKYH YSDEFVVGER KWRIKISPKG --DKKVRALS VYVQAMEYLP -N--AVASTT YAKLKLQLMN
At2M19a/1-297    FSYIGDKRYY YSDEFVVGDR KWRLKISPKG --DKKVRALS VYVQAMAYLP -N--AVASST YAKLRLRLLN
At2M19b/1-297    FSYIGDKRYY YSDEFVVGDR KWRLKISPKG --DKKVRALS VYVQAMAYLP -N--AVASST YAKLRLRLLN
At2M20/1-299     FSSFNPGKAH QSNEFVVGTR KWRLEVHPRG YMDEKDKSFS VYLSAEGFVN -N--APMTKT YAKFKLRVLD
At2M21/1-300     FSSFNPGNVH QSNEFVVGTR KWRIKVHPRG SMGEKDKSFS VYLSALGFVN -N--APKTKT YARFKLRVLD
At2M22/1-351     FSTLEDK-FY YSDDFLVGDR YWRLGFNPKG SGGGRPHALP IFLYAQGHKA -N--AVVTNT WGAVNLRLKN
At2M23a/1-350    FSTLEDK-FY YSDDFLVEDR YWRLGFNPKG DGGGRPHALP IFLFAQGHKA -N--AVATNT WGAVNLRLKN
At2M23b/1-327    FSTLEDK-FY YSDDFLVEDR YWRLGFNPKG DGGGRPHALP IFLFAQGHKA -N--AVATNT WGAVNLRLKN
At2M24/1-333     FSTLEDK-VY QSNEFLVGDR YWKLGLNPKG ------GLVP IFLYAQGFKA -N--AVVTTT YAATNLRLKN
At2M25/1-352     FSTLENK-FY YSDEFLVGDR YWRLGFNPKG YQGERPRALS IFLYAQGYKA -N--AVITNT WGSVNLQLKN
Os2M1/1-685      -SKLDLKSII CSPKFDIGEH KWYLRVDPYG DYRNRD-YVS IYLCLDDNSN MP--PIESAI MAEFIISILN
```

```
              841
At2M1/1-294    PLGSNH---V ASR--LNY-- ---------W HKGS--NFG- --YGW----C KFLSLDKIRK T---YLDKED
At2M2/1-396    QIQSNN---F EKK--VAA-- ---------W PNAT--ENG- --WGF----D RFLSFADIKN TSKGFLVND-
At2M3a/1-296   PRGSNH---L TGS--LNC-- ---------W LMNS--NKA- --WGL----P QSMSFDKNEG A---YLDREG
At2M3b/1-296   PRGSNH---L TGS--LNC-- ---------W LMNS--NKA- --WGL----P QSMSFDKNEG A---YLDREG
At2M4/1-365    PLGN------ ---------- ---------W FRDR--NKG- --WGY----L EFLSFSKLRK S---YLDLE-
At2M5/1-898    QSGKEL---H RTPEIGDQ-- ---------W FCDD--SLS- --WGF----P QTLPRKKLLD KI--FLDND-
At2M6a/1-420   QRSSNH---A QIY--SAA-- ---------W YPTR--SD-- --YGV--GVN TIISLAEFND ASKGYSVND-
At2M6b/1-420   QRSSNH---A QIY--SAA-- ---------W YPTR--SD-- --YGV--GVN TIISLAEFND ASKGYSVND-
At2M6c/1-298   QRSSNH---A QIY--SAA-- ---------W YPTR--SD-- --YGV--GVN TIISLAEFND ASKGYSVND-
At2M6d/1-298   QRSSNH---A QIY--SAA-- ---------W YPTR--SD-- --YGV--GVN TIISLAEFND ASKGYSVND-
At2M7/1-743    QSGKE----V KIP--SDSCD ---------L FCTE--LS-- --SSY----P KILPPIKLKE E--GFLEND-
At2M8/1-318    QLHRNH---F EID--CQD-- ---------W FLYDPVHPRL CSWGR----T KFLPLEELHK ASRGFLVND-
At2M9/1-416    QLNRNH---C ETE--CRY-- ---------W FPYNPVNQMD SLWGR----P KFLPLEELHK SSRGFLVND-
At2M10/1-411   QRSSNH---S QIY--SAA-- ---------L YPIR--ND-- --YGV--GVN TVLSLAELND AVKEYLVND-
At2M11/1-309   QLQGRH---I AGK--VTK-- ---------W FSRS--SSE- --HGW----V KYVSMVYFTQ PNSGLLLKD-
At2M12/1-370   QRDSKH---M EKK--VES-- ---------W SDQA--NS-- --WGF----Q KFVPFADLKD TSKGLLVND-
At2M13/1-363   QFGSQSNLVL ERP--LDN-- ---------W FSPQ--TIG- --WGY----A DFMPLSDLRN SSKGFLVND-
At2M14/1-291   QIGSDN---V EKP--VEG-- ---------W PNAA--ENG- --WGY----Q EFIPLADLQD ATKGFVVDD-
At2M15/1-379   QLNLSN---I ERE--LDI-- ---------W YNGP--GYGE YSWGF----P EFIYFPYLTD SSKGFVKND-
At2M16/1-375   QRKLNN---V EIQ--VSN-- ---------W YTS------- --WFYYSGDF QIIPLADLRD SSKGFVVND-
At2M17/1-358   QIPGGL---S QQRE-AVY-- ---------W FDEK--DTT- --HGF----E SMFLLSEIQS SDKGFLVNG-
At2M18/1-427   QKNTNH---I EKR--GTYQT SFFLYLVFHF FSRE--KE-- --DGY--GTS ELISVEDLND ESKGYLVED-
At2M19a/1-297  QKNSNH---I EKR--VFH-- ---------F YSRE--NG-- --DGS--GIS ELISVEDLND ESKGYLVED-
At2M19b/1-297  QKNSNH---I EKR--VFH-- ---------F YSRE--NG-- --DGS--GIS ELISVEDLND ESKGYLVED-
At2M20/1-299   QVSWNH---V EES--GLS-- ---------W FDAE--PSDQ --SGF----A DFMPLGKLNE P---YLVKD-
At2M21/1-300   QVSRNH---V EKT--ISG-- ---------W LGAE--PDDR --HGF----A DFMPLGELDD P---YLVKD-
At2M22/1-351   QRSSNH---K QLY--SAA-- ---------W YPIR--SD-- --YGV--GVN NIILMSELKD ASKGYMVND-
At2M23a/1-350  QRSTNH---R QIY--SAA-- ---------W YPIG--SG-- --YGV--GVN NIILLADLND ASKGYLVND-
At2M23b/1-327  QRSTNH---R QIY--SAA-- ---------W YPIG--SG-- --YGV--GVN NIILLADLND ASKGYLVND-
At2M24/1-333   QRSSNH---V TTY--TAY-- ---------W YLIP--SG-- --LGL--GVN T-IPLSDVKD ASKGYVVND-
At2M25/1-352   QRSSNH---I QLY--SEA-- ---------W CAIR--SG-- --YGI--EGN SIILLEDLQN SSKGYLVND-
Os2M1/1-685    QKNGKH---S QQK--ART-- ---------V FSCK--GIA- --WGW----H KFIRRDQMKN TNAGFVVGS-

              911
At2M1/1-294    TLMIEAEFEV VSATK----- ---------- ---------- ---------- ---------- ----------
At2M2/1-396    TLKLEVQILS FSKTD----- ---------- ---------- ---------- ---------- ----------
At2M3a/1-296   TLEVEIECEI KNSHK----- ---------- ---------- ---------- ---------- ----------
At2M3b/1-296   TLEVEIECEI KNSHK----- ---------- ---------- ---------- ---------- ----------
At2M4/1-365    DTFFPLNL-- ---------- ---------- ---------- ---------- ---------- ----------
At2M5/1-898    RFNIEIYIKV IEVVEGYHMF PASFTNKLLR SSLEYPDKSE KETVDINGFK VLSSQVTSVK RIFEEHPDIA
At2M6a/1-420   SIIFEAEMVK VSVTN----- ---------- ---------- ---------- ---------- ----------
At2M6b/1-420   SIIFEAEMVK VSVTN----- ---------- ---------- ---------- ---------- ----------
At2M6c/1-298   SIIFEAEMVK VSVTN----- ---------- ---------- ---------- ---------- ----------
At2M6d/1-298   SIIFEAEMVK VSVTN----- ---------- ---------- ---------- ---------- ----------
At2M7/1-743    KLIITVEVKV VEVVHPGELT GKEMVEFKEL QDLYNGVQQN KEVVKNCELM NMDMKQDSLK SN--------
At2M8/1-318    QIYIGVEFLI VSTTE----- ---------- ---------- ---------- ---------- ----------
At2M9/1-416    QIYIGVEISI VSTTE----- ---------- ---------- ---------- ---------- ----------
At2M10/1-411   SIIFEAEMVK VSVTN----- ---------- ---------- ---------- ---------- ----------
At2M11/1-309   VCLVEADVCV HGITS----- ---------- ---------- ---------- ---------- ----------
At2M12/1-370   TLKMEIEFED FSNTK----- ---------- ---------- ---------- ---------- ----------
At2M13/1-363   MLVVQVAMEE ISSTN----- ---------- ---------- ---------- ---------- ----------
At2M14/1-291   LLEVEVEIMA ISKQT----- ---------- ---------- ---------- ---------- ----------
At2M15/1-379   VLMVQVEMEA ISSTK----- ---------- ---------- ---------- ---------- ----------
At2M16/1-375   MLKVEVQLEG ISSTK----- ---------- ---------- ---------- ---------- ----------
At2M17/1-358   EVKIVAEVDV LEVIG----- ---------- ---ELDVPEE PERIDINGFQ VPASQVESMN SLFEKYRGFA
At2M18/1-427   TIVLETTLLC VTETK----- ---------- --VKLCDMCK YKFFLLNPRQ HAKSHWKKKK NMHNHLLQLW
At2M19a/1-297  SIVLETTLLW VSDTK----- ---------- ---------- ---------- ---------- ----------
At2M19b/1-297  SIVLETTLLW VSDTK----- ---------- ---------- ---------- ---------- ----------
At2M20/1-299   KLYVGVEFEV VSTTY----- ---------- ---------- ---------- ---------- ----------
At2M21/1-300   KLYVGVDFDV ISVSN----- ---------- ---------- ---------- ---------- ----------
At2M22/1-351   AIIFEAEMVK VSVTN----- ---------- ---------- ---------- ---------- ----------
At2M23a/1-350  AIIFEAEMVK VSITN----- ---------- ---------- ---------- ---------- ----------
At2M23b/1-327  AIIFEAEMVK VSITN----- ---------- ---------- ---------- ---------- ----------
At2M24/1-333   SIIIEVEMLT VSVTN----- ---------- ---------- ---------- ---------- ----------
At2M25/1-352   AIIFEAELVK VSVTN----- ---------- ---------- ---------- ---------- ----------
Os2M1/1-685    SWTVQAEVTV IGSSS----- ---------- ---------- ---------- ---------- ----------
```

```
               981
At2M1/1-294    ---------- ---FSTII-- ---------- ---------- ---------- ---------- ----------
At2M2/1-396    ---------- ---YYSHQSS LNVLTGDST- ---------- ---------- ---------- ----------
At2M3a/1-296   ---------- ---NHPFF-- ---------- ---------- ---------- ---------- ----------
At2M3b/1-296   ---------- ---NHPFF-- ---------- ---------- ---------- ---------- ----------
At2M4/1-365    ---------- ---------- ---------- ---------- ---------- ---------- ----------
At2M5/1-898    EDFRSKNQVV KTEYMSVLLR VIETMAKPPQ SISETELSNV HSELTELTEV GFKLEWLKAK LEEVCVAFKK
At2M6a/1-420   ---------- ---IVPI--- ---------- ---------- ---------- ---------- ----------
At2M6b/1-420   ---------- ---IVPI--- ---------- ---------- ---------- ---------- ----------
At2M6c/1-298   ---------- ---IVPI--- ---------- ---------- ---------- ---------- ----------
At2M6d/1-298   ---------- ---IVPI--- ---------- ---------- ---------- ---------- ----------
At2M7/1-743    ---------- ---HHEVSLK DKKRDDADES RFQKLEERLK NLELMELDCL KSKLEEVSIK NKKADADRSR
At2M8/1-318    ---------- ---YL----- ---------- ---------- ---------- ---------- ----------
At2M9/1-416    ---------- ---YL----- ---------- ---------- ---------- ---------- ----------
At2M10/1-411   ---------- ---IVPI--- ---------- ---------- ---------- ---------- ----------
At2M11/1-309   ---------- ---AI----- ---------- ---------- ---------- ---------- ----------
At2M12/1-370   ---------- ---YFPS--- ---------- ---------- ---------- ---------- ----------
At2M13/1-363   ---------- ---YLPK--- ---------- ---------- ---------- ---------- ----------
At2M14/1-291   ---------- -----PIN-- ---------- ---------- ---------- ---------- ----------
At2M15/1-379   ---------- ---YFPS--- ---------- ---------- ---------- ---------- ----------
At2M16/1-375   ---------- ---WYPS--- ---------- ---------- ---------- ---------- ----------
At2M17/1-358   SKIFPKNQHL RKTFLDVVLS MTEILCKFPE ELSSGDLAEA YSALRFVTKA GFKLDWLEKK LKETGKSRLQ
At2M18/1-427   CNPHNRLYVF GVVFLGLFDN VDASAEGEVY QI-------- ---------- ---------- ----------
At2M19a/1-297  ---------- ---VVDSI-- ---------- ---------- ---------- ---------- ----------
At2M19b/1-297  ---------- ---VVDSI-- ---------- ---------- ---------- ---------- ----------
At2M20/1-299   ---------- ---YC----- ---------- ---------- ---------- ---------- ----------
At2M21/1-300   ---------- ---YC----- ---------- ---------- ---------- ---------- ----------
At2M22/1-351   ---------- ---IVSV--- ---------- ---------- ---------- ---------- ----------
At2M23a/1-350  ---------- ---IVSA--- ---------- ---------- ---------- ---------- ----------
At2M23b/1-327  ---------- ---IVSA--- ---------- ---------- ---------- ---------- ----------
At2M24/1-333   ---------- ---IVSA--- ---------- ---------- ---------- ---------- ----------
At2M25/1-352   ---------- ---IVSA--- ---------- ---------- ---------- ---------- ----------
Os2M1/1-685    ---------- ---HA----- ---------- ---------- ---------- ---------- ----------

              1051
At2M1/1-294    ---------- ---------- ---------- ---------- ---------- ---------- ----------
At2M2/1-396    ---------- ---------- ---------- ---------- ---------- ---------- ----------
At2M3a/1-296   ---------- ---------- ---------- ---------- ---------- ---------- ----------
At2M3b/1-296   ---------- ---------- ---------- ---------- ---------- ---------- ----------
At2M4/1-365    ---------- ---------- ---------- ---------- ---------- ---------- ----------
At2M5/1-898    ANADGCRIQQ LEEHVKNLEQ TVSDLKVEMD KEKAKSTAKV LSLEDTLSDL KTELGKEKAK NATATDKFLL
At2M6a/1-420   ---------- ---------- ---------- ---------- ---------- ---------- ----------
At2M6b/1-420   ---------- ---------- ---------- ---------- ---------- ---------- ----------
At2M6c/1-298   ---------- ---------- ---------- ---------- ---------- ---------- ----------
At2M6d/1-298   ---------- ---------- ---------- ---------- ---------- ---------- ----------
At2M7/1-743    VQRLEERLKN LELMDLDCLK SKLELVSIKN KKADADRSRI QRLEERVKKL ELMELDDLKS KLEEVSLERK
At2M8/1-318    ---------- ---------- ---------- ---------- ---------- ---------- ----------
At2M9/1-416    ---------- ---------- ---------- ---------- ---------- ---------- ----------
At2M10/1-411   ---------- ---------- ---------- ---------- ---------- ---------- ----------
At2M11/1-309   ---------- ---------- ---------- ---------- ---------- ---------- ----------
At2M12/1-370   ---------- ---------- ---------- ---------- ---------- ---------- ----------
At2M13/1-363   ---------- ---------- ---------- ---------- ---------- ---------- ----------
At2M14/1-291   ---------- ---------- ---------- ---------- ---------- ---------- ----------
At2M15/1-379   ---------- ---------- ---------- ---------- ---------- ---------- ----------
At2M16/1-375   ---------- ---------- ---------- ---------- ---------- ---------- ----------
At2M17/1-358   EIEEDLKDLK VKCADMDALL DFLR------ ---------- ---------- ---------- ----------
At2M18/1-427   ---------- ---------- ---------- ---------- ---------- ---------- ----------
At2M19a/1-297  ---------- ---------- ---------- ---------- ---------- ---------- ----------
At2M19b/1-297  ---------- ---------- ---------- ---------- ---------- ---------- ----------
At2M20/1-299   ---------- ---------- ---------- ---------- ---------- ---------- ----------
At2M21/1-300   ---------- ---------- ---------- ---------- ---------- ---------- ----------
At2M22/1-351   ---------- ---------- ---------- ---------- ---------- ---------- ----------
At2M23a/1-350  ---------- ---------- ---------- ---------- ---------- ---------- ----------
At2M23b/1-327  ---------- ---------- ---------- ---------- ---------- ---------- ----------
At2M24/1-333   ---------- ---------- ---------- ---------- ---------- ---------- ----------
At2M25/1-352   ---------- ---------- ---------- ---------- ---------- ---------- ----------
Os2M1/1-685    ---------- ---------- ---------- ---------- ---------- ---------- ----------
```

```
                1121
At2M1/1-294     ---------- ---------- ---------- ---------- ---------- ---------- ----------
At2M2/1-396     ---------- ---------- ---------- ---------- ---------- ---------- ----------
At2M3a/1-296    ---------- ---------- ---------- ---------- ---------- ---------- ----------
At2M3b/1-296    ---------- ---------- ---------- ---------- ---------- ---------- ----------
At2M4/1-365     ---------- ---------- ---------- ---------- ---------- ---------- ----------
At2M5/1-898     LKDTYSDLKV ELEKEKAKST SAAAKVLSLK EALSDLKVEL DDQKIVNSAT TANVLSWEDD DDLFSHTNCL
At2M6a/1-420    ---------- ---------- ---------- ---------- ---------- ---------- ----------
At2M6b/1-420    ---------- ---------- ---------- ---------- ---------- ---------- ----------
At2M6c/1-298    ---------- ---------- ---------- ---------- ---------- ---------- ----------
At2M6d/1-298    ---------- ---------- ---------- ---------- ---------- ---------- ----------
At2M7/1-743     KSDDAYRSRV YQLEECFKNL ELMVLDFKVE LDKKKDKSCD DGFLLVDEFA ---------- ----------
At2M8/1-318     ---------- ---------- ---------- ---------- ---------- ---------- ----------
At2M9/1-416     ---------- ---------- ---------- ---------- ---------- ---------- ----------
At2M10/1-411    ---------- ---------- ---------- ---------- ---------- ---------- ----------
At2M11/1-309    ---------- ---------- ---------- ---------- ---------- ---------- ----------
At2M12/1-370    ---------- ---------- ---------- ---------- ---------- ---------- ----------
At2M13/1-363    ---------- ---------- ---------- ---------- ---------- ---------- ----------
At2M14/1-291    ---------- ---------- ---------- ---------- ---------- ---------- ----------
At2M15/1-379    ---------- ---------- ---------- ---------- ---------- ---------- ----------
At2M16/1-375    ---------- ---------- ---------- ---------- ---------- ---------- ----------
At2M17/1-358    ---------- ---------- ---------- ---------- ---------- ---------- ----------
At2M18/1-427    ---------- ---------- ---------- ---------- ---------- ---------- ----------
At2M19a/1-297   ---------- ---------- ---------- ---------- ---------- ---------- ----------
At2M19b/1-297   ---------- ---------- ---------- ---------- ---------- ---------- ----------
At2M20/1-299    ---------- ---------- ---------- ---------- ---------- ---------- ----------
At2M21/1-300    ---------- ---------- ---------- ---------- ---------- ---------- ----------
At2M22/1-351    ---------- ---------- ---------- ---------- ---------- ---------- ----------
At2M23a/1-350   ---------- ---------- ---------- ---------- ---------- ---------- ----------
At2M23b/1-327   ---------- ---------- ---------- ---------- ---------- ---------- ----------
At2M24/1-333    ---------- ---------- ---------- ---------- ---------- ---------- ----------
At2M25/1-352    ---------- ---------- ---------- ---------- ---------- ---------- ----------
Os2M1/1-685     ---------- ---------- ---------- ---------- ---------- ---------- ----------

                1191
At2M1/1-294     ---------- ---
At2M2/1-396     ---------- ---
At2M3a/1-296    ---------- ---
At2M3b/1-296    ---------- ---
At2M4/1-365     ---------- ---
At2M5/1-898     GIQQKTNAYK RIN
At2M6a/1-420    ---------- ---
At2M6b/1-420    ---------- ---
At2M6c/1-298    ---------- ---
At2M6d/1-298    ---------- ---
At2M7/1-743     ---------- ---
At2M8/1-318     ---------- ---
At2M9/1-416     ---------- ---
At2M10/1-411    ---------- ---
At2M11/1-309    ---------- ---
At2M12/1-370    ---------- ---
At2M13/1-363    ---------- ---
At2M14/1-291    ---------- ---
At2M15/1-379    ---------- ---
At2M16/1-375    ---------- ---
At2M17/1-358    ---------- ---
At2M18/1-427    ---------- ---
At2M19a/1-297   ---------- ---
At2M19b/1-297   ---------- ---
At2M20/1-299    ---------- ---
At2M21/1-300    ---------- ---
At2M22/1-351    ---------- ---
At2M23a/1-350   ---------- ---
At2M23b/1-327   ---------- ---
At2M24/1-333    ---------- ---
At2M25/1-352    ---------- ---
Os2M1/1-685     ---------- ---
```
