# Supplementary material for: MATH-Domain Family Shows Response toward Abiotic Stress in Arabidopsis and Rice
Source: Front Plant Sci. 2016 Jun 28;7:923. doi: 10.3389/fpls.2016.00923 (PMC4923191; doi:10.3389/fpls.2016.00923)
Supplement: Figure S4 — Multiple sequence alignment of full length sequences having MATH and BTB domains in Arabidopsis and rice. The sequence analysis was performed using Seaview (version 4) multiple sequence alignment editor (Gouy et al., 2010). [file Image4.PDF]

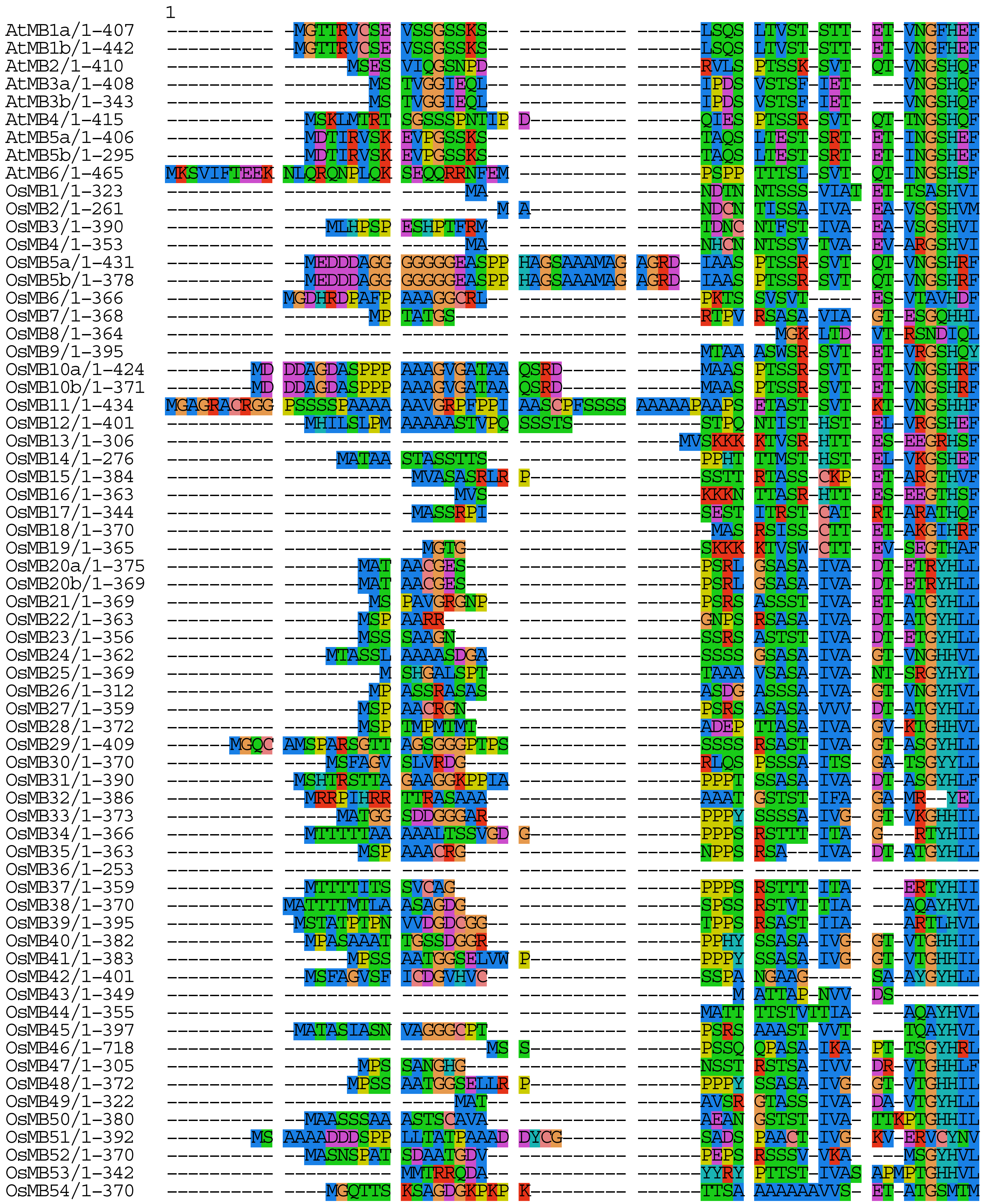
1
AtMB1a/1-407
AtMB1b/1-442
AtMB2/1-410
AtMB3a/1-408
AtMB3b/1-343
AtMB4/1-415
AtMB5a/1-406
AtMB5b/1-295
AtMB6/1-465
OsMB1/1-323
OsMB2/1-261
OsMB3/1-390
OsMB4/1-353
OsMB5a/1-431
OsMB5b/1-378
OsMB6/1-366
OsMB7/1-368
OsMB8/1-364
OsMB9/1-395
OsMB10a/1-424
OsMB10b/1-371
OsMB11/1-434
OsMB12/1-401
OsMB13/1-306
OsMB14/1-276
OsMB15/1-384
OsMB16/1-363
OsMB17/1-344
OsMB18/1-370
OsMB19/1-365
OsMB20a/1-375
OsMB20b/1-369
OsMB21/1-369
OsMB22/1-363
OsMB23/1-356
OsMB24/1-362
OsMB25/1-369
OsMB26/1-312
OsMB27/1-359
OsMB28/1-372
OsMB29/1-409
OsMB30/1-370
OsMB31/1-390
OsMB32/1-386
OsMB33/1-373
OsMB34/1-366
OsMB35/1-363
OsMB36/1-253
OsMB37/1-359
OsMB38/1-370
OsMB39/1-395
OsMB40/1-382
OsMB41/1-383
OsMB42/1-401
OsMB43/1-349
OsMB44/1-355
OsMB45/1-397
OsMB46/1-718
OsMB47/1-305
OsMB48/1-372
OsMB49/1-322
OsMB50/1-380
OsMB51/1-392
OsMB52/1-370
OsMB53/1-342
OsMB54/1-370

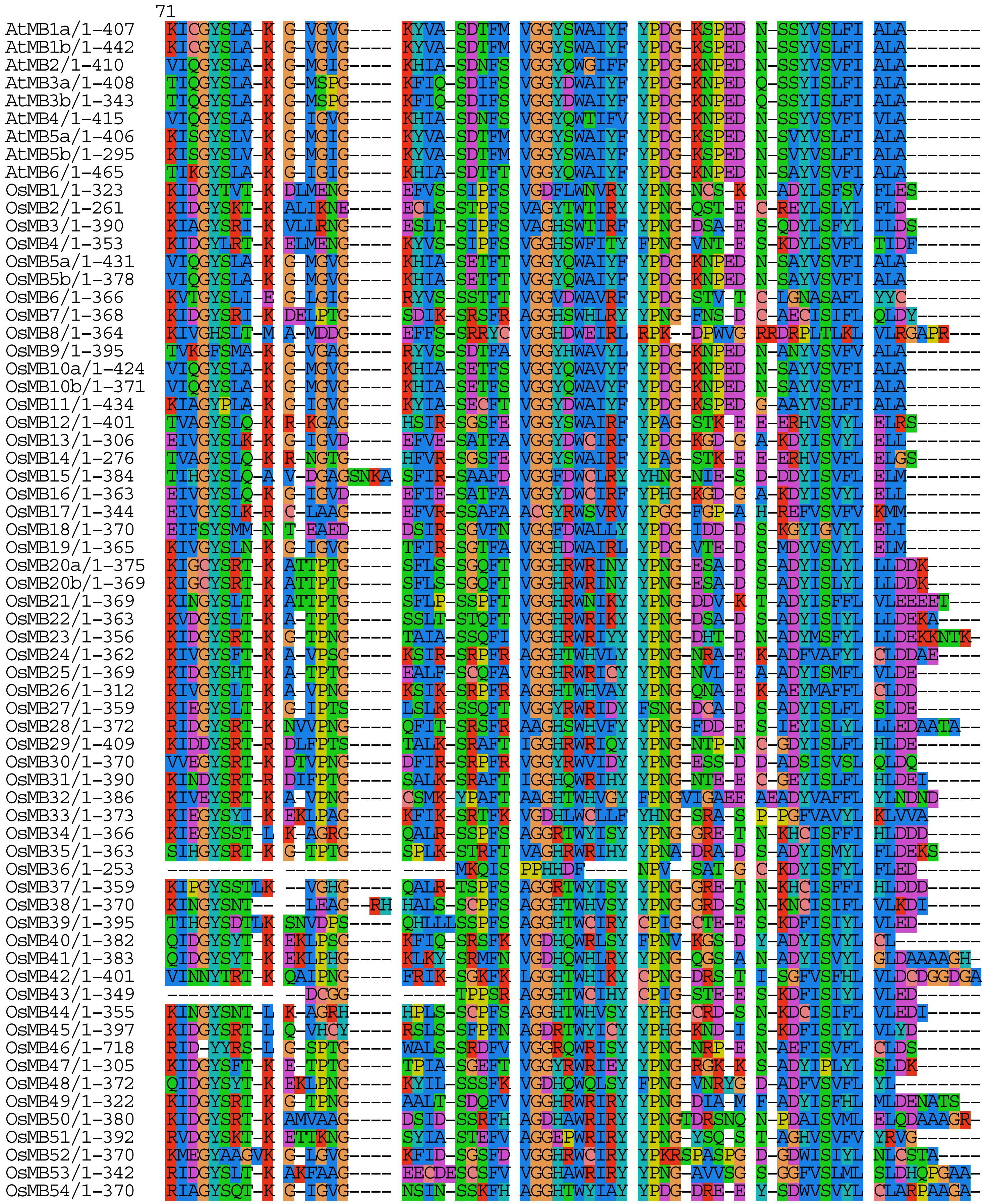
71
AtMB1a/1-407 KICGYSLA-K G-VGVG---- KYVA-SDTFM VGGYSWAIYF YPDG-KSPED N-SSYVSLFI ALA-------
AtMB1b/1-442 KICGYSLA-K G-VGVG---- KYVA-SDTFM VGGYSWAIYF YPDG-KSPED N-SSYVSLFI ALA-------
AtMB2/1-410 VIQGYSLA-K G-MGIG---- KHIA-SDNFS VGGYQWGIFF YPDG-KNPED N-SSYVSVFI ALA-------
AtMB3a/1-408 TIQGYSLA-K G-MSPG---- KFIQ-SDIFS VGGYDWAIYF YPDG-KNPED Q-SSYISLFI ALA-------
AtMB3b/1-343 TIQGYSLA-K G-MSPG---- KFIQ-SDIFS VGGYDWAIYF YPDG-KNPED Q-SSYISLFI ALA-------
AtMB4/1-415 VIQGYSLA-K G-IGVG---- KHIA-SDNFS VGGYQWTIFV YPDG-KNPED N-SSYVSVFI VLA-------
AtMB5a/1-406 KISGYSLV-K G-MGIG---- KYVA-SDTFM VGGYSWAIYF YPDG-KSPED N-SVYVSLFI ALA-------
AtMB5b/1-295 KISGYSLV-K G-MGIG---- KYVA-SDTFM VGGYSWAIYF YPDG-KSPED N-SVYVSLFI ALA-------
AtMB6/1-465 TIKGYSLA-K G-IGIG---- KHIA-SDTFT VGGYQWAIYF YPDG-KNPED N-SAYVSVFI ALA-------
OsMB1/1-323 KIDGYTVT-K DLMENG---- EFVS-SIPFS VGDFLWNVRY YPNG-NCS-K N-ADYLSFSV FLES------
OsMB2/1-261 KIDGYSKT-K ALIKNE---- ECLS-STPFS VAGYTWTIRY YPNG-QST-E C-REYLSLYL FLD-------
OsMB3/1-390 KIAGYSRI-K VLLRNG---- ESLT-SIPFS VAGHSWTIRF YPNG-DSA-E S-QDYLSFYL ILDS------
OsMB4/1-353 KIDGYLRT-K ELMENG---- KYVS-SIPFS VGGHSWFITY FPNG-VNT-E S-KDYLSVFL TIDF------
OsMB5a/1-431 VIQGYSLA-K G-MGVG---- KHIA-SETFT VGGYQWAIYF YPDG-KNPED N-SAYVSVFI ALA-------
OsMB5b/1-378 VIQGYSLA-K G-MGVG---- KHIA-SETFT VGGYQWAIYF YPDG-KNPED N-SAYVSVFI ALA-------
OsMB6/1-366 KVTGYSLI-E G-LGIG---- RYVS-SSTFT VGGVDWAVRF YPDG-STV-T C-LGNASAFL YYC-------
OsMB7/1-368 KIDGYSRI-K DELPTG---- SDIK-SRSFR AGGHSWHLRY YPNG-FNS-D C-AECISIFL QLDY------
OsMB8/1-364 KIVGHSLT-M A-MDDG---- EFFS-SRRYC VGGHDWEIRL RPK--DPWVG RRDRPLTLKL VLRGAPR---
OsMB9/1-395 TVKGFSMA-K G-VGAG---- RYVS-SDTFA VGGYHWAVYL YPDG-KNPED N-ANYVSVFV ALA-------
OsMB10a/1-424 VIQGYSLA-K G-MGVG---- KHIA-SETFS VGGYQWAVYF YPDG-KNPED N-SAYVSVFI ALA-------
OsMB10b/1-371 VIQGYSLA-K G-MGVG---- KHIA-SETFS VGGYQWAVYF YPDG-KNPED N-SAYVSVFI ALA-------
OsMB11/1-434 KIAGYPLA-K G-IGVG---- KYIA-SECFT VGGYDWAIYF YPDG-KSPED G-AAYVSLFI ALA-------
OsMB12/1-401 TVAGYSLQ-K R-KGAG---- HSIR-SGSFE VGGYSWAIRF YPAG-STK-E E-ERHVSVYL ELRS------
OsMB13/1-306 EIVGYSLK-K G-IGVD---- EFVE-SATFA VGGYDWCIRF YPDG-KGD-G A-KDYISVYL ELL-------
OsMB14/1-276 TVAGYSLQ-K R-NGTG---- HFVR-SGSFE VGGYSWAIRF YPAG-STK-E E-ERHVSVFL ELGS------
OsMB15/1-384 TIHGYSLQ-A V-DGAGSNKA SFIR-SAAFD VGGFDWCLRY YHNG-NIE-S D-DDYISVFL ELM-------
OsMB16/1-363 EIVGYSLQ-K G-IGVD---- EFIE-SATFA VGGYDWCIRF YPHG-KGD-G A-KDYISVYL ELL-------
OsMB17/1-344 EIVGYSLK-R C-LAAG---- EFVR-SSAFA ACGYRWSVRV YPGG-FGP-A H-REFVSVFV KMM-------
OsMB18/1-370 EIFSYSMM-N T-EAED---- DSIR-SGVFN VGGFDWALLY YPDG-IDD-D S-KGYIGVYL ELI-------
OsMB19/1-365 KIVGYSLN-K G-IGVG---- TFIR-SGTFA VGGHDWAIRL YPDG-VTE-D S-MDYVSVYL ELM-------
OsMB20a/1-375 KIGCYSRT-K ATTPTG---- SFLS-SGQFT VGGHRWRINY YPNG-ESA-D S-ADYISLYL LLDDK-----
OsMB20b/1-369 KIGCYSRT-K ATTPTG---- SFLS-SGQFT VGGHRWRINY YPNG-ESA-D S-ADYISLYL LLDDK-----
OsMB21/1-369 KINGYSLT-K ATTPTG---- SFLP-SSPFT VGGHRWNIKY YPNG-DDV-K T-ADYISFFL VLEEEET---
OsMB22/1-363 KVDGYSLT-K A-TPTG---- SSLT-STQFT VGGHRWRIKY YPNG-DSA-D S-ADYISIYL LLDEKA----
OsMB23/1-356 KIDGYSRT-K G-TPNG---- TAIA-SSQFI VGGHRWRIYY YPNG-DHT-D N-ADYMSFYL LLDEKKNTK-
OsMB24/1-362 KIVGYSFT-K A-VPSG---- KSIR-SRPFR AGGHTWHVLY YPNG-NRA-E K-ADFVAFYL CLDDAE----
OsMB25/1-369 KIDGYSHT-K A-TPTG---- EALF-SCQFA VGGHRWRICY YPNG-NVL-E A-ADYISMFL VLDE------
OsMB26/1-312 KIVGYSLT-K A-VPNG---- KSIK-SRPFR AGGHTWHVAY YPNG-QNA-E K-AEYMAFFL CLDD------
OsMB27/1-359 KIEGYSLT-K G-IPTS---- LSLK-SSQFT VGGYRWRIDY FSNG-DCA-D S-ADYISLFL SLDE------
OsMB28/1-372 RIDGYSRT-K NVVPNG---- QFIT-SRSFR AAGHSWHVFY YPNG-FDD-E S-IEYISLYL LLEDAATA--
OsMB29/1-409 KIDDYSRT-R DLFPTS---- TALK-SRAFT IGGHRWRIQY YPNG-NTP-N C-GDYISLFL HLDE------
OsMB30/1-370 VVEGYSRT-K DTVPNG---- DFIR-SRPFR VGGYRWVIDY YPNG-ESS-D D-ADSISVSL QLDQ------
OsMB31/1-390 KINDYSRT-R DIFPTG---- SALK-SRAFT IGGHQWRIHY YPNG-NTE-E C-GEYISLFL HLDEI-----
OsMB32/1-386 KIVEYSRT-K A-VPNG---- CSMK-YPAFT AAGHTWHVGY FPNGVIGAEE AEADYVAFFL YLNDND----
OsMB33/1-373 KIEGYSYI-K EKLPAG---- KFIK-SRTFK VGDHLWCLLF YHNG-SRA-S P-PGFVAVYL KLVVA-----
OsMB34/1-366 KIEGYSST-L K-AGRG---- QALR-SSPFS AGGRTWYISY YPNG-GRE-T N-KHCISFFI HLDDD-----
OsMB35/1-363 SIHGYSRT-K G-TPTG---- SPLK-STRFT VAGHRWRIHY YPNA-DRA-D S-ADYISMYL FLDEKS----
OsMB36/1-253 ---------- ---------- -----MKQIS PPHHDF---- NPV--SAT-G C-KDYISFYL FLED------
OsMB37/1-359 KIPGYSSTLK --VGHG---- QALR-TSPFS AGGRTWYISY YPNG-GRE-T N-KHCISFFI HLDDD-----
OsMB38/1-370 KINGYSNT-- --LEAG--RH HALS-SCPFS AGGHTWHVSY YPNG-GRD-S N-KNCISIFL VLKDI-----
OsMB39/1-395 TIHGYSDTLK SNVDPS---- QHLLLSSPFS AGGHTWCIRY CPIG-CTE-E S-KDFISIYL VLED------
OsMB40/1-382 QIDGYSYT-K EKLPSG---- KFIQ-SRSFK VGDHQWRLSY FPNV-KGS-D Y-ADYISVYL CL--------
OsMB41/1-383 QIDGYSYT-K EKLPHG---- KLKY-SRMFN VGDHQWHLRY YPNG-QGS-A N-ADYISVYL GLDAAAAGH-
OsMB42/1-401 VINNYTRT-K QAIPNG---- FRIK-SGKFK LGGHTWHIKY CPNG-DRS-T I-SGFVSFHL VLDCDGGDGA
OsMB43/1-349 ---------- --DCGG---- -----TPPSR AGGHTWCIHY CPIG-STE-E S-KDFISIYL VLED------
OsMB44/1-355 KINGYSNT-L K-AGRH---- HPLS-SCPFS AGGHTWHVSY YPHG-CRD-S N-KDCISIFL VLEDI-----
OsMB45/1-397 KIDGYSRT-L Q-VHCY---- RSLS-SFPFN AGDRTWYICY YPHG-KND-I S-KDFISIYL VLYD------
OsMB46/1-718 RID-YYRS-L G-SPTG---- WALS-SRDFV VGGRQWRISY YPNG-NRP-E N-AEFISVFL CLDS------
OsMB47/1-305 KIDGYSFT-K E-TPTG---- TPIA-SGEFT VGGYRWRIEY YPNG-RGK-K S-ADYIPLYL SLDK------
OsMB48/1-372 QIDGYSYT-K EKLPNG---- KYIL-SSSFK VGDHQWQLSY FPNG-VNRYG D-ADFVSVFL YL--------
OsMB49/1-322 KIDGYSRT-K G-TPNG---- AALT-SDQFV VGGHRWRIRY YPNG-DIA-M F-ADYISFHL MLDENATS--
OsMB50/1-380 KIDGYSRT-K AMVAAG---- DSID-SSRFH AGDHAWRIRY YPNGTDRSNQ N-PDAISVML ELQDAAAGR-
OsMB51/1-392 RVDGYSKT-K ETTKNG---- SYIA-STEFV AGGEPWRIRY YPNG-YSQ-S T-AGHVSVFV YRVG------
OsMB52/1-370 KMEGYAAGVK G-LGVG---- KFID-SGSFD VGGHRWCIRY YPKRSPASPG D-GDWISIYL NLCSTA----
OsMB53/1-342 RIDGYSLT-K AKFAAG---- EECDESCSFV VGGHAWRIRY YPNG-AVVSG S-GGFVSLML SLDHQPGAA-
OsMB54/1-370 RIAGYSQT-K G-IGVG---- NSIN-SSKFH AGGHTWYIAY YPDG-DRE-E Y-SDWVSVYL CLARPAAGA-

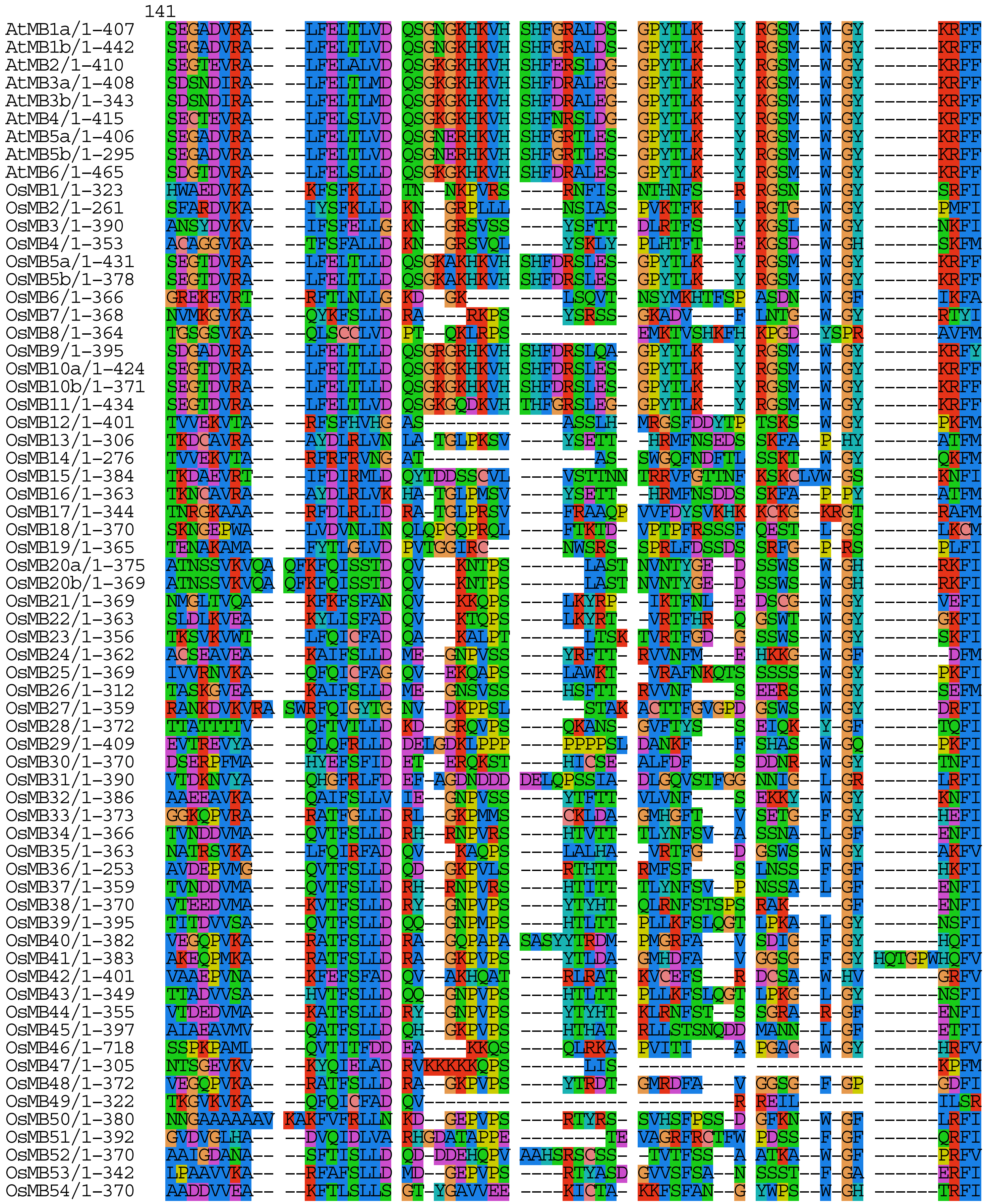
141
AtMB1a/1-407 SEGADVRA-- --LFELTLVD QSGNGKHKVH SHFGRALDS- GPYTLK---Y RGSM--W-GY -------KRFF
AtMB1b/1-442 SEGADVRA-- --LFELTLVD QSGNGKHKVH SHFGRALDS- GPYTLK---Y RGSM--W-GY -------KRFF
AtMB2/1-410 SEGTEVRA-- --LFELALVD QSGKGKHKVH SHFERSLDG- GPYTLK---Y RGSM--W-GY -------KRFF
AtMB3a/1-408 SDSNDIRA-- --LFELTLMD QSGKGKHKVH SHFDRALEG- GPYTLK---Y KGSM--W-GY -------KRFF
AtMB3b/1-343 SDSNDIRA-- --LFELTLMD QSGKGKHKVH SHFDRALEG- GPYTLK---Y KGSM--W-GY -------KRFF
AtMB4/1-415 SECTEVRA-- --LFELSLVD QSGKGKHKVH SHFNRSLDG- GPYTLK---Y RGSM--W-GY -------KRFF
AtMB5a/1-406 SEGADVRA-- --LFELTLVD QSGNERHKVH SHFGRTLES- GPYTLK---Y RGSM--W-GY -------KRFF
AtMB5b/1-295 SEGADVRA-- --LFELTLVD QSGNERHKVH SHFGRTLES- GPYTLK---Y RGSM--W-GY -------KRFF
AtMB6/1-465 SDGTDVRA-- --LFELSLLD QSGKGKHKVH SHFDRALES- GPYTLK---Y RGSM--W-GY -------KRFF
OsMB1/1-323 HWAEDVKA-- --KFSFKLLD TN--NKPVRS ----RNFIS- NTHNFS---R RGSN--W-GY -------SRFI
OsMB2/1-261 SFARDVKA-- --IYSFKLLD KN--GRPLLL ----NSIAS- PVKTFK---L RGTG--W-GY -------PMFI
OsMB3/1-390 ANSYDVKV-- --IFSFELLG KN--GRSVSS ----YSFTT- DLRTFS---Y KGSL--W-GY -------NKFI
OsMB4/1-353 ACAGGVKA-- --TFSFALLD KN--GRSVQL ----YSKLY- PLHTFT---E KGSD--W-GH -------SKFM
OsMB5a/1-431 SEGTDVRA-- --LFELTLLD QSGKAKHKVH SHFDRSLES- GPYTLK---Y RGSM--W-GY -------KRFF
OsMB5b/1-378 SEGTDVRA-- --LFELTLLD QSGKAKHKVH SHFDRSLES- GPYTLK---Y RGSM--W-GY -------KRFF
OsMB6/1-366 GREKEVRT-- --RFTLNLLG KD--GK---- ----LSQVT- NSYMKHTFSP ASDN--W-GF -------IKFA
OsMB7/1-368 NVMKGVKA-- --QYKFSLLD RA----RKPS ----YSRSS- GKADV----F LNTG--W-GY -------RTYI
OsMB8/1-364 TGSGSVKA-- --QLSCCLVD PT--QKLRPS ---------- EMKTVSHKFH KPGD--YSPR -------AVFM
OsMB9/1-395 SDGADVRA-- --LFELTLLD QSGRGRHKVH SHFDRSLQA- GPYTLK---Y RGSM--W-GY -------KRFY
OsMB10a/1-424 SEGTDVRA-- --LFELTLLD QSGKGKHKVH SHFDRSLES- GPYTLK---Y RGSM--W-GY -------KRFF
OsMB10b/1-371 SEGTDVRA-- --LFELTLLD QSGKGKHKVH SHFDRSLES- GPYTLK---Y RGSM--W-GY -------KRFF
OsMB11/1-434 SEGTDVRA-- --LFELTLVD QSGKGQDKVH THFGRSLEG- GPYTLK---Y RGSM--W-GY -------KRFF
OsMB12/1-401 TVVEKVTA-- --RFSFHVHG AS-------- ----ASSLH- MRGSFDDYTP TSKS--W-GY -------PKFM
OsMB13/1-306 TKDCAVRA-- --AYDLRLVN LA-TGLPKSV ----YSETT- -HRMFNSEDS SKFA--P-HY -------ATFM
OsMB14/1-276 TVVEKVTA-- --RFRFRVNG AT-------- -------AS- SWGQFNDFTL SSKI--W-GY -------QKFM
OsMB15/1-384 TKDAEVRT-- --IFDIRMLD QYTDDSSCVL ----VSTTNN TRRVFGTTNF KSKCLVW-GS -------KNFI
OsMB16/1-363 TKNCAVRA-- --AYDLRLVK HA-TGLPMSV ----YSETT- -HRMFNSDDS SKFA--P-PY -------ATFM
OsMB17/1-344 TNRGKAAA-- --RFDLRLID RA-TGLPRSV ----FRAAQP VVFDYSVKHK KCKG--KRGT -------RAFM
OsMB18/1-370 SKNGEPWA-- --LVDVNLIN QLQPGQPRQL ----FTKTD- VPTPFRSSSF QEST--L-GS -------LKCM
OsMB19/1-365 TENAKAMA-- --FYTLGLVD PVTGGIRC-- ----NWSRS- SPRLFDSSDS SRFG--P-RS -------PLFI
OsMB20a/1-375 ATNSSVKVQA QFKFQISSTD QV---KNTPS ------LAST NVNTYGE--D SSWS--W-GH -------RKFI
OsMB20b/1-369 ATNSSVKVQA QFKFQISSTD QV---KNTPS ------LAST NVNTYGE--D SSWS--W-GH -------RKFI
OsMB21/1-369 NMGLTVQA-- --KFKFSFAN QV---KKQPS ----LKYRP- -IKTFNL--E DSCG--W-GY -------VEFI
OsMB22/1-363 SLDLKVEA-- --KYLISFAD QV---KTQPS ----LKYRT- -VRTFHR--Q GSWT--W-GY -------GKFI
OsMB23/1-356 TKSVKVWT-- --LFQICFAD QA---KALPT ------LTSK TVRTFGD--G SSWS--W-GY -------SKFI
OsMB24/1-362 ACSEAVEA-- --KAIFSLLD ME--GNPVSS ----YRFTT- RVVNFM---E HKKG--W-GF --------DFM
OsMB25/1-369 IVVRNVKA-- --QFQICFAG QV--EKQAPS ----LAWKT- -VRAFNKQTS SSSS--W-GY -------PKFI
OsMB26/1-312 TASKGVEA-- --KAIFSLLD ME--GNSVSS ----HSFTT- RVVNF----S EERS--W-GY -------SEFM
OsMB27/1-359 RANKDVKVRA SWRFQIGYTG NV--DKPPSL ------STAK ACTTFGVGPD GSWS--W-GY -------DRFI
OsMB28/1-372 TTATTTTV-- --QFTVILLD KD--GRQVPS ----QKANS- GVFTYS---S EIQK--Y-GF -------TQFI
OsMB29/1-409 EVTREVYA-- --QLQFRLLD DELGDKLPPP ----PPPPSL DANKF----F SHAS--W-GQ -------PKFI
OsMB30/1-370 DSERPFMA-- --HYEFSFID ET--ERQKST ----HICSE- ALFDF----S DDNR--W-GY -------TNFI
OsMB31/1-390 VTDKNVYA-- --QHGFRLFD EF-AGDNDDD DELQPSSIA- DLGQVSTFGG NNIG--L-GR -------LRFI
OsMB32/1-386 AAEEAVKA-- --QAIFSLLV IE--GNPVSS ----YTFTT- VLVNF----S EKKY--W-GY -------KNFI
OsMB33/1-373 GGKQPVRA-- --RATFGLLD RL--GKPMMS ----CKLDA- GMHGFT---V SETG--F-GY -------HEFI
OsMB34/1-366 TVNDDVMA-- --QVTFSLLD RH--RNPVRS ----HTVTT- TLYNFSV--A SSNA--L-GF -------ENFI
OsMB35/1-363 NATRSVKA-- --LFQIRFAD QV---KAQPS ----LALHA- -VRTFG---D GSWS--W-GY -------AKFV
OsMB36/1-253 AVDEPVMG-- --QVTFSLLD QD--GKPVLS ----RTHTT- RMFSF----S LNSS--F-GF -------HKFI
OsMB37/1-359 TVNDDVMA-- --QVTFSLLD RH--RNPVRS ----HTITT- TLYNFSV--P NSSA--L-GF -------ENFI
OsMB38/1-370 VTEEDVMA-- --KVTFSLLD RY--GNPVPS ----YTYHT- QLRNFSTSPS RAK-----GF -------ENFI
OsMB39/1-395 TITDVVSA-- --QVTFSLLD QQ--GNPMPS ----HTLTT- PLLKFSLQGT LPKA--L-GY -------NSFI
OsMB40/1-382 VEGQPVKA-- --RATFSLLD RA--GQPAPA SASYYTRDM- PMGRFA---V SDIG--F-GY -------HQFI
OsMB41/1-383 AKEQPMKA-- --RATFSLLD RA--GKPVPS ----YTLDA- GMHDFA---V GGSG--F-GY HQTGPWHQFV
OsMB42/1-401 VAAEPVNA-- --KFEFSFAD QV--AKHQAT ----RLRAT- KVCEFS---R DCSA--W-HV -------GRFV
OsMB43/1-349 TTADVVSA-- --HVTFSLLD QQ--GNPVPS ----HTLTT- PLLKFSLQGT LPKG--L-GY -------NSFI
OsMB44/1-355 VTDEDVMA-- --KATFSLLD RY--GNPVPS ----YTYHT- KLRNFST--S SGRA--R-GF -------ENFI
OsMB45/1-397 AIAEAVMV-- --QATFSLLD QH--GKPVPS ----HTHAT- RLLSTSNQDD MANN--L-GF -------ETFI
OsMB46/1-718 SSPKPAML-- --QVTITFDD EA----KKQS ----QLRKA- PVITI----A PGAC--W-GY -------HRFV
OsMB47/1-305 NTSGEVKV-- --KYQIELAD RVKKKKKQPS ------LIS- ---------- ---------- -------KPFM
OsMB48/1-372 VEGQPVKA-- --RATFSLLD RA--GKPVPS ----YTRDT- GMRDFA---V GGSG--F-GP -------GDFI
OsMB49/1-322 TKGVKVKA-- --QFQICFAD QV-------- ---------- ---------R REIL------ -------ILSR
OsMB50/1-380 NNGAAAAAAV KAKFVFRLLN KD--GEPVPS ----RTYRS- SVHSFPSS-D GFKN--W-GF -------LRFI
OsMB51/1-392 GVDVGLHA-- --DVQIDLVA RHGDATAPPE --------TE VAGRFRCTFW PDSS--F-GF -------QRFI
OsMB52/1-370 AAIGDANA-- --SFTISLLD QD-DDEHQPV AAHSRSCSS- -TVTFSS--A ATKA--W-GF -------PRFV
OsMB53/1-342 LPAAVVKA-- --RFAFSLLD MD--GEPVPS ----RTYASD GVVSFSA--N SSST--F-GA -------ERFI
OsMB54/1-370 AADDVVEA-- --KFTLSLLS GT-YGAVVEE ----KICTA- KKFSFAN--G YWPS--W-GH -------TRFI

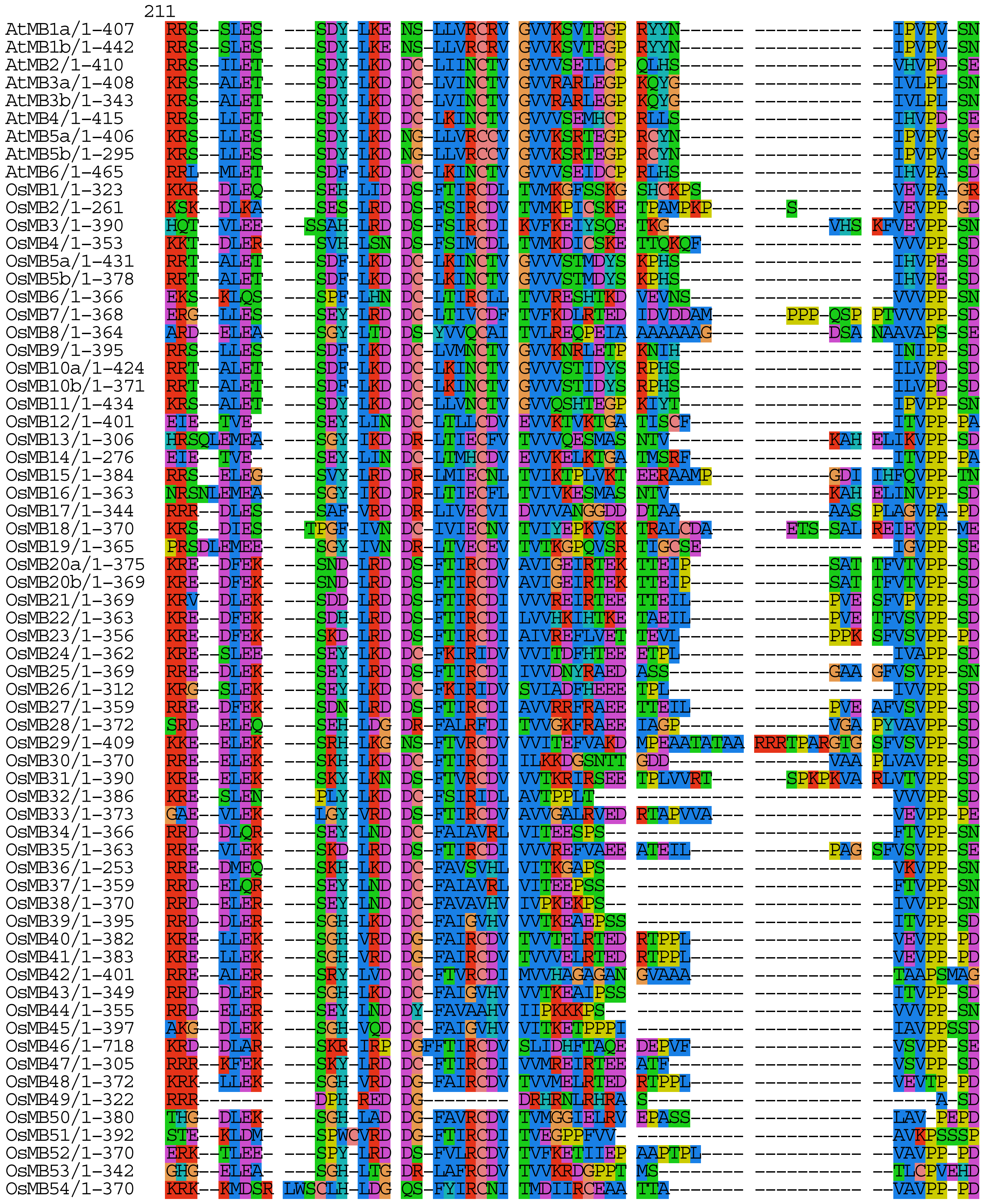
211
AtMB1a/1-407
AtMB1b/1-442
AtMB2/1-410
AtMB3a/1-408
AtMB3b/1-343
AtMB4/1-415
AtMB5a/1-406
AtMB5b/1-295
AtMB6/1-465
OsMB1/1-323
OsMB2/1-261
OsMB3/1-390
OsMB4/1-353
OsMB5a/1-431
OsMB5b/1-378
OsMB6/1-366
OsMB7/1-368
OsMB8/1-364
OsMB9/1-395
OsMB10a/1-424
OsMB10b/1-371
OsMB11/1-434
OsMB12/1-401
OsMB13/1-306
OsMB14/1-276
OsMB15/1-384
OsMB16/1-363
OsMB17/1-344
OsMB18/1-370
OsMB19/1-365
OsMB20a/1-375
OsMB20b/1-369
OsMB21/1-369
OsMB22/1-363
OsMB23/1-356
OsMB24/1-362
OsMB25/1-369
OsMB26/1-312
OsMB27/1-359
OsMB28/1-372
OsMB29/1-409
OsMB30/1-370
OsMB31/1-390
OsMB32/1-386
OsMB33/1-373
OsMB34/1-366
OsMB35/1-363
OsMB36/1-253
OsMB37/1-359
OsMB38/1-370
OsMB39/1-395
OsMB40/1-382
OsMB41/1-383
OsMB42/1-401
OsMB43/1-349
OsMB44/1-355
OsMB45/1-397
OsMB46/1-718
OsMB47/1-305
OsMB48/1-372
OsMB49/1-322
OsMB50/1-380
OsMB51/1-392
OsMB52/1-370
OsMB53/1-342
OsMB54/1-370

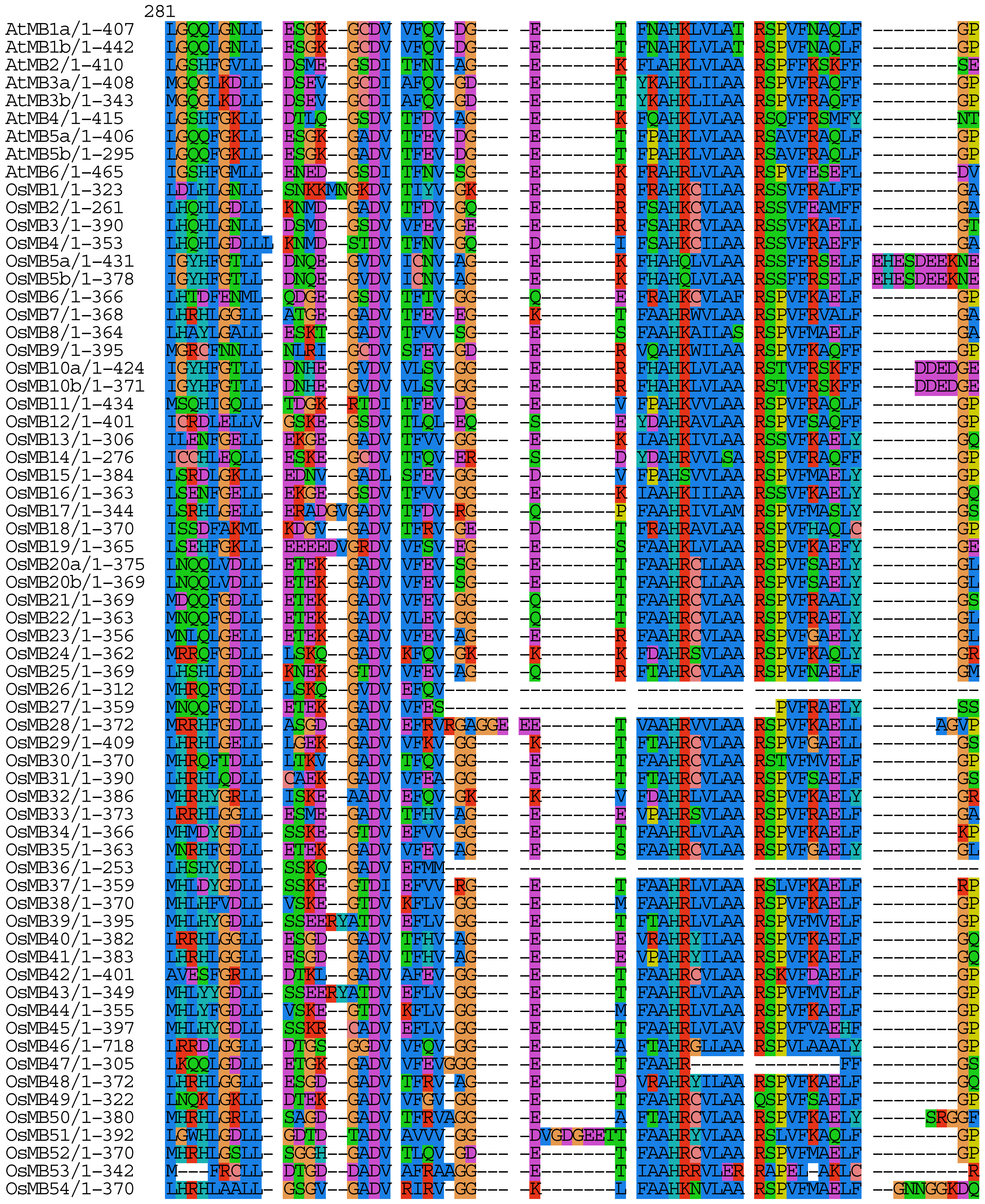
281
AtMB1a/1-407 LGQQLGNLL- ESGK--GCDV VFQV-DG--- -E-------T FNAHKLVLAT RSPVFNAQLF ---------GP
AtMB1b/1-442 LGQQLGNLL- ESGK--GCDV VFQV-DG--- -E-------T FNAHKLVLAT RSPVFNAQLF ---------GP
AtMB2/1-410 LGSHFGVLL- DSME--GSDI TFNI-AG--- -E-------K FLAHKLVLAA RSPFFKSKFF ---------SE
AtMB3a/1-408 MGQGLKDLL- DSEV--GCDI AFQV-GD--- -E-------T YKAHKLILAA RSPVFRAQFF ---------GP
AtMB3b/1-343 MGQGLKDLL- DSEV--GCDI AFQV-GD--- -E-------T YKAHKLILAA RSPVFRAQFF ---------GP
AtMB4/1-415 LGSHFGKLL- DTLQ--GSDV TFDV-AG--- -E-------K FQAHKLVLAA RSQFFRSMFY ---------NT
AtMB5a/1-406 LGQQFGKLL- ESGK--GADV TFEV-DG--- -E-------T FPAHKLVLAA RSAVFRAQLF ---------GP
AtMB5b/1-295 LGQQFGKLL- ESGK--GADV TFEV-DG--- -E-------T FPAHKLVLAA RSAVFRAQLF ---------GP
AtMB6/1-465 IGSHFGMLL- ENED--GSDI TFNV-SG--- -E-------K FRAHRLVLAA RSPVFESEFL ---------DV
OsMB1/1-323 LDLHLGNLL- SNKKMNGKDV TIYV-GK--- -E-------R FRAHKCILAA RSSVFRALFF ---------GA
OsMB2/1-261 LHQHLGDLL- KNMD--GADV TFDV-GQ--- -E-------R FSAHKCVLAA RSSVFEAMFF ---------GA
OsMB3/1-390 LHQHLGNLL- DSMD--GSDV VFEV-GE--- -E-------R FSAHRCVLAA RSSVFKAELL ---------GT
OsMB4/1-353 LHQHLGDLLL KNMD--STDV TFNV-GQ--- -D-------I FSAHKCILAA RSSVFRAEFF ---------GA
OsMB5a/1-431 IGYHFGTLL- DNQE--GVDV ICNV-AG--- -E-------K FHAHQLVLAA RSSFFRSELF EHESDEEKNE
OsMB5b/1-378 IGYHFGTLL- DNQE--GVDV ICNV-AG--- -E-------K FHAHQLVLAA RSSFFRSELF EHESDEEKNE
OsMB6/1-366 LHTDFENML- QDGE--GSDV TFIV-GG--- -Q-------E FRAHKCVLAF RSPVFKAELF ---------GP
OsMB7/1-368 LHRHLGGLL- ATGE--GADV TFEV-EG--- -K-------T FAAHRWVLAA RSPVFRVALF ---------GA
OsMB8/1-364 LHAYLGALL- ESKT--GADV TFVV-SG--- -E-------S FAAHKAILAS RSPVFMAELF ---------GA
OsMB9/1-395 MGRCFNNLL- NLRI--GCDV SFEV-GD--- -E-------R VQAHKWILAA RSPVFKAQFF ---------GP
OsMB10a/1-424 IGYHFGTLL- DNHE--GVDV VLSV-GG--- -E-------R FHAHKLVLAA RSTVFRSKFF -----DDEDGE
OsMB10b/1-371 IGYHFGTLL- DNHE--GVDV VLSV-GG--- -E-------R FHAHKLVLAA RSTVFRSKFF -----DDEDGE
OsMB11/1-434 MSQHIGQLL- TDGK--RTDI TFEV-DG--- -E-------V FPAHKVVLAA RSPVFRAQLF ---------GP
OsMB12/1-401 ICRDLELLV- GSKE--GSDV TLQL-EQ--- -S-------E YDAHRAVLAA RSPVFSAQFF ---------GP
OsMB13/1-306 ILENFGELL- EKGE--GADV TFVV-GG--- -E-------K IAAHKIVLAA RSSVFKAELY ---------GQ
OsMB14/1-276 ICCHLEQLL- ESKE--GCDV TFQV-ER--- -S-------D YDAHRVVLSA RSPVFRAQFF ---------GP
OsMB15/1-384 LSRDLGKLL- EDNV--GADL SFEV-GG--- -D-------V FPAHSVVLAA RSPVFMAELY ---------GP
OsMB16/1-363 LSENFGELL- EKGE--GSDV TFVV-GG--- -E-------K IAAHKIILAA RSSVFKAELY ---------GQ
OsMB17/1-344 LSRHLGELL- ERADGVGADV TFDV-RG--- -Q-------P FAAHRIVLAM RSPVFMASLY ---------GS
OsMB18/1-370 ISSDFAKML- KDGV--GADV TFRV-GE--- -D-------T FRAHRAVLAA RSPVFHAQLC ---------GP
OsMB19/1-365 LSEHFGKLL- EEEEDVGRDV VFSV-EG--- -E-------S FAAHKLVLAA RSPVFKAEFY ---------GE
OsMB20a/1-375 LNQQLVDLL- ETEK--GADV VFEV-SG--- -E-------T FAAHRCLLAA RSPVFSAELY ---------GL
OsMB20b/1-369 LNQQLVDLL- ETEK--GADV VFEV-SG--- -E-------T FAAHRCLLAA RSPVFSAELY ---------GL
OsMB21/1-369 MDQQFGDLL- ETEK--GADV VFEV-GG--- -Q-------T FAAHRCVLAA RSPVFRAALY ---------GS
OsMB22/1-363 MNQQFGDLL- ETEK--GADV VLEV-GG--- -Q-------T FAAHRCVLAA RSPVFRAELY ---------GL
OsMB23/1-356 MNLQLGELL- ETEK--GADV VFEV-AG--- -E-------R FAAHRCVLAA RSPVFGAELY ---------GL
OsMB24/1-362 MRRQFGDLL- LSKQ--GADV KFQV-GK--- -K-------K FDAHRSVLAA RSPVFKAQLY ---------GR
OsMB25/1-369 LHSHLGDLL- KNEK--GTDV VFEV-AG--- -Q-------R FTAHRCVLAA RSPVFNAELF ---------GM
OsMB26/1-312 MHRQFGDLL- LSKQ--GVDV EFQV
OsMB27/1-359 MNQQFGDLL- ETEK--GADV VFES------ ---------- ---------- --PVFRAELY ---------SS
OsMB28/1-372 MRRHFGDLL- ASGD--GADV EFRVRGAGGE EE-------T VAAHRVVLAA RSPVFKAELL -------AGVP
OsMB29/1-409 LHRHLGELL- LGEK--GADV VFKV-GG--- -K-------T FTAHRCVLAA RSPVFGAELL ---------GS
OsMB30/1-370 MHRQFTDLL- LTKV--GADV TFQV-GG--- -E-------T FAAHRCVLAA RSTVFMVELF ---------GP
OsMB31/1-390 LHRHLQDLL- CAEK--GADV VFEA-GG--- -E-------T FTAHRCVLAA RSPVFSAELF ---------GS
OsMB32/1-386 MHRHYGRLL- ISKE--AADV EFQV-GK--- -K-------V FDAHRLVLAA RSPVFKAELY ---------GR
OsMB33/1-373 LRRHLGGLL- ESME--GADV TFHV-AG--- -E-------E VPAHRSVLAA RSPVFRAELF ---------GA
OsMB34/1-366 MHMDYGDLL- SSKE--GTDV EFVV-GG--- -E-------T FAAHRLVLAA RSPVFKAELF ---------KP
OsMB35/1-363 MNRHFGDLL- ETEK--GADV VFEV-AG--- -E-------S FAAHRCVLAA RSPVFGAELY ---------GL
OsMB36/1-253 LHSHYGDLL- SSKQ--GADV EFMM------ ---------- ---------- ---------- ----------
OsMB37/1-359 MHLDYGDLL- SSKE--GTDI EFVV-RG--- -E-------T FAAHRLVLAA RSLVFKAELF ---------RP
OsMB38/1-370 MHLHFVDLL- VSKE--GTDV KFLV-GG--- -E-------M FAAHRLVLAA RSPVFKAELF ---------GP
OsMB39/1-395 MHLHYGDLL- SSEERYATDV EFLV-GG--- -E-------T FTAHRLVLAA RSPVFMVELF ---------GP
OsMB40/1-382 LRRHLGGLL- ESGD--GADV TFHV-AG--- -E-------E VRAHRYILAA RSPVFKAELF ---------GQ
OsMB41/1-383 LHRHLGGLL- ESGD--GADV TFHV-AG--- -E-------E VPAHRYILAA RSPVFKAELF ---------GQ
OsMB42/1-401 AVESFGRLL- DTKL--GADV AFEV-GG--- -E-------T FAAHRCVLAA RSKVFDAELF ---------GP
OsMB43/1-349 MHLYYGDLL- SSEERYATDV EFLV-GG--- -E-------T FAAHRLVLAA RSPVFMVELF ---------GP
OsMB44/1-355 MHLYFGDLL- VSKE--GTDV KFLV-GG--- -E-------M FAAHRLVLAA RSPVFKAELF ---------GP
OsMB45/1-397 MHLHYGDLL- SSKR--CADV EFLV-GG--- -E-------T FAAHRLVLAV RSPVFVAEHF ---------GP
OsMB46/1-718 LRRDLGGLL- DTGS--GGDV VFQV-GG--- -E-------A FTAHRGLLAA RSPVLAAALY ---------GP
OsMB47/1-305 LKQQLGDLL- ETGK--GADV VFEVGGG--- -E-------T FAAHR----- --------FF ---------GS
OsMB48/1-372 LHRHLGGLL- ESGD--GADV TFRV-AG--- -E-------D VRAHRYILAA RSPVFKAELF ---------GQ
OsMB49/1-322 LNQKLGKLL- DTEK--GADV VFGV-GG--- -E-------T FAAHRCVLAA QSPVFSAELF ---------GP
OsMB50/1-380 MHRHLGRLL- SAGD--GADV TFRVAGG--- -E-------A FTAHRCVLAA RSPVFKAELY ------SRGGF
OsMB51/1-392 LGWHLGDLL- GDTD--TADV AVVV-GG--- -DVGDGEETT FAAHRYVLAA RSLVFKAQLF ---------GP
OsMB52/1-370 MHRHLGSLL- SGGH--GADV TLQV-GD--- -E-------T FAAHRCVLAA RSPVFMAELF ---------GP
OsMB53/1-342 M---FRCLL- DTGD--DADV AFRAAGG--- -E-------T IAAHRRVLER RAPEL-AKLC ----------R
OsMB54/1-370 LHRHLAALL- GSGV--GADV RIRV-GG--- -K-------L FAAHKNVLAA RSPVFMAELF --GNNGGKDQ

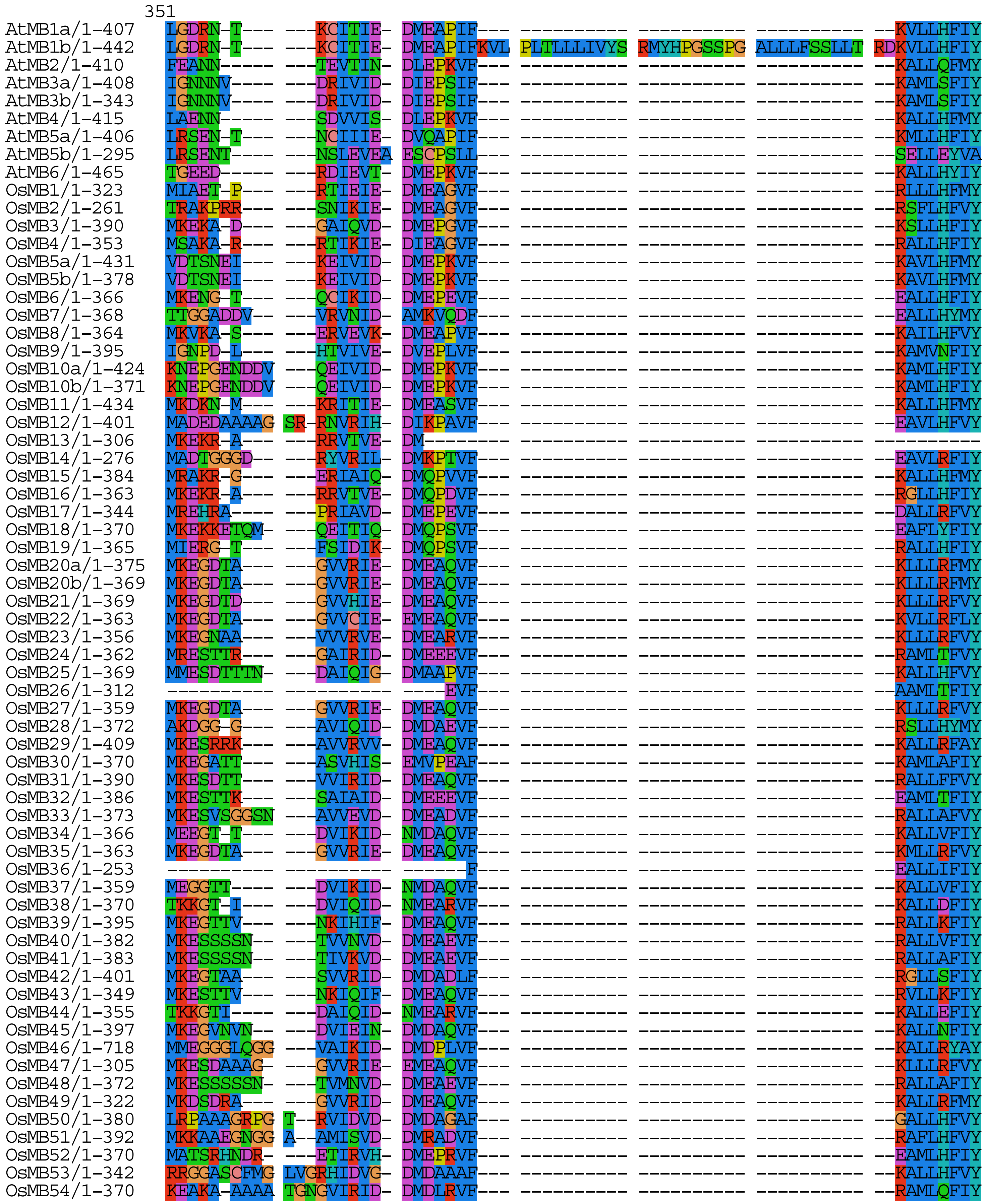
351
AtMB1a/1-407 LGDRN-T--- ---KCITIE- DMEAPIF--- ---------- ---------- ---------- --KVLLHFIY
AtMB1b/1-442 LGDRN-T--- ---KCITIE- DMEAPIFKVL PLTLLLIVYS RMYHPGSSPG ALLLFSSLLT RDKVLLHFIY
AtMB2/1-410 FEANN----- ---TEVTIN- DLEPKVF--- ---------- ---------- ---------- --KALLQFMY
AtMB3a/1-408 IGNNNV---- ---DRIVID- DIEPSIF--- ---------- ---------- ---------- --KAMLSFIY
AtMB3b/1-343 IGNNNV---- ---DRIVID- DIEPSIF--- ---------- ---------- ---------- --KAMLSFIY
AtMB4/1-415 LAENN----- ---SDVVIS- DLEPKVF--- ---------- ---------- ---------- --KALLHFMY
AtMB5a/1-406 LRSEN-T--- ---NCIIIE- DVQAPIF--- ---------- ---------- ---------- --KMLLHFIY
AtMB5b/1-295 LRSENT---- ---NSLEVEA ESCPSLL--- ---------- ---------- ---------- --SELLEYVA
AtMB6/1-465 TGEED----- ---RDIEVT- DMEPKVF--- ---------- ---------- ---------- --KALLHYIY
OsMB1/1-323 MIAET-P--- ---RTIEIE- DMEAGVF--- ---------- ---------- ---------- --RLLLHFMY
OsMB2/1-261 TRAKPRR--- ---SNIKIE- DMEAGVF--- ---------- ---------- ---------- --RSFLHFVY
OsMB3/1-390 MKEKA-D--- ---GAIQVD- DMEPGVF--- ---------- ---------- ---------- --KSLLHFIY
OsMB4/1-353 MSAKA-R--- ---RTIKIE- DIEAGVF--- ---------- ---------- ---------- --RALLHFIY
OsMB5a/1-431 VDTSNEI--- ---KEIVID- DMEPKVF--- ---------- ---------- ---------- --KAVLHFMY
OsMB5b/1-378 VDTSNEI--- ---KEIVID- DMEPKVF--- ---------- ---------- ---------- --KAVLHFMY
OsMB6/1-366 MKENG-T--- ---QCIKID- DMEPEVF--- ---------- ---------- ---------- --EALLHFIY
OsMB7/1-368 TTGGADDV-- ---VRVNID- AMKVQDF--- ---------- ---------- ---------- --EALLHYMY
OsMB8/1-364 MKVKA-S--- ---ERVEVK- DMEAPVF--- ---------- ---------- ---------- --KAILHFVY
OsMB9/1-395 IGNPD-L--- ---HTVIVE- DVEPLVF--- ---------- ---------- ---------- --KAMVNFIY
OsMB10a/1-424 KNEPGENDDV ---QEIVID- DMEPKVF--- ---------- ---------- ---------- --KAMLHFIY
OsMB10b/1-371 KNEPGENDDV ---QEIVID- DMEPKVF--- ---------- ---------- ---------- --KAMLHFIY
OsMB11/1-434 MKDKN-M--- ---KRITIE- DMEASVF--- ---------- ---------- ---------- --KALLHFMY
OsMB12/1-401 MADEDAAAAG SR-RNVRIH- DIKPAVF--- ---------- ---------- ---------- --EAVLHFVY
OsMB13/1-306 MKEKR-A--- ---RRVTVE- DM-------- ---------- ---------- ---------- ----------
OsMB14/1-276 MADTGGGD-- ---RYVRIL- DMKPTVF--- ---------- ---------- ---------- --EAVLRFIY
OsMB15/1-384 MRAKR-G--- ---ERIAIQ- DMQPVVF--- ---------- ---------- ---------- --KALLHFMY
OsMB16/1-363 MKEKR-A--- ---RRVTVE- DMQPDVF--- ---------- ---------- ---------- --RGLLHFIY
OsMB17/1-344 MREHRA---- ---PRIAVD- DMEPEVF--- ---------- ---------- ---------- --DALLRFVY
OsMB18/1-370 MKEKKETQM- ---QEITIQ- DMQPSVF--- ---------- ---------- ---------- --EAFLYFIY
OsMB19/1-365 MIERG-T--- ---FSIDIK- DMQPSVF--- ---------- ---------- ---------- --RALLHFIY
OsMB20a/1-375 MKEGDTA--- ---GVVRIE- DMEAQVF--- ---------- ---------- ---------- --KLLLRFMY
OsMB20b/1-369 MKEGDTA--- ---GVVRIE- DMEAQVF--- ---------- ---------- ---------- --KLLLRFMY
OsMB21/1-369 MKEGDTD--- ---GVVHIE- DMEAQVF--- ---------- ---------- ---------- --KLLLRFVY
OsMB22/1-363 MKEGDTA--- ---GVVCIE- EMEAQVF--- ---------- ---------- ---------- --KVLLRFLY
OsMB23/1-356 MKEGNAA--- ---VVVRVE- DMEARVF--- ---------- ---------- ---------- --KLLLRFVY
OsMB24/1-362 MRESTTR--- ---GAIRID- DMEEEVF--- ---------- ---------- ---------- --RAMLTFVY
OsMB25/1-369 MMESDTTTN- ---DAIQIG- DMAAPVF--- ---------- ---------- ---------- --KALLHFVY
OsMB26/1-312 ---------- ---------- ----EVF--- ---------- ---------- ---------- --AAMLTFIY
OsMB27/1-359 MKEGDTA--- ---GVVRIE- DMEAQVF--- ---------- ---------- ---------- --KLLLRFVY
OsMB28/1-372 AKDGG-G--- ---AVIQID- DMDAEVF--- ---------- ---------- ---------- --RSLLHYMY
OsMB29/1-409 MKESRRK--- ---AVVRVV- DMEAQVF--- ---------- ---------- ---------- --KALLRFAY
OsMB30/1-370 MKEGATT--- ---ASVHIS- EMVPEAF--- ---------- ---------- ---------- --KAMLAFIY
OsMB31/1-390 MKESDTT--- ---VVIRID- DMEAQVF--- ---------- ---------- ---------- --RALLFFVY
OsMB32/1-386 MKESTTK--- ---SAIAID- DMEEEVF--- ---------- ---------- ---------- --EAMLTFIY
OsMB33/1-373 MKESVSGGSN ---AVVEVD- DMEADVF--- ---------- ---------- ---------- --RALLAFVY
OsMB34/1-366 MEEGT-T--- ---DVIKID- NMDAQVF--- ---------- ---------- ---------- --KALLVFIY
OsMB35/1-363 MKEGDTA--- ---GVVRIE- DMEAQVF--- ---------- ---------- ---------- --KMLLRFVY
OsMB36/1-253 ---------- ---------- ------F--- ---------- ---------- ---------- --EALLIFIY
OsMB37/1-359 MEGGTT---- ---DVIKID- NMDAQVF--- ---------- ---------- ---------- --KALLVFIY
OsMB38/1-370 TKKGT-I--- ---DVIQID- NMEARVF--- ---------- ---------- ---------- --KALLDFIY
OsMB39/1-395 MKEGTTV--- ---NKIHIF- DMEAQVF--- ---------- ---------- ---------- --RALLKFIY
OsMB40/1-382 MKESSSSN-- ---TVVNVD- DMEAEVF--- ---------- ---------- ---------- --RALLVFIY
OsMB41/1-383 MKESSSSN-- ---TIVKVD- DMEAEVF--- ---------- ---------- ---------- --RALLAFIY
OsMB42/1-401 MKEGTAA--- ---SVVRID- DMDADLF--- ---------- ---------- ---------- --RGLLSFIY
OsMB43/1-349 MKESTTV--- ---NKIQIF- DMEAQVF--- ---------- ---------- ---------- --RVLLKFIY
OsMB44/1-355 TKKGTI---- ---DAIQID- NMEARVF--- ---------- ---------- ---------- --KALLEFIY
OsMB45/1-397 MKEGVNVN-- ---DVIEIN- DMDAQVF--- ---------- ---------- ---------- --KALLNFIY
OsMB46/1-718 MMEGGGLQGG ---VAIKID- DMDPLVF--- ---------- ---------- ---------- --KALLRYAY
OsMB47/1-305 MKESDAAAG- ---GVVRIE- EMEAQVF--- ---------- ---------- ---------- --KLLLRFVY
OsMB48/1-372 MKESSSSSN- ---TVMNVD- DMEAEVF--- ---------- ---------- ---------- --RALLAFIY
OsMB49/1-322 MKDSDRA--- ---GVVRID- DMEAQVF--- ---------- ---------- ---------- --KALLRFMY
OsMB50/1-380 LRPAAAGRPG T--RVIDVD- DMDAGAF--- ---------- ---------- ---------- --GALLHFVY
OsMB51/1-392 MKKAAEGNGG A--AMISVD- DMRADVF--- ---------- ---------- ---------- --RAFLHFVY
OsMB52/1-370 MATSRHNDR- ---ETIRVH- DMEPRVF--- ---------- ---------- ---------- --EAMLHFIY
OsMB53/1-342 RRGGASCFMG LVGRHIDVG- DMDAAAF--- ---------- ---------- ---------- --KALLHFVY
OsMB54/1-370 KEAKA-AAAA TGNGVIRID- DMDLRVF--- ---------- ---------- ---------- --RAMLQFIY

```
             421
AtMB1a/1-407   WDEL-PDMQE ---------- ---------- LIGTDSTLAS TLVAQHLLAA ADRYAL--ER LKAICESK-L
AtMB1b/1-442   WDEL-PDMQE ---------- ---------- LIGTDSTLAS TLVAQHLLAA ADRYAL--ER LKAICESK-L
AtMB2/1-410    KDSL-PEDVE ---------- ----PATAHT FERLKLSEIY ETLIVKVLAA ADKYDL--IR LRLLCESH-I
AtMB3a/1-408   TDVL-PNVHE ---------- ---------- ITGSTSASSF TNMIQHLLAA ADLYDL--AR LKILCEVL-L
AtMB3b/1-343   TDVL-PNVHE ---------- ---------- ITGSTSASSF TNMIQHLLAA ADLYDL--AR LKILCEVL-L
AtMB4/1-415    KDSL-PGDVE ---------- ----PLTAHS FDLLRPSEID DTLIVKLLAA AEMYNL--SR LRLLCESH-I
AtMB5a/1-406   WDEM-PDMQD ---------- ---------- LIGTDLKWAS TLVAQHLLAA ADRYAL--ER LRTICESK-L
AtMB5b/1-295   R--------- ---------- ---------- ---------- --LSEHSLTS SG------HR KELFADGCDL
AtMB6/1-465    KDAL-IEDAE SSSSSG---- ---------- --SSVGPSAS DTLAAKLLGA ADKYKL--PR LSLMCESV-L
OsMB1/1-323    NDSL-PETWS ---------- ---------- --------QD AMMAQHLLVA ADWYNV--GR LKLICEEK-L
OsMB2/1-261    TDLL-PDTSQ ---------- ---------- ---------D VVMAQ----- ---------- ----------
OsMB3/1-390    TDSL-DTMAQ ---------- -------EDQ SRDEASEEED LVMAQHLLVA ADRYNV--ER LKLICEEK-L
OsMB4/1-353    TDSL-PETAQ ---------- ---------- ---------N IVMAQHLVVA ADRYNV--GR LKLICEEK-L
OsMB5a/1-431   RDNL-VGDDE ---------- ---------L SASSSDCSIF DTLAGKLLAA ADRYEL--PR LRLLCESY-L
OsMB5b/1-378   RDNL-VGDDE ---------- ---------L SASSSDCSIF DTLAGKLLAA ADRYEL--PR LRLLCESY-L
OsMB6/1-366    TDRL-PDSCR ---------- ---------- -------DGK AAAMQHLLVA ADRYGV--DR LRLICERR-L
OsMB7/1-368    TDSL-PEMKG ---------- ---------- -------GEA AAMLPDLVAA ANRYKM--ER LRLVCEHK-L
OsMB8/1-364    TDTV-PELDH ---------- --------RD GEETEAASTA TAMAQHLLAG ADRYGL--ER LKLICESK-L
OsMB9/1-395    SDEL-PSIHE LA-------- ---------- --GSVSTWTS TVVVQHLLAA ADRYGL--DR LRLLCEEK-L
OsMB10a/1-424  RDTL-VDDNE ---------- ---------L GGSSSEGSIF DTLAAKLLAA ADKYDL--AR LRLLCESY-L
OsMB10b/1-371  RDTL-VDDNE ---------- ---------L GGSSSEGSIF DTLAAKLLAA ADKYDL--AR LRLLCESY-L
OsMB11/1-434   WDEL-PDIEE ---------- ---------- LTGLNTTWVS TLMAQHLLAA ADRYAL--ER LKLLCELK-L
OsMB12/1-401   TDTL-PPATT ----SWSASH RDKRPKLSDV AAASCSEEEV RVMIGERLAA ADRFDL--ER MRLLCEDA-L
OsMB13/1-306   ---------- ---------- ---------- ---------- ---------- ---YAM--DR MKLQCESI-L
OsMB14/1-276   TDRL-PPVED ---------- ---------- ---------- ---------- ---------- ----------
OsMB15/1-384   TDSFSPAIND ---------- ---------- ---DLSRDER QELAKHLLVA ADRYAV--EG LKTICEKA-L
OsMB16/1-363   TDSL-PDMDD ---------- ---------- ----LSDDDY YEMIRLLLVA ADRYAM--DR MKLQCESI-L
OsMB17/1-344   SDTL-ALPGD ---------- ---------- ----LGEGEY KEMVRQLLEA ADRYAM--DR LRVICELI-L
OsMB18/1-370   TDCV-PRMDD ---------- ---------- ----LGNGEK MHLMMHLLKA GDRYGL--ER LRIMCERF-L
OsMB19/1-365   TDVL-PADIG ---------- ---------- ---DLEGDDY VEFIRHLVVA ADRYAM--DR LKLMCQSI-L
OsMB20a/1-375  TDSL-PKMEE ---------- ---------- ---------E DVMWQHLLVA ADRHDL--QR LKLICEDR-L
OsMB20b/1-369  TDSL-PKMEE ---------- ---------- ---------E DVMWQHLLVA ADRHDL--QR LKLICEDR-L
OsMB21/1-369   TDSL-PEMET ---------- ---------- --------EE DVVCQHLLVT ADRYDL--HR LKLMCENR-L
OsMB22/1-363   TDSL-PEMKE ---------- ---------- --------EE DVMCQHLLVA ADRYNL--ER LKLICEEK-L
OsMB23/1-356   TDSL-PEMKK ---------- ---------- -------KDE GIMCQHLLVA ADRYNL--ER LKLICEEK-L
OsMB24/1-362   TDDL-PEMKQ ---------- ---------- -------QDE AAMAQHLLVA ADRYNL--ER MKLICEHN-L
OsMB25/1-369   TDSL-PETME ---------- ---------- -------ERE DTMCEHLLVA ADRYNL--ER LKLICEER-L
OsMB26/1-312   TDAL-PEMKQ ---------- ---------- -------QEE AAMAQHLLVA ADRYNL--ER MKLICEDK-L
OsMB27/1-359   TDSL-PEMGN ---------- ---------- -------DDE DVMCQHLLVA ADRYNL--ER LKLIYEEK-L
OsMB28/1-372   TDSLPPEKGT ---------- ---------- ------TREE AAMAQNMIVA ADRYSM--ET LKLMCEDR-L
OsMB29/1-409   TDSL-PEMKE ---------- ---------- -------KDE GAMCQHLLVA ADRYAM--ER LKLVCEEK-L
OsMB30/1-370   NDTPPPETEE ---------- ---------- -----DEDGK VAMWQHLLVA ADRYDL--PR LKLICEEK-L
OsMB31/1-390   TDSL-PETKK ---------- ---------- -------EDE YAMCQHLLVA ADRYNM--ER LKLMCEER-L
OsMB32/1-386   TDSL-PKMKR ---------- ---------- -------RDE AAMAQHLLVA ADRYNL--ER LKLICEDK-L
OsMB33/1-373   TDEL-PETET ---------- ---------- -------KQQ VVMAQHLLVA ADRYGM--QR LMRLCEEK-L
OsMB34/1-366   TDTW-PEIGQ ---------- ---------- --------DE TTMVQQLLVA ANKYSL--SR LKIMCEDK-L
OsMB35/1-363   TDSL-PEMEA ---------- ---------- --------EE DVMCQHLIVA ADRYDL--QR LKLICEEK-L
OsMB36/1-253   TDML-PKMDQ ---------- ---------- -------EDE VAMAQHLLVA SDTYGL--QR LMLICEDR-L
OsMB37/1-359   TDTW-PEIDQ ---------- ---------- --------DE TTMVQ-LLVA ANKYSL--SR LKIMCEDR-L
OsMB38/1-370   TDIW-PEIGH ---------- ---------- ------GEDN VAMAQRLLAA ADMYGL--QR LKFVYEDK-L
OsMB39/1-395   TDML-PEMDQ ---------- ---------- -------EDE TAMVQHLLVA ADKYGL--HR LKMICVEI-L
OsMB40/1-382   TNAL-PETKT ---------- ---------- ---KANQEDE LVIAQHLLVA ADRYGM--ER LKLLCEEK-L
OsMB41/1-383   TDAL-PETKT ---------- ---------- ---KANQEDE LVIAQHLLVA ADRYGM--ER LKLLCEEK-V
OsMB42/1-401   TDEL-PERED HGGEKETSSD DDDDDDDDDN GAQSDQKHKQ FTWLQQLIVA ADRYDL--QR LKLLCEEE-M
OsMB43/1-349   IDML-PEMDQ ---------- ---------- -------EDE AAMAQHLLVA ADKYGL--HR LKMICVEI-L
OsMB44/1-355   TDIW-SEIGH ---------- ---------- ------GKDN VAMAQQLLAA ADRYGL--QR LKFVYEDK-L
OsMB45/1-397   TDTL-LEMDQ ---------- ---------- -------EED ATMAQHLLVA ADKYGL--ER LKVKCEER-L
OsMB46/1-718   TDSLPPQMQQ ---------- ---------- ---GELEEEG RAMAQHLLAA ADRYGM--ER LRLLCEAQ-L
OsMB47/1-305   TDSL-PKMKE ---------- ---------- ---------E DVMCQHLLVA ADRYNL--KR LKLICEKK-L
OsMB48/1-372   TDAL-PETKT ---------- ---------- ---KAKQEDE LVIAQHLLVV ADRYGM--ER LKLLCEEK-V
OsMB49/1-322   TDSL-PEMEE ---------- ---------- --------EE DTMCQHLLVA ADRYNL--ER LKLICEDR-L
OsMB50/1-380   TDTL-PEMAS ---------- ---------- -------ADV PAMARQLIAA ADKYKV--ER LKLVCEDK-L
OsMB51/1-392   TDELPPGELD ---------- ---------- VAGDGDADTA AIMAQHLLVA ADKYDL--PR LKLVCERK-L
OsMB52/1-370   NDSL-PKVDD ---------- ---------- -------DEV VAMAQHLLVA ADRYGM--ER LKLMCEDT-L
OsMB53/1-342   TDTL-PETMA ---------- ---------- ------PREV PAMAPQLIAA ARKYGLDWER LRLLCEDKAW
OsMB54/1-370   TDTL-PKIDK ---------- ---------- -------GDT AFMAQNLLVA AHRYGI--ER LKSISVD--M
```

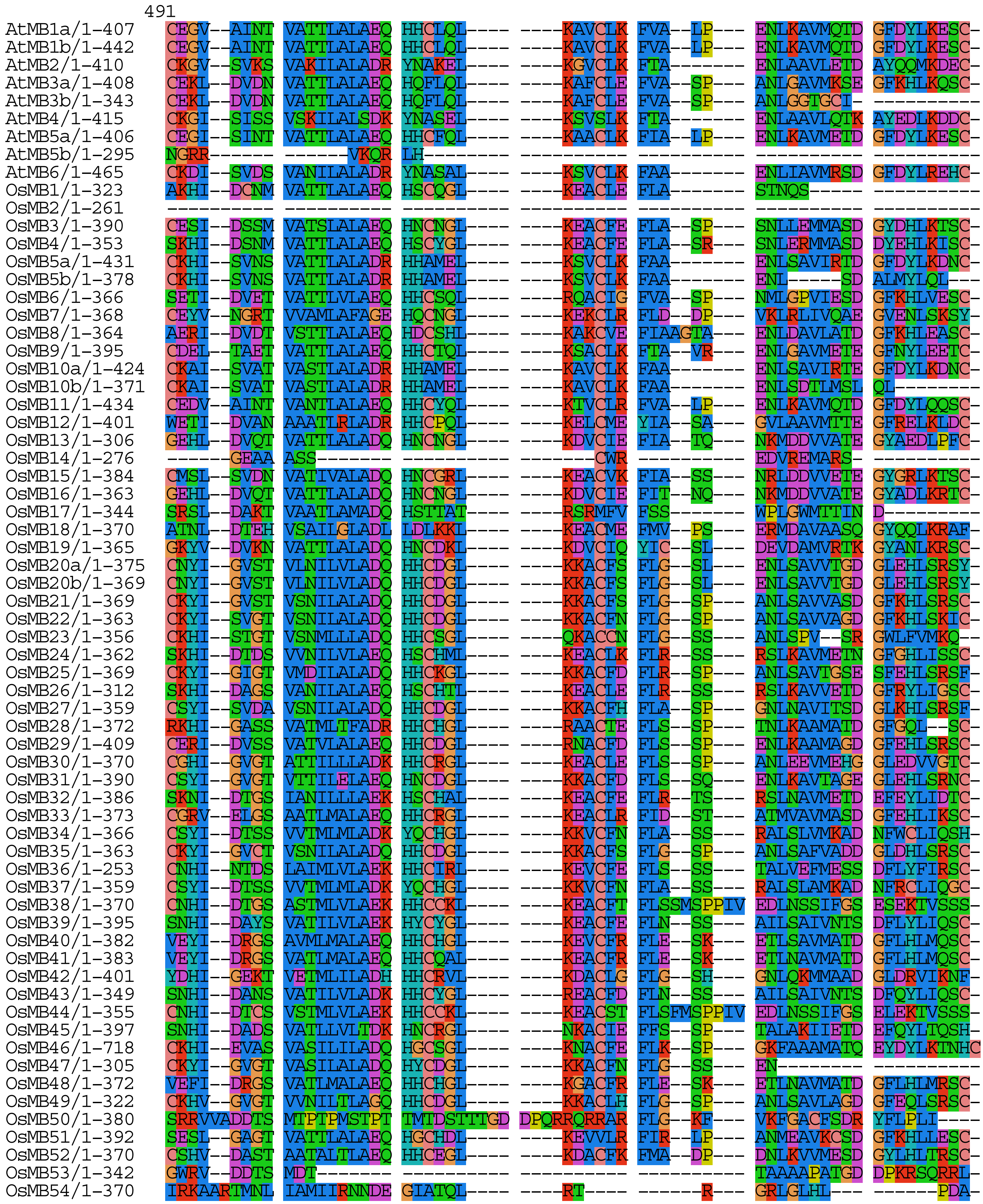
491
AtMB1a/1-407
AtMB1b/1-442
AtMB2/1-410
AtMB3a/1-408
AtMB3b/1-343
AtMB4/1-415
AtMB5a/1-406
AtMB5b/1-295
AtMB6/1-465
OsMB1/1-323
OsMB2/1-261
OsMB3/1-390
OsMB4/1-353
OsMB5a/1-431
OsMB5b/1-378
OsMB6/1-366
OsMB7/1-368
OsMB8/1-364
OsMB9/1-395
OsMB10a/1-424
OsMB10b/1-371
OsMB11/1-434
OsMB12/1-401
OsMB13/1-306
OsMB14/1-276
OsMB15/1-384
OsMB16/1-363
OsMB17/1-344
OsMB18/1-370
OsMB19/1-365
OsMB20a/1-375
OsMB20b/1-369
OsMB21/1-369
OsMB22/1-363
OsMB23/1-356
OsMB24/1-362
OsMB25/1-369
OsMB26/1-312
OsMB27/1-359
OsMB28/1-372
OsMB29/1-409
OsMB30/1-370
OsMB31/1-390
OsMB32/1-386
OsMB33/1-373
OsMB34/1-366
OsMB35/1-363
OsMB36/1-253
OsMB37/1-359
OsMB38/1-370
OsMB39/1-395
OsMB40/1-382
OsMB41/1-383
OsMB42/1-401
OsMB43/1-349
OsMB44/1-355
OsMB45/1-397
OsMB46/1-718
OsMB47/1-305
OsMB48/1-372
OsMB49/1-322
OsMB50/1-380
OsMB51/1-392
OsMB52/1-370
OsMB53/1-342
OsMB54/1-370

```
                  561
AtMB1a/1-407    ---------- ---------- ---------- ---------- ---------- ---------- ----------
AtMB1b/1-442    ---------- ---------- ---------- ---------- ---------- ---------- ----------
AtMB2/1-410     ---------- ---------- ---------- ---------- ---------- ---------- ----------
AtMB3a/1-408    ---------- ---------- ---------- ---------- ---------- ---------- ----------
AtMB3b/1-343    ---------- ---------- ---------- ---------- ---------- ---------- ----------
AtMB4/1-415     ---------- ---------- ---------- ---------- ---------- ---------- ----------
AtMB5a/1-406    ---------- ---------- ---------- ---------- ---------- ---------- ----------
AtMB5b/1-295    ---------- ---------- ---------- ---------- ---------- ---------- ----------
AtMB6/1-465     ---------- ---------- ---------- ---------- ---------- ---------- ----------
OsMB1/1-323     ---------- ---------- ---------- ---------- ---------- ---------- ----------
OsMB2/1-261     ---------- ---------- ---------- ---------- ---------- ---------- ----------
OsMB3/1-390     ---------- ---------- ---------- ---------- ---------- ---------- ----------
OsMB4/1-353     ---------- ---------- ---------- ---------- ---------- ---------- ----------
OsMB5a/1-431    ---------- ---------- ---------- ---------- ---------- ---------- ----------
OsMB5b/1-378    ---------- ---------- ---------- ---------- ---------- ---------- ----------
OsMB6/1-366     ---------- ---------- ---------- ---------- ---------- ---------- ----------
OsMB7/1-368     ---------- ---------- ---------- ---------- ---------- ---------- ----------
OsMB8/1-364     ---------- ---------- ---------- ---------- ---------- ---------- ----------
OsMB9/1-395     ---------- ---------- ---------- ---------- ---------- ---------- ----------
OsMB10a/1-424   ---------- ---------- ---------- ---------- ---------- ---------- ----------
OsMB10b/1-371   ---------- ---------- ---------- ---------- ---------- ---------- ----------
OsMB11/1-434    ---------- ---------- ---------- ---------- ---------- ---------- ----------
OsMB12/1-401    ---------- ---------- ---------- ---------- ---------- ---------- ----------
OsMB13/1-306    ---------- ---------- ---------- ---------- ---------- ---------- ----------
OsMB14/1-276    ---------- ---------- ---------- ---------- ---------- ---------- ----------
OsMB15/1-384    ---------- ---------- ---------- ---------- ---------- ---------- ----------
OsMB16/1-363    ---------- ---------- ---------- ---------- ---------- ---------- ----------
OsMB17/1-344    ---------- ---------- ---------- ---------- ---------- ---------- ----------
OsMB18/1-370    ---------- ---------- ---------- ---------- ---------- ---------- ----------
OsMB19/1-365    ---------- ---------- ---------- ---------- ---------- ---------- ----------
OsMB20a/1-375   ---------- ---------- ---------- ---------- ---------- ---------- ----------
OsMB20b/1-369   ---------- ---------- ---------- ---------- ---------- ---------- ----------
OsMB21/1-369    ---------- ---------- ---------- ---------- ---------- ---------- ----------
OsMB22/1-363    ---------- ---------- ---------- ---------- ---------- ---------- ----------
OsMB23/1-356    ---------- ---------- ---------- ---------- ---------- ---------- ----------
OsMB24/1-362    ---------- ---------- ---------- ---------- ---------- ---------- ----------
OsMB25/1-369    ---------- ---------- ---------- ---------- ---------- ---------- ----------
OsMB26/1-312    ---------- ---------- ---------- ---------- ---------- ---------- ----------
OsMB27/1-359    ---------- ---------- ---------- ---------- ---------- ---------- ----------
OsMB28/1-372    ---------- ---------- ---------- ---------- ---------- ---------- ----------
OsMB29/1-409    ---------- ---------- ---------- ---------- ---------- ---------- ----------
OsMB30/1-370    ---------- ---------- ---------- ---------- ---------- ---------- ----------
OsMB31/1-390    ---------- ---------- ---------- ---------- ---------- ---------- ----------
OsMB32/1-386    ---------- ---------- ---------- ---------- ---------- ---------- ----------
OsMB33/1-373    ---------- ---------- ---------- ---------- ---------- ---------- ----------
OsMB34/1-366    ---------- ---------- ---------- ---------- ---------- ---------- ----------
OsMB35/1-363    ---------- ---------- ---------- ---------- ---------- ---------- ----------
OsMB36/1-253    ---------- ---------- ---------- ---------- ---------- ---------- ----------
OsMB37/1-359    ---------- ---------- ---------- ---------- ---------- ---------- ----------
OsMB38/1-370    ---------- ---------- ---------- ---------- ---------- ---------- ----------
OsMB39/1-395    ---------- ---------- ---------- ---------- ---------- ---------- ----------
OsMB40/1-382    ---------- ---------- ---------- ---------- ---------- ---------- ----------
OsMB41/1-383    ---------- ---------- ---------- ---------- ---------- ---------- ----------
OsMB42/1-401    ---------- ---------- ---------- ---------- ---------- ---------- ----------
OsMB43/1-349    ---------- ---------- ---------- ---------- ---------- ---------- ----------
OsMB44/1-355    ---------- ---------- ---------- ---------- ---------- ---------- ----------
OsMB45/1-397    ---------- ---------- ---------- ---------- ---------- ---------- ----------
OsMB46/1-718    ALADEVVKGN QAKWMLATVE TFMRHRLNSK SILETATREI ALHASTSNET PPVFLDLEES LRAKGDMTLR
OsMB47/1-305    ---------- ---------- ---------- ---------- ---------- ---------- ----------
OsMB48/1-372    ---------- ---------- ---------- ---------- ---------- ---------- ----------
OsMB49/1-322    ---------- ---------- ---------- ---------- ---------- ---------- ----------
OsMB50/1-380    ---------- ---------- ---------- ---------- ---------- ---------- ----------
OsMB51/1-392    ---------- ---------- ---------- ---------- ---------- ---------- ----------
OsMB52/1-370    ---------- ---------- ---------- ---------- ---------- ---------- ----------
OsMB53/1-342    ---------- ---------- ---------- ---------- ---------- ---------- ----------
OsMB54/1-370    ---------- ---------- ---------- ---------- ---------- ---------- ----------
```

```
                  631
AtMB1a/1-407    ---------- ---------- ---------- ---------- ---------- ---------- ----------
AtMB1b/1-442    ---------- ---------- ---------- ---------- ---------- ---------- ----------
AtMB2/1-410     ---------- ---------- ---------- ---------- ---------- ---------- ----------
AtMB3a/1-408    ---------- ---------- ---------- ---------- ---------- ---------- ----------
AtMB3b/1-343    ---------- ---------- ---------- ---------- ---------- ---------- ----------
AtMB4/1-415     ---------- ---------- ---------- ---------- ---------- ---------- ----------
AtMB5a/1-406    ---------- ---------- ---------- ---------- ---------- ---------- ----------
AtMB5b/1-295    ---------- ---------- ---------- ---------- ---------- ---------- ----------
AtMB6/1-465     ---------- ---------- ---------- ---------- ---------- ---------- ----------
OsMB1/1-323     ---------- ---------- ---------- ---------- ---------- ---------- ----------
OsMB2/1-261     ---------- ---------- ---------- ---------- ---------- ---------- ----------
OsMB3/1-390     ---------- ---------- ---------- ---------- ---------- ---------- ----------
OsMB4/1-353     ---------- ---------- ---------- ---------- ---------- ---------- ----------
OsMB5a/1-431    ---------- ---------- ---------- ---------- ---------- ---------- ----------
OsMB5b/1-378    ---------- ---------- ---------- ---------- ---------- ---------- ----------
OsMB6/1-366     ---------- ---------- ---------- ---------- ---------- ---------- ----------
OsMB7/1-368     ---------- ---------- ---------- ---------- ---------- ---------- ----------
OsMB8/1-364     ---------- ---------- ---------- ---------- ---------- ---------- ----------
OsMB9/1-395     ---------- ---------- ---------- ---------- ---------- ---------- ----------
OsMB10a/1-424   ---------- ---------- ---------- ---------- ---------- ---------- ----------
OsMB10b/1-371   ---------- ---------- ---------- ---------- ---------- ---------- ----------
OsMB11/1-434    ---------- ---------- ---------- ---------- ---------- ---------- ----------
OsMB12/1-401    ---------- ---------- ---------- ---------- ---------- ---------- ----------
OsMB13/1-306    ---------- ---------- ---------- ---------- ---------- ---------- ----------
OsMB14/1-276    ---------- ---------- ---------- ---------- ---------- ---------- ----------
OsMB15/1-384    ---------- ---------- ---------- ---------- ---------- ---------- ----------
OsMB16/1-363    ---------- ---------- ---------- ---------- ---------- ---------- ----------
OsMB17/1-344    ---------- ---------- ---------- ---------- ---------- ---------- ----------
OsMB18/1-370    ---------- ---------- ---------- ---------- ---------- ---------- ----------
OsMB19/1-365    ---------- ---------- ---------- ---------- ---------- ---------- ----------
OsMB20a/1-375   ---------- ---------- ---------- ---------- ---------- ---------- ----------
OsMB20b/1-369   ---------- ---------- ---------- ---------- ---------- ---------- ----------
OsMB21/1-369    ---------- ---------- ---------- ---------- ---------- ---------- ----------
OsMB22/1-363    ---------- ---------- ---------- ---------- ---------- ---------- ----------
OsMB23/1-356    ---------- ---------- ---------- ---------- ---------- ---------- ----------
OsMB24/1-362    ---------- ---------- ---------- ---------- ---------- ---------- ----------
OsMB25/1-369    ---------- ---------- ---------- ---------- ---------- ---------- ----------
OsMB26/1-312    ---------- ---------- ---------- ---------- ---------- ---------- ----------
OsMB27/1-359    ---------- ---------- ---------- ---------- ---------- ---------- ----------
OsMB28/1-372    ---------- ---------- ---------- ---------- ---------- ---------- ----------
OsMB29/1-409    ---------- ---------- ---------- ---------- ---------- ---------- ----------
OsMB30/1-370    ---------- ---------- ---------- ---------- ---------- ---------- ----------
OsMB31/1-390    ---------- ---------- ---------- ---------- ---------- ---------- ----------
OsMB32/1-386    ---------- ---------- ---------- ---------- ---------- ---------- ----------
OsMB33/1-373    ---------- ---------- ---------- ---------- ---------- ---------- ----------
OsMB34/1-366    ---------- ---------- ---------- ---------- ---------- ---------- ----------
OsMB35/1-363    ---------- ---------- ---------- ---------- ---------- ---------- ----------
OsMB36/1-253    ---------- ---------- ---------- ---------- ---------- ---------- ----------
OsMB37/1-359    ---------- ---------- ---------- ---------- ---------- ---------- ----------
OsMB38/1-370    ---------- ---------- ---------- ---------- ---------- ---------- ----------
OsMB39/1-395    ---------- ---------- ---------- ---------- ---------- ---------- ----------
OsMB40/1-382    ---------- ---------- ---------- ---------- ---------- ---------- ----------
OsMB41/1-383    ---------- ---------- ---------- ---------- ---------- ---------- ----------
OsMB42/1-401    ---------- ---------- ---------- ---------- ---------- ---------- ----------
OsMB43/1-349    ---------- ---------- ---------- ---------- ---------- ---------- ----------
OsMB44/1-355    ---------- ---------- ---------- ---------- ---------- ---------- ----------
OsMB45/1-397    ---------- ---------- ---------- ---------- ---------- ---------- ----------
OsMB46/1-718    EEATLKVLKV VATLGKAIST GAIAAVGYYV PGIVGAKFGE PSLPRLPRFA MAAVTSWFAG KVMYYAILQG
OsMB47/1-305    ---------- ---------- ---------- ---------- ---------- ---------- ----------
OsMB48/1-372    ---------- ---------- ---------- ---------- ---------- ---------- ----------
OsMB49/1-322    ---------- ---------- ---------- ---------- ---------- ---------- ----------
OsMB50/1-380    ---------- ---------- ---------- ---------- ---------- ---------- ----------
OsMB51/1-392    ---------- ---------- ---------- ---------- ---------- ---------- ----------
OsMB52/1-370    ---------- ---------- ---------- ---------- ---------- ---------- ----------
OsMB53/1-342    ---------- ---------- ---------- ---------- ---------- ---------- ----------
OsMB54/1-370    ---------- ---------- ---------- ---------- ---------- ---------- ----------
```

```
                   701
AtMB1a/1-407       ---------- ---------- ---------- ---------- ---------- ---------- ----------
AtMB1b/1-442       ---------- ---------- ---------- ---------- ---------- ---------- ----------
AtMB2/1-410        ---------- ---------- ---------- ---------- ---------- ---------- ----------
AtMB3a/1-408       ---------- ---------- ---------- ---------- ---------- ---------- ----------
AtMB3b/1-343       ---------- ---------- ---------- ---------- ---------- ---------- ----------
AtMB4/1-415        ---------- ---------- ---------- ---------- ---------- ---------- ----------
AtMB5a/1-406       ---------- ---------- ---------- ---------- ---------- ---------- ----------
AtMB5b/1-295       ---------- ---------- ---------- ---------- ---------- ---------- ----------
AtMB6/1-465        ---------- ---------- ---------- ---------- ---------- ---------- ----------
OsMB1/1-323        ---------- ---------- ---------- ---------- ---------- ---------- ----------
OsMB2/1-261        ---------- ---------- ---------- ---------- ---------- ---------- ----------
OsMB3/1-390        ---------- ---------- ---------- ---------- ---------- ---------- ----------
OsMB4/1-353        ---------- ---------- ---------- ---------- ---------- ---------- ----------
OsMB5a/1-431       ---------- ---------- ---------- ---------- ---------- ---------- ----------
OsMB5b/1-378       ---------- ---------- ---------- ---------- ---------- ---------- ----------
OsMB6/1-366        ---------- ---------- ---------- ---------- ---------- ---------- ----------
OsMB7/1-368        ---------- ---------- ---------- ---------- ---------- ---------- ----------
OsMB8/1-364        ---------- ---------- ---------- ---------- ---------- ---------- ----------
OsMB9/1-395        ---------- ---------- ---------- ---------- ---------- ---------- ----------
OsMB10a/1-424      ---------- ---------- ---------- ---------- ---------- ---------- ----------
OsMB10b/1-371      ---------- ---------- ---------- ---------- ---------- ---------- ----------
OsMB11/1-434       ---------- ---------- ---------- ---------- ---------- ---------- ----------
OsMB12/1-401       ---------- ---------- ---------- ---------- ---------- ---------- ----------
OsMB13/1-306       ---------- ---------- ---------- ---------- ---------- ---------- ----------
OsMB14/1-276       ---------- ---------- ---------- ---------- ---------- ---------- ----------
OsMB15/1-384       ---------- ---------- ---------- ---------- ---------- ---------- ----------
OsMB16/1-363       ---------- ---------- ---------- ---------- ---------- ---------- ----------
OsMB17/1-344       ---------- ---------- ---------- ---------- ---------- ---------- ----------
OsMB18/1-370       ---------- ---------- ---------- ---------- ---------- ---------- ----------
OsMB19/1-365       ---------- ---------- ---------- ---------- ---------- ---------- ----------
OsMB20a/1-375      ---------- ---------- ---------- ---------- ---------- ---------- ----------
OsMB20b/1-369      ---------- ---------- ---------- ---------- ---------- ---------- ----------
OsMB21/1-369       ---------- ---------- ---------- ---------- ---------- ---------- ----------
OsMB22/1-363       ---------- ---------- ---------- ---------- ---------- ---------- ----------
OsMB23/1-356       ---------- ---------- ---------- ---------- ---------- ---------- ----------
OsMB24/1-362       ---------- ---------- ---------- ---------- ---------- ---------- ----------
OsMB25/1-369       ---------- ---------- ---------- ---------- ---------- ---------- ----------
OsMB26/1-312       ---------- ---------- ---------- ---------- ---------- ---------- ----------
OsMB27/1-359       ---------- ---------- ---------- ---------- ---------- ---------- ----------
OsMB28/1-372       ---------- ---------- ---------- ---------- ---------- ---------- ----------
OsMB29/1-409       ---------- ---------- ---------- ---------- ---------- ---------- ----------
OsMB30/1-370       ---------- ---------- ---------- ---------- ---------- ---------- ----------
OsMB31/1-390       ---------- ---------- ---------- ---------- ---------- ---------- ----------
OsMB32/1-386       ---------- ---------- ---------- ---------- ---------- ---------- ----------
OsMB33/1-373       ---------- ---------- ---------- ---------- ---------- ---------- ----------
OsMB34/1-366       ---------- ---------- ---------- ---------- ---------- ---------- ----------
OsMB35/1-363       ---------- ---------- ---------- ---------- ---------- ---------- ----------
OsMB36/1-253       ---------- ---------- ---------- ---------- ---------- ---------- ----------
OsMB37/1-359       ---------- ---------- ---------- ---------- ---------- ---------- ----------
OsMB38/1-370       ---------- ---------- ---------- ---------- ---------- ---------- ----------
OsMB39/1-395       ---------- ---------- ---------- ---------- ---------- ---------- ----------
OsMB40/1-382       ---------- ---------- ---------- ---------- ---------- ---------- ----------
OsMB41/1-383       ---------- ---------- ---------- ---------- ---------- ---------- ----------
OsMB42/1-401       ---------- ---------- ---------- ---------- ---------- ---------- ----------
OsMB43/1-349       ---------- ---------- ---------- ---------- ---------- ---------- ----------
OsMB44/1-355       ---------- ---------- ---------- ---------- ---------- ---------- ----------
OsMB45/1-397       ---------- ---------- ---------- ---------- ---------- ---------- ----------
OsMB46/1-718       STEFILKHGE ERMKMELSNI ILNKHSDDKT LVEAVKTQFF AEHLFSDQYQ DRTLFRWHLR RTYVDSAFME
OsMB47/1-305       ---------- ---------- ---------- ---------- ---------- ---------- ----------
OsMB48/1-372       ---------- ---------- ---------- ---------- ---------- ---------- ----------
OsMB49/1-322       ---------- ---------- ---------- ---------- ---------- ---------- ----------
OsMB50/1-380       ---------- ---------- ---------- ---------- ---------- ---------- ----------
OsMB51/1-392       ---------- ---------- ---------- ---------- ---------- ---------- ----------
OsMB52/1-370       ---------- ---------- ---------- ---------- ---------- ---------- ----------
OsMB53/1-342       ---------- ---------- ---------- ---------- ---------- ---------- ----------
OsMB54/1-370       ---------- ---------- ---------- ---------- ---------- ---------- ----------
```

```
                 771
AtMB1a/1-407     ---------- ---------- ---------- ---------- ---------- ---------- --------PS
AtMB1b/1-442     ---------- ---------- ---------- ---------- ---------- ---------- --------PS
AtMB2/1-410      ---------- ---------- ---------- ---------- ---------- ---------- --------VT
AtMB3a/1-408     ---------- ---------- ---------- ---------- ---------- ---------- --------PT
AtMB3b/1-343     ---------- ---------- ---------- ---------- ---------- ---------- ----------
AtMB4/1-415      ---------- ---------- ---------- ---------- ---------- ---------- --------PN
AtMB5a/1-406     ---------- ---------- ---------- ---------- ---------- ---------- --------PS
AtMB5b/1-295     ---------- ---------- ---------- ---------- ---------- ---------- ----------
AtMB6/1-465      ---------- ---------- ---------- ---------- ---------- ---------- --------PS
OsMB1/1-323      ---------- ---------- ---------- ---------- ---------- ---------- ----------
OsMB2/1-261      ---------- ---------- ---------- ---------- ---------- ---------- ----------
OsMB3/1-390      ---------- ---------- ---------- ---------- ---------- ---------- --------PA
OsMB4/1-353      ---------- ---------- ---------- ---------- ---------- ---------- --------PS
OsMB5a/1-431     ---------- ---------- ---------- ---------- ---------- ---------- --------PA
OsMB5b/1-378     ---------- ---------- ---------- ---------- ---------- ---------- ----------
OsMB6/1-366      ---------- ---------- ---------- ---------- ---------- ---------- --------PL
OsMB7/1-368      ---------- ---------- ---------- ---------- ---------- ---------- --------PS
OsMB8/1-364      ---------- ---------- ---------- ---------- ---------- ---------- --------PS
OsMB9/1-395      ---------- ---------- ---------- ---------- ---------- ---------- --------PS
OsMB10a/1-424    ---------- ---------- ---------- ---------- ---------- ---------- --------PS
OsMB10b/1-371    ---------- ---------- ---------- ---------- ---------- ---------- ----------
OsMB11/1-434     ---------- ---------- ---------- ---------- ---------- ---------- --------PS
OsMB12/1-401     ---------- ---------- ---------- ---------- ---------- ---------- --------PS
OsMB13/1-306     ---------- ---------- ---------- ---------- ---------- ---------- --------LS
OsMB14/1-276     ---------- ---------- ---------- ---------- ---------- ---------- ----------
OsMB15/1-384     ---------- ---------- ---------- ---------- ---------- ---------- --------PS
OsMB16/1-363     ---------- ---------- ---------- ---------- ---------- ---------- --------PS
OsMB17/1-344     ---------- ---------- ---------- ---------- ---------- ---------- ----------
OsMB18/1-370     ---------- ---------- ---------- ---------- ---------- ---------- --------PS
OsMB19/1-365     ---------- ---------- ---------- ---------- ---------- ---------- --------PS
OsMB20a/1-375    ---------- ---------- ---------- ---------- ---------- ---------- --------PS
OsMB20b/1-369    ---------- ---------- ---------- ---------- ---------- ---------- --------PS
OsMB21/1-369     ---------- ---------- ---------- ---------- ---------- ---------- --------PS
OsMB22/1-363     ---------- ---------- ---------- ---------- ---------- ---------- --------PS
OsMB23/1-356     ---------- ---------- ---------- ---------- ---------- ---------- ----------
OsMB24/1-362     ---------- ---------- ---------- ---------- ---------- ---------- --------PG
OsMB25/1-369     ---------- ---------- ---------- ---------- ---------- ---------- --------PS
OsMB26/1-312     ---------- ---------- ---------- ---------- ---------- ---------- --------PG
OsMB27/1-359     ---------- ---------- ---------- ---------- ---------- ---------- --------PS
OsMB28/1-372     ---------- ---------- ---------- ---------- ---------- ---------- --------PT
OsMB29/1-409     ---------- ---------- ---------- ---------- ---------- ---------- --------PS
OsMB30/1-370     ---------- ---------- ---------- ---------- ---------- ---------- --------PS
OsMB31/1-390     ---------- ---------- ---------- ---------- ---------- ---------- --------PS
OsMB32/1-386     ---------- ---------- ---------- ---------- ---------- ---------- --------PG
OsMB33/1-373     ---------- ---------- ---------- ---------- ---------- ---------- --------PS
OsMB34/1-366     ---------- ---------- ---------- ---------- ---------- ---------- --------PT
OsMB35/1-363     ---------- ---------- ---------- ---------- ---------- ---------- --------PS
OsMB36/1-253     ---------- ---------- ---------- ---------- ---------- ---------- --------PT
OsMB37/1-359     ---------- ---------- ---------- ---------- ---------- ---------- --------PT
OsMB38/1-370     ---------- ---------- ---------- ---------- ---------- ---------- --------DN
OsMB39/1-395     ---------- ---------- ---------- ---------- ---------- ---------- --------PD
OsMB40/1-382     ---------- ---------- ---------- ---------- ---------- ---------- --------PS
OsMB41/1-383     ---------- ---------- ---------- ---------- ---------- ---------- --------PS
OsMB42/1-401     ---------- ---------- ---------- ---------- ---------- ---------- --------PS
OsMB43/1-349     ---------- ---------- ---------- ---------- ---------- ---------- --------PD
OsMB44/1-355     ---------- ---------- ---------- ---------- ---------- ---------- --------EN
OsMB45/1-397     ---------- ---------- ---------- ---------- ---------- ---------- --------PN
OsMB46/1-718     RVKEIEVKSS NDGPGLISGQ RIISTRPFGD LMEDPLACIL GSPDSNMESN KSAEHTGTIV KRREIACPPA
OsMB47/1-305     ---------- ---------- ---------- ---------- ---------- ---------- --------PP
OsMB48/1-372     ---------- ---------- ---------- ---------- ---------- ---------- --------PS
OsMB49/1-322     ---------- ---------- ---------- ---------- ---------- ---------- --------PS
OsMB50/1-380     ---------- ---------- ---------- ---------- ---------- ---------- ----------
OsMB51/1-392     ---------- ---------- ---------- ---------- ---------- ---------- --------PS
OsMB52/1-370     ---------- ---------- ---------- ---------- ---------- ---------- --------SY
OsMB53/1-342     ---------- ---------- ---------- ---------- ---------- ---------- --------PW
OsMB54/1-370     ---------- ---------- ---------- ---------- ---------- ---------- --------PE
```

```
               841
AtMB1a/1-407   LLTELL---Q YVARLSEHSV IVSGHRKEIF ADGCDASGRR VKPRLH---- ---------- ----------
AtMB1b/1-442   LLTELL---Q YVARLSEHSV IVSGHRKEIF ADGCDASGRR VKPRLH---- ---------- ----------
AtMB2/1-410    LQSELL---K AVAGHEEGSN STGGAKSQSV WAQLSDGGGD TTSRHVRQRT T--------- ----------
AtMB3a/1-408   LLSELL---N TVAAADKSST SGQSNKKRSA SSVLGCDTTN VRQLRRRTRK EVRAVS---- ----------
AtMB3b/1-343   ---------- ---------- ---------- ---------- ---------- ---------- ----------
AtMB4/1-415    LQSELL---K AVAGYDDTSS SGGGKSQSVW AQLSNGGETS SRRVRQRTT- ---------- ----------
AtMB5a/1-406   LLSELL---E YVARLSEHSL TSSGHRKELF ADGCDLNGRR VKQRLH---- ---------- ----------
AtMB5b/1-295   ---------- ---------- ---------- ---------- ---------- ---------- ----------
AtMB6/1-465    LQSELL---K TVAGCEEELS GGGGKTRSVW GQFSDGGAET NGRQAQTWGD INGGAERSQS VWVEVVNANG
OsMB1/1-323    ---------- ---------- ---------- ---------- ---------- ---------- ----------
OsMB2/1-261    ---------- ---------- ---------- ---------- ---------- ---------- ----------
OsMB3/1-390    VLKELT---T RFLPPETKAS EEITIGLYN- ---------- ---------- ---------- ----------
OsMB4/1-353    VLMELV---A RFLPQQEK-- ---------- ---------- ---------- ---------- ----------
OsMB5a/1-431   LQSEIL---R TVAGCEEECS SGGKSQSVWG QLSDGGDTSG RRVRPRV--- ---------- ----------
OsMB5b/1-378   ---------- ---------- ---------- ---------- ---------- ---------- ----------
OsMB6/1-366    IMKEIL---S KVSHIWIDKS C--------- ---------- ---------- ---------- ----------
OsMB7/1-368    ILKDVI---A KFVATPVDS- ---------- ---------- ---------- ---------- ----------
OsMB8/1-364    VLTDLV---- KVARGRKN-- ---------- ---------- ---------- ---------- ----------
OsMB9/1-395    LLSDLL---A TVAVVDDDAA SFNRKRGVGG NEGANPVESV EASDRRIRRR V--------- ----------
OsMB10a/1-424  LQSEIL---R TLAGCEEECS SGGKSQSVWG QLSDGGDTSG RRVRPRT--- ---------- ----------
OsMB10b/1-371  ---------- ---------- ---------- ---------- ---------- ---------- ----------
OsMB11/1-434   LLTELL---E YVAKVGEHSV SPCLYSNEVL -DGGDANGRR VKPRI----- ---------- ----------
OsMB12/1-401   LLIEIL---E NFGKRSEADE E--------- ---------- ---------- ---------- ----------
OsMB13/1-306   ---------- ---------- ---------- ---------- ---------- ---------- ----------
OsMB14/1-276   ---------- ---------- ---------- ---------- ---------- ---------- ----------
OsMB15/1-384   ILLDVI---E MATKSRKIN- ---------- ---------- ---------- ---------- ----------
OsMB16/1-363   VLVDVF---E KASKFRRI-- ---------- ---------- ---------- ---------- ----------
OsMB17/1-344   ---------- ---------- ---------- ---------- ---------- ---------- ----------
OsMB18/1-370   LAFEVW---E RRSRVRRN-- ---------- ---------- ---------- ---------- ----------
OsMB19/1-365   VLADLF---E KTSKFRAS-- ---------- ---------- ---------- ---------- ----------
OsMB20a/1-375  LMKELF---V VMALPPNHGR YVIFF----- ---------- ---------- ---------- ----------
OsMB20b/1-369  LMKELF---V VMALPPNHG- ---------- ---------- ---------- ---------- ----------
OsMB21/1-369   LMEELV---V MLALPPSHA- ---------- ---------- ---------- ---------- ----------
OsMB22/1-363   LMEELV---V VLALPGSS-- ---------- ---------- ---------- ---------- ----------
OsMB23/1-356   ---------L VLGCWCLH-- ---------- ---------- ---------- ---------- ----------
OsMB24/1-362   LIKDIM---S KLSPC----- ---------- ---------- ---------- ---------- ----------
OsMB25/1-369   LMKELV---D ILGTSHNYAW ---------- ---------- ---------- ---------- ----------
OsMB26/1-312   LIKDIF---S KLSPR----- ---------- ---------- ---------- ---------- ----------
OsMB27/1-359   LMEELV---A MLAPLLSHAL VI-------- ---------- ---------- ---------- ----------
OsMB28/1-372   VLKELM---A KALF------ ---------- ---------- ---------- ---------- ----------
OsMB29/1-409   LMTELV---A MLGNCHDPQP THRSMPNTGS NR-------- ---------- ---------- ----------
OsMB30/1-370   VLVELI---A KLALLRTQV- ---------- ---------- ---------- ---------- ----------
OsMB31/1-390   LVNELI---G TLGNLIQ--- ---------- ---------- ---------- ---------- ----------
OsMB32/1-386   VIKELM---S KLIVNLGQSN EWRRDGALVI PIAKLILRYY QVQ------- ---------- ----------
OsMB33/1-373   LVKELIVRGS QLLSGAK--- ---------- ---------- ---------- ---------- ----------
OsMB34/1-366   MLKDLI---Y NIVTHQLEIK LSV------- ---------- ---------- ---------- ----------
OsMB35/1-363   LMKELV---V ELALPPSHA- ---------- ---------- ---------- ---------- ----------
OsMB36/1-253   VLKDLI---Y NVAAHGK--- ---------- ---------- ---------- ---------- ----------
OsMB37/1-359   MLKDLI---Y NIVTHQLEIK LSV------- ---------- ---------- ---------- ----------
OsMB38/1-370   HGSQINRIDI RIQPWQNADV ---------- ---------- ---------- ---------- ----------
OsMB39/1-395   VLEDIS---F NIVARQLERA IFLSENQEGQ INSVEIRINS VMTN------ ---------- ----------
OsMB40/1-382   LVKELL---F RVVDHSLEIT HVVRL----- ---------- ---------- ---------- ----------
OsMB41/1-383   LVKDLV---F RVADRHFQ-- ---------- ---------- ---------- ---------- ----------
OsMB42/1-401   LTKEII---G RFAVVMANKS ---------- ---------- ---------- ---------- ----------
OsMB43/1-349   ILEDIS---F NIVARQLERA IFLSENQAGQ INSVEIGIRS ---------- ---------- ----------
OsMB44/1-355   HGSQINRTDI RIQPWQNADV ---------- ---------- ---------- ---------- ----------
OsMB45/1-397   ILEDII---S NIVASQLEKA IFSPENEGGK INKVDIRIQP WQNSNARCG- ---------- ----------
OsMB46/1-718   VLVEPIRLQP NVPICSLLAR SRTGSNLEVN PVILDLEESL LAKGNMTLEE EVTIRVSKMT ASVFKVFATA
OsMB47/1-305   VASII----- ---------- ---------- ---------- ---------- ---------- ----------
OsMB48/1-372   LVKDLV---F RVADSHFQ-- ---------- ---------- ---------- ---------- ----------
OsMB49/1-322   LVNELV---A MLAPPIPPSS HA-------- ---------- ---------- ---------- ----------
OsMB50/1-380   ---------- ---------- ---------- ---------- ---------- ---------- ----------
OsMB51/1-392   LHQDLK---S RHIIS----- ---------- ---------- ---------- ---------- ----------
OsMB52/1-370   VLKKLAT--- ---------- ---------- ---------- ---------- ---------- ----------
OsMB53/1-342   FIRKCF---- ---------- ---------- ---------- ---------- ---------- ----------
OsMB54/1-370   VNRAQI---F DTGRG----- ---------- ---------- ---------- ---------- ----------
```

```
                911
AtMB1a/1-407    ---------- ---------- -----
AtMB1b/1-442    ---------- ---------- -----
AtMB2/1-410     ---------- ---------- -----
AtMB3a/1-408    ---------- ---------- -----
AtMB3b/1-343    ---------- ---------- -----
AtMB4/1-415     ---------- ---------- -----
AtMB5a/1-406    ---------- ---------- -----
AtMB5b/1-295    ---------- ---------- -----
AtMB6/1-465     SGRNNNDNNN SDDPMAELED -----
OsMB1/1-323     ---------- ---------- -----
OsMB2/1-261     ---------- ---------- -----
OsMB3/1-390     ---------- ---------- -----
OsMB4/1-353     ---------- ---------- -----
OsMB5a/1-431    ---------- ---------- -----
OsMB5b/1-378    ---------- ---------- -----
OsMB6/1-366     ---------- ---------- -----
OsMB7/1-368     ---------- ---------- -----
OsMB8/1-364     ---------- ---------- -----
OsMB9/1-395     ---------- ---------- -----
OsMB10a/1-424   ---------- ---------- -----
OsMB10b/1-371   ---------- ---------- -----
OsMB11/1-434    ---------- ---------- -----
OsMB12/1-401    ---------- ---------- -----
OsMB13/1-306    ---------- ---------- -----
OsMB14/1-276    ---------- ---------- -----
OsMB15/1-384    ---------- ---------- -----
OsMB16/1-363    ---------- ---------- -----
OsMB17/1-344    ---------- ---------- -----
OsMB18/1-370    ---------- ---------- -----
OsMB19/1-365    ---------- ---------- -----
OsMB20a/1-375   ---------- ---------- -----
OsMB20b/1-369   ---------- ---------- -----
OsMB21/1-369    ---------- ---------- -----
OsMB22/1-363    ---------- ---------- -----
OsMB23/1-356    ---------- ---------- -----
OsMB24/1-362    ---------- ---------- -----
OsMB25/1-369    ---------- ---------- -----
OsMB26/1-312    ---------- ---------- -----
OsMB27/1-359    ---------- ---------- -----
OsMB28/1-372    ---------- ---------- -----
OsMB29/1-409    ---------- ---------- -----
OsMB30/1-370    ---------- ---------- -----
OsMB31/1-390    ---------- ---------- -----
OsMB32/1-386    ---------- ---------- -----
OsMB33/1-373    ---------- ---------- -----
OsMB34/1-366    ---------- ---------- -----
OsMB35/1-363    ---------- ---------- -----
OsMB36/1-253    ---------- ---------- -----
OsMB37/1-359    ---------- ---------- -----
OsMB38/1-370    ---------- ---------- -----
OsMB39/1-395    ---------- ---------- -----
OsMB40/1-382    ---------- ---------- -----
OsMB41/1-383    ---------- ---------- -----
OsMB42/1-401    ---------- ---------- -----
OsMB43/1-349    ---------- ---------- -----
OsMB44/1-355    ---------- ---------- -----
OsMB45/1-397    ---------- ---------- -----
OsMB46/1-718    AFGASSYYAL GLGELSLHAR ARLDL
OsMB47/1-305    ---------- ---------- -----
OsMB48/1-372    ---------- ---------- -----
OsMB49/1-322    ---------- ---------- -----
OsMB50/1-380    ---------- ---------- -----
OsMB51/1-392    ---------- ---------- -----
OsMB52/1-370    ---------- ---------- -----
OsMB53/1-342    ---------- ---------- -----
OsMB54/1-370    ---------- ---------- -----
```
