# Supplementary material for: MATH-Domain Family Shows Response toward Abiotic Stress in Arabidopsis and Rice
Source: Front Plant Sci. 2016 Jun 28;7:923. doi: 10.3389/fpls.2016.00923 (PMC4923191; doi:10.3389/fpls.2016.00923)
Supplement: Table S3 — List of libraries of various abiotic stresses with their abbreviations used in the expression analysis of MDC protein encoding genes in (a) rice and (b) Arabidopsis. [file Table3.DOCX]

| **Table S3: List of libraries of different developmental stages with their abbreviation used in the expression analysis of MDC protein encoding genes in (a) rice and (b) Arabidopsis.** | | | | | |
| --- | --- | --- | --- | --- | --- |
|  | | | | |  |
| **(A) Rice** |  |  | **(B) Arabidopsis** |  |  |
|  |  |  |  |  |  |
| **Full Name** | **Abbreviation** |  | **Full name** | **Abbreviation** |  |
| germination | GRM |  | germinated seed | GMS |  |
| seedling | SDL |  | seedling | SDL |  |
| tillering stage | TLR |  | young rosette | YRT |  |
| stem elongation stage | ELN |  | developed rosette | DRT |  |
| booting stage | BOT |  | bolting | BLT |  |
| heading stage | HED |  | young flower | YFL |  |
| flowering stage | FLW |  | developed flower | DFL |  |
| milk stage | MLK |  | flowers and siliques | FAS |  |
| dough stage | DUG |  | mature siliques | MSQ |  |
|  |  |  | senescence | SNS |  |
|  |  |  |  |  |  |
