# Supplementary material for: MATH-Domain Family Shows Response toward Abiotic Stress in Arabidopsis and Rice
Source: Front Plant Sci. 2016 Jun 28;7:923. doi: 10.3389/fpls.2016.00923 (PMC4923191; doi:10.3389/fpls.2016.00923)
Supplement: Table S5 — List of primers used for qRT-PCR analysis in the present study. [file Table5.DOCX]

| **Table S5: List of primers used for qRT-PCR analysis in the present study** | | | |
| --- | --- | --- | --- |
| **Gene name** | **Locus** | **Forward Primer Sequence** | **Reverse Primer Sequence** |
| OsM1 | Os01g56800 | TCGGTCCGACTTGAGATAGC | GATTGCTGGTGGAATTTGGCT |
| OsM2 | Os01g56490 | AGGTTCCAACACCACCCATC | CGGAGAGGGGGAAAGAAACC |
| OsM4 | Os05g43280 | GGTAGGGTTTACTCGGGCG | GAAGGAGAACAGAGCCACCC |
| OsM5 | Os07g06950 | CGTAACAAGGCCAATTCCCG | TGGAATGATCCACCCGCATT |
| OsM12 | Os12g40520 | TGCGCCGCGTAATCTATCAT | GAGCAACCATCCCGATCCAA |
| OsM13 | Os12g30540 | TGGAAGTAGCCGATCCGTGA | ATACGTATTCGGGAAACACGC |
| OsMB5 | Os03g57854 | TCATCTCAAATCAGGCGGCA | CGAGGCGAGCTAAAGCCTAA |
| OsMB6 | Os04g53410 | TAGTGGCACAGGCTCTCTCA | CGGCCCAAAACTGCAGAATC |
| OsMB9 | Os07g01140 | AGGGGCCGCCATTGTTATAG | CGACCAAGGGTTCATTGGGT |
| OsMB10 | Os07g07270 | TAACTTGTCCTCCAGAACCGC | GCCTCACCTATCCAACAGGC |
| OsMB11 | Os07g46160 | GCGCAAAGACACTCTCTGAAC | ATCACACGGCCAGCTGATTC |
| eEF1α |  | TTTCACTCTTGGTGTGAAGCAGAT | GACTTCCTTCACGATTTCATCGTAA |
